# Long-Term Exposure to Nitrogen Dioxide and Ozone and Mortality: Update of the WHO Air Quality Guidelines Systematic Review and Meta-Analysis

**DOI:** 10.3389/ijph.2024.1607676

**Published:** 2024-10-18

**Authors:** Maria-Iosifina Kasdagli, Pablo Orellano, Román Pérez Velasco, Evangelia Samoli

**Affiliations:** ^1^ Department of Hygiene, Epidemiology and Medical Statistics, Medical School, National and Kapodistrian University of Athens, Athens, Greece; ^2^ Consejo Nacional de Investigaciones Cientificas y Tecnicas (CONICET), Universidad Tecnologica Nacional, Facultad Regional San Nicolas, San Nicolas, Argentina; ^3^ World Health Organization (WHO) Regional Office for Europe, European Centre for Environment and Health, Bonn, Germany

**Keywords:** long-term exposure, meta-analysis, mortality, nitrogen dioxide, ozone

## Abstract

**Objectives:**

We performed a systematic review and meta-analysis on long-term exposure to nitrogen dioxide (NO_2_) and ozone (O_3_) with mortality, to expand evidence that informed 2021 the WHO Air Quality Guidelines and guide the Health Risks of Air Pollution in Europe project.

**Methods:**

We included cohorts investigating NO_2_ and O_3_ mortality from all-causes, respiratory diseases, chronic obstructive pulmonary disease (COPD), acute lower respiratory infections (ALRI); and NO_2_ mortality from circulatory, ischemic heart, cerebrovascular diseases and lung cancer. We pooled estimates by random-effects models and investigated heterogeneity. We assessed the certainty of the evidence using the Grading of Recommendations Assessment Development approach and Evaluation (GRADE).

**Results:**

We selected 83 studies for NO_2_ and 26 for O_3_ for the meta-analysis. NO_2_ was associated with all outcomes, except for cerebrovascular mortality. O_3_ was associated with respiratory mortality following annual exposure. There was high heterogeneity, partly explained by region and pollutant levels. Certainty was high for NO_2_ with COPD and ALRI, and annual O_3_ with respiratory mortality.

**Conclusion:**

An increasing body of evidence, with new results from countrywide areas and the Western Pacific, supports certainty, including new outcomes.

## Introduction

In 2012-2013, the WHO Regional Office for Europe, through its European Centre for Environment and Health (ECEH), coordinated the Health Risks of Air Pollution in Europe (HRAPIE) project to provide advice on suitable concentration-response functions (CRFs) and associated information for health risk assessment. Most recently, WHO ECEH coordinated the update of the WHO global air quality guidelines (AQGs) [1]. Two systematic reviews and meta-analysis on particles [2] and gases [3] health effects were part of the evidence that guided the AQG revision. With new evidence on the health effects of air pollution rapidly accumulating, an update of the HRAPIE project was prioritized by the Joint WHO/Convention Task Force on the Health Aspects of Air Pollution under the United Nations Economic Commission for Europe Convention on Long-range Transboundary Air Pollution.

Under this framework, two systematic reviews and meta-analyses were conducted for updating and expanding the evidence used in the original WHO AQG reviews [2, 3], assessing the health effects of long-term exposure to ambient particles (PM) and gases [nitrogen dioxide (NO_2_) and ozone (O_3_)] and for which the search timeline extended up to September/October 2018.

We present the results on the association between long-term exposure to gases (NO_2_ and O_3_) with mortality, expanded to include additional mortality outcomes not previously assessed [3] and specifically: circulatory, cerebrovascular, ischemic heart diseases and lung cancer mortality for NO_2_. The purpose is to provide up-to-date scientific evidence for the associations under investigation, including an assessment of the certainty of the evidence, and to provide quantitatively pooled effect estimates for each pollutant-outcome combination from meta-analysis based on single exposure results to help inform the selection of concentration-response functions and associated information for relevant health risk assessment.

## Methods

We included general population cohorts that reported on long-term (months to years) exposure to: NO_2_ and all-cause mortality, mortality due to circulatory diseases, ischemic heart diseases (IHD), respiratory diseases (chronic obstructive pulmonary disease (COPD), acute lower respiratory infections (ALRI) and lung cancer; and O_3_ (annual or peak/warm season) and all-cause mortality, mortality due to respiratory diseases, COPD and ALRI (Supplementary Appendix S1).

We searched the PubMed and EMBASE databases for studies published from September 2018 — the last search date that informed the previous WHO AQG review, up to May 2023 (Supplementary Material S3; Appendix S2 Table A1). As the current review includes mortality due to circulatory, cerebrovascular and ischemic heart diseases for NO_2_ not considered previously [3], we re-assessed articles published before September 2018 that were identified but excluded in the previous systematic review [3] as they were not part of the targeted outcomes at the time and we evaluated these studies following our current protocol. An updated search was performed in January 2024 to identify articles published after May 2023 to enrich the discussion of the current report. When multiple publications using the same cohorts were identified, we selected the study reporting on the largest cohort sample size, or as a second criterion the most recent paper. A publication was not included in the meta-analysis if it reported on a specific cohort that was part of greater collaborative project and publication reporting pooled effects between multiple cohorts. We selected the publication that reported the pooled results (and extracted the pooled effect estimate) of the analyzed cohorts and not cohort specific results or cohort specific papers. This decision 1) reduces heterogeneity due to exposure assessment and statistical methods between cohorts and 2) avoids including the same (potentially large administrative) cohort many times in the meta-analysis. Only if the cohort baseline sample size differed by more than 25% or the number of additional years reported in a cohort-specific paper was equal to or greater than the number of overlapping years with those contributing to the pooled analysis, the decision was to include both papers (the pooled analysis and the cohort-specific).

We retrieved information on: publication details; study characteristics; study population; exposure details outcome assessment; exposure unit of measurement; risk estimate as measure of association with 95% confidence interval (CI) with corresponding exposure increment (Supplementary Material S1).

Effect estimates (Hazard Ratios (HR) or Relative Risks (RR) together with 95% CIs) from single pollutant models were extracted from the selected studies and were used to calculate the pooled effect estimates. When multiple estimates were reported, we selected those reported by study authors as the “main” model in the methods section in accordance with the previous reviews [2, 3]. In cases where the effect estimates were presented per increase in parts per billion (ppb), we converted them to μg/m^3^ using standard factors (1.88 for NO_2_ and 1.96 for O_3_) [4]. All effect estimates are reported per 10 μg/m^3^.

We applied a random-effects meta-analysis using the restricted maximum likelihood method for the estimation of between studies variability [3]. Heterogeneity between studies was assessed by calculating 80% prediction intervals (80% PI), the Cochran’s Q test and the I^2^ statistic. Publication bias was assessed by funnel plots, and the Eggers’ test if 10 studies or more contributed to the meta-analysis.

We performed subgroup analysis to investigate heterogeneity by a) WHO regions (European Region; Region of the Americas; Western Pacific Region) and b) Risk of bias assessment per domain in cases the meta-analyses included studies at high risk of bias (High vs. Low/Moderate). We conducted a meta-regression to assess the impact of the mean level of pollutant concentrations on the effect estimates, in case 10 studies or more contributed to the meta-analysis. In case of less than 10 studies, we performed a subgroup analysis to provide pooled effect estimates separately for studies with mean levels above or below the median of the reported levels’ distribution.

All analyses were performed using the “meta” package (version 6.2-0) in the R statistical software, version 4.3.2 (https://www.r-project.org/).

Individual studies, included in the meta-analysis, were evaluated for the risk of bias (RoB), using the tool developed by WHO for the AQG systematic reviews [3, 5]. For each pollutant – outcome pair, we assessed the certainty of evidence using the modified GRADE (Grading of Recommendations, Assessment, Development and Evaluation) [6], adapted by a working group of experts under the supervision of the WHO Secretariat in the context of the update of the AQGs (Supplementary Material S3; Appendix S1).

## Results

### Characteristics of Studies

**FIGURE 1:**
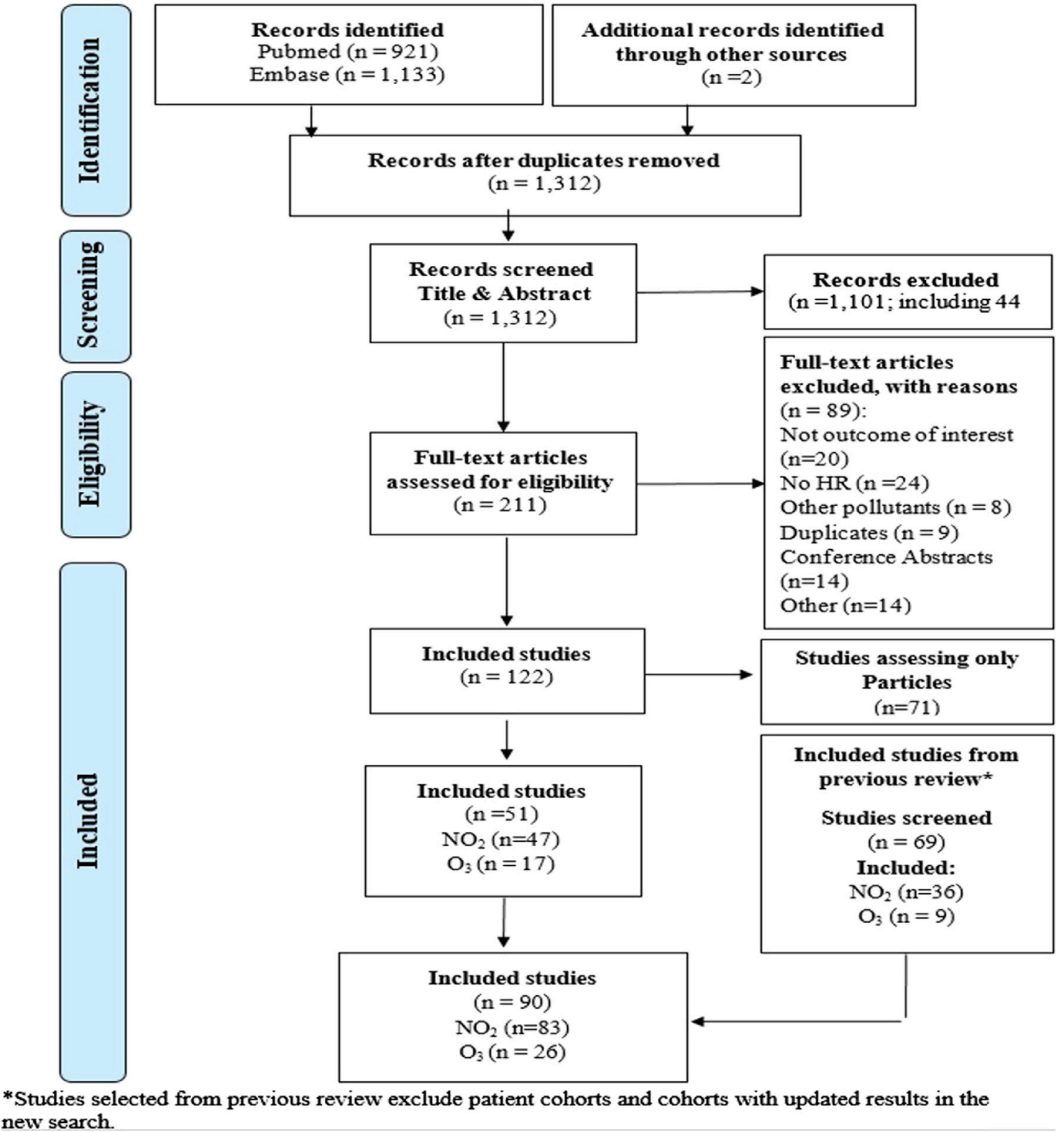
Flowchart for the selection of studies (Global, 2023-2024).

From the search between September 2018 and May 2023, we selected 125 studies, of which 47 reported on long-term exposure to NO_2_ with the outcomes of interest and 17 on O_3_ (annual or peak/warm period) (Figure 1; Supplementary Tables S1, S2). We re-assessed the 69 articles included in the previous meta-analysis (n = 41 for NO_2_ and n = 20 for O_3_) and 23 studies on NO_2_ that assessed other cardiovascular outcomes or lung cancer that were not considered at the time as relevant. Through this step, we further included 36 studies for NO_2_ and 9 studies for O_3._ In total 90 studies, 83 studies for NO_2_ and 26 for O_3_ were included in the current systematic review.

Eighty-three studies reported risk estimates on NO_2_ and mortality outcomes [7–89] (Supplementary Material S3 Table S1). Forty studies were conducted in the European Region, 27 in the Region of the Americas and 16 studies in the Western Pacific. The sample sizes of the selected studies varied from 2,788 to 68,721,015, while several cohorts were included in multiple papers reporting for different follow up periods and sample sizes.

Twenty-six studies assessed the association between exposure to O_3_ and mortality outcomes (Supplementary Material S3 Table S2) [7, 9, 12, 15, 20, 41, 42, 51, 52, 54, 55, 58, 63, 64, 66–68, 72, 73, 77, 90–95]. Ten studies were from the European Region, 13 were from the Region of the Americas and three studies from the Western Pacific Region. The sample sizes of the selected studies varied from 5,652 to 68,721,015.

Detailed RoB assessment for the studies in the meta-analysis is presented in the Supplementary Material S2. For the studies retrieved from Huangfu & Atkinson [3] we adopted the previous evaluation. For the confounding domain, most of the new studies were characterized as low/moderate RoB except for nine [14, 22, 29, 35, 56, 57, 77, 87, 95] that were rated as high; for the exposure assessment domain, two studies were evaluated as high RoB [37, 83]. For the remaining domains, all selected studies were evaluated as low or moderate RoB.

### Meta-Analysis Results

#### Nitrogen Dioxide

**FIGURE 2:**
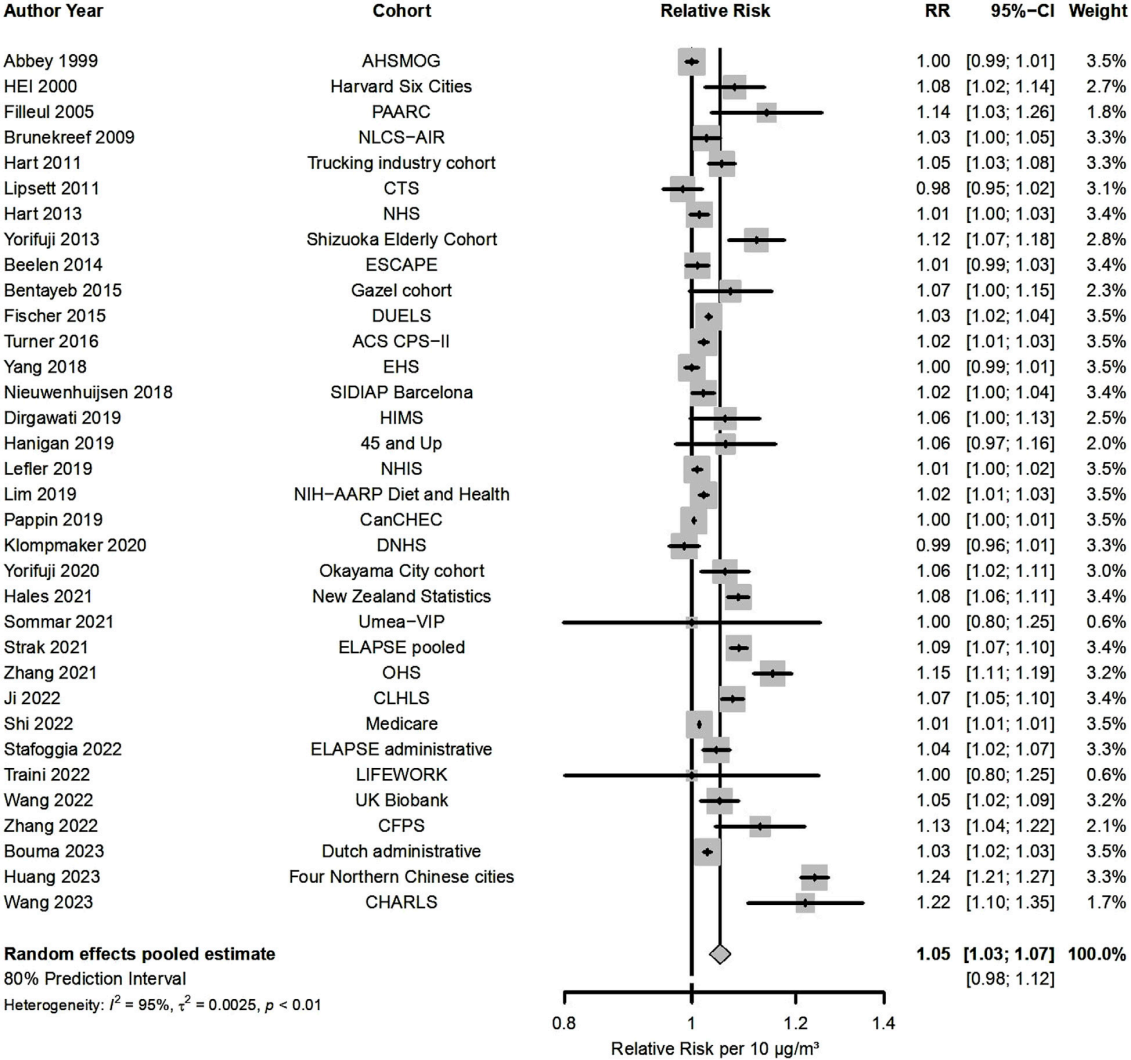
Forest plot for the associations between 10 μg/m^3^ increase in long-term exposure to nitrogen dioxide (NO_2_) and all-cause mortality (Global, 2023-2024).

Fifty-four studies presented results for NO_2_ and all-cause mortality (Figure 2; Table 1), out of which 34 were included in the meta-analysis, indicating that a 10 μg/m^3^ increase was associated with a RR of 1.05 (95% CI: 1.03, 1.07). There was high heterogeneity (80% PI: 0.98, 1.12 and I^2^ = 95%) that contributed to statistically significant funnel plot asymmetry (Supplementary Material S3 Figure S1). RRs were consistently >1 for almost all studies (32 out of 34). Meta-analysis by WHO region (Supplementary Material S3 Figure S2) estimated an RR of 1.03 for the European Region (n = 13), 1.03 for the Region of the Americas (n = 11), while a higher effect estimate was detected for the Western Pacific Region (RR = 1.10, n = 10). Stratification by RoB for confounding resulted in a RR 1.05 for 30 low/moderate RoB vs. 1.02 (Supplementary Material S3 Figure S3) in high RoB studies. Additional subgroup analysis, to further explore heterogeneity in all-cause mortality results, was done according to whether the cohorts were based on administrative databases (n = 7) or collected detailed individual data (n = 27), that indicated statistically significantly higher effects for the detailed cohorts (1.06 vs. 1.02, Supplementary Material S3 Figure S4). Meta-regression on NO_2_ levels did not explain the observed heterogeneity (p-value for levels = 0.103).

**TABLE 1:** Meta-analysis results and characteristics of studies informing the associations between long-term exposure to nitrogen dioxide (NO_2_) (per 10 μg/m^3^ increase) and mortality outcomes (Global, 2023-2024).

	N	Pooled RR (95% CI)	I^2^ (%)	80% prediction interval	Sample size	Age (years)	Median of NO_2_ (min – max)
All-cause	34	1.05 (1.03, 1.07)	95	(0.98, 1.12)	130,487,512	>15	25.0 (7.1—129.9)
Circulatory disease	28	1.05 (1.03, 1.08)	97	(0.97, 1.15)	104,984,429	>15	26.7 (7.1—104)
IHD	20	1.05 (1.03, 1.08)	95	(0.99, 1.12)	96,725,244	>15	26.7 (7.6—104)
Cerebrovascular disease	20	1.08 (0.99, 1.19)	98	(0.82, 1.43)	96,394,524	>25	26.0 (7.6—104)
Respiratory disease	25	1.05 (1.03, 1.07)	90	(0.99, 1.11)	106,631,530	>15	25.0 (7.1—129.9)
COPD	15	1.04 (1.02, 1.06)	62	(0.99, 1.09)	83,982,574	>15	25.7 (20.5—104)
ALRI	9	1.08 (1.04, 1.12)	92	(1.00, 1.16)	52,302,628	>30	25.0 (50.5—104)
Lung cancer	20	1.07 (1.04, 1.10)	92	(0.99. 1.14)	102,667,742	>15	28.9 (7.6—129.9)

^a^ N = number of studies Note: Only Hart et al [35] included population above 15 years old-all other studies include adults ≥18 years old, with the majority ≥30 years old.

Twenty-eight studies estimated a RR 1.05 (95% CI: 1.03, 1.08) (Table 1; Supplementary Material S3 Figure S5). Very high heterogeneity was present (80% PI: 0.97, 1.15 and I^2^ = 97%) with no indication of funnel plot asymmetry (Supplementary Material S3 Figure S6). For the 12 studies in the European Region the RR was 1.02, for the region of the Americas (n = 9) and for the Western Pacific Region (n = 7) the correspondent RRs were 1.06 and 1.10, respectively (Supplementary Material S3 Figure S7). The 26 studies characterized as low/moderate RoB for confounding estimated a higher effect estimate (RR = 1.06) vs. the two high RoB studies (RR = 1.02) (Supplementary Material S3 Figure S8). Heterogeneity was not explained by NO_2_ levels (meta-regression p-value = 0.329).

Thirty-one studies reported results regarding exposure to NO_2_ and IHD mortality from which 20 were included in the meta-analysis estimating, a pooled RR 1.05 (95% CI: 1.03, 1.08) (Table 1). Heterogeneity was very high (Supplementary Material S3 Figure S9). No evidence of small study bias/funnel plot asymmetry was detected (Supplementary Material S3 Figure S10). Stratification by WHO region resulted to a RR of 1.02 for the seven studies that conducted in the European Region, 1.06 for the 9 studies from the Region of the Americas and 1.10 for the four studies from the Western Pacific Region (Supplementary Material S3 Figure S11). All studies were characterized as low/moderate risk of bias, while NO_2_ levels did not explain heterogeneity (meta-regression p-value = 0.467).

From the 31 studies that reported on NO_2_ and cerebrovascular mortality, 20 studies were included in the meta-analysis indicating that a RR of 1.08 (95% CI: 0.99, 1.19) with very high heterogeneity (Table 1; Supplementary Material S3 Figure S12). No evidence of small study bias/funnel plot asymmetry was found (Supplementary Material S3 Figure S13). Meta-analysis stratified by WHO region reported a RR of 1.04 for the European Region (n = 7), 1.01 for the Region of the Americas (n = 7), and 1.23 for the Western Pacific Region (n = 6) (Supplementary Material S3 Figure S14). One study was characterized as high risk of bias for confounding with RR 2.44 (vs. 1.04 for the rest (Supplementary Material S3 Figure S15). NO_2_ levels did not explain heterogeneity (meta-regression p-value = 0.870)

Thirty-five studies reported on NO_2_ and respiratory mortality and included in the meta-analysis estimating a RR 1.05 (95% CI: 1.03, 1.07) (Table 1; Supplementary Material S3 Figure S16) with very high heterogeneity. No evidence of small study bias/funnel plot asymmetry was found (Supplementary Material S3 Figure S17). The RR for the European Region (n = 8) was of 1.05, for the Region of the Americas 1.04 (n = 10) and 1.08 for the Western Pacific Region (n = 7) (Supplementary Material S3 Figure S18). The RR in the 23 low/moderate RoB studies was 1.05 vs. a 1.06 in high risk (n = 2) (Supplementary Material S3 Figure S19). Meta-regression indicated that 33.55% of the heterogeneity was explained by NO_2_ levels (p-value = 0.012) with decreasing effects for increasing levels.

Twenty studies reported effect estimates for NO_2_ and COPD, from which 15 were included in the meta-analysis, estimating RR 1.04 (95% CI: 1.02, 1.06) with substantial heterogeneity (Table 1; Supplementary Material S3 Figure S20). No evidence of small study bias/funnel plot asymmetry was detected (Supplementary Material S3 Figure S21). Stratification by WHO region (Supplementary Material S3 Figure S22) estimated RR 1.06 for the European Region (n = 4), 1.04, for the Region of the Americas (n = 7) and 1.01 for the Western Pacific Region (n = 4). Twelve studies evaluated as low/moderate RoB for confounding had RR 1.03 vs. 1.07 in high RoB ones (Supplementary Material S3 Figure S23). Excluding the high risk of bias studies significantly reduced heterogeneity and the PI did not include unity. NO_2_ levels did not explain heterogeneity (meta-regression p-value = 0.773).

Nine out of 11 studies assessing NO_2_ and ALRI mortality were included in the meta-analysis estimating RR 1.08 (95% CI: 1.04, 1.12) (Table 1; Supplementary Material S3 Figure S24) with very high heterogeneity, but no indication of publication bias (Supplementary Material S3 Figure S25). Only one study was conducted in the European Region (RR = 1.10). The four studies from the Region of the Americas had RR 1.10, and four studies from the Western Pacific Region 1.05 (Supplementary Material S3 Figure S26). All included studies were characterized as low/moderate for RoB. Studies with mean levels of NO_2_ lower than 21 μg/m^3^ (n = 4) estimated RR 1.10 versus 1.04 for those with levels equal or above 21 μg/m^3^ (n = 5) (Supplementary Material S3 Figure S27).

Twenty out of 31 studies identified for the association between NO_2_ and lung cancer mortality were included in the meta-analysis, and estimated RR 1.07 (95% CI: 1.04, 1.10) with high heterogeneity (Table 1; Supplementary Material S3 Figure S28) but no indication of small study bias (Supplementary Material S3 Figure S29). The RR for the European Region (n = 8) was 1.09, 1.02 for the Region of the Americas (n = 7) and 1.13 for the Western Pacific Region (n = 5) (Supplementary Material S3 Figure S30). One study was characterized as high risk of bias for confounding (RR = 1.27), while excluding this study the pooled RR was 1.06 (Supplementary Material S3 Figure S31). Meta-regression on NO_2_ levels indicated that 21.44% of the heterogeneity could be explained (p-value = 0.054), with increasing effects with increasing levels.

We extracted information on the shape of the concentration-response function for each exposure-outcome pair. Most studies used splines (natural or penalized splines) with varying degrees of freedom and compared to the linear model by model fit criteria, such as the model likelihood and BIC. All studies indicated increasing risk with increasing NO_2_ levels for all pairs, while the vast majority indicated linear or supra-linear shapes, i.e., linear shapes with steeper slopes at low pollutant levels (Supplementary Appendix S3).

Eighteen studies adjusted for PM_2.5_ [11, 15, 25, 30, 33, 44, 46, 51, 53, 55, 58, 67, 68, 76, 79, 80, 84, 85]; one for PM_10_ [28]; nine for O_3_ [14, 40, 51, 55, 66–68, 76, 84]; four for black carbon/smoke [22, 30, 68, 79]; two for PM_2.5-10_ [11, 51] and SO_2_ [22, 46], and one for each of SO_4_ [42] and CO [51]. In most of the studies, further adjustment for other pollutants did not affect the effect estimate of interest. Only five studies reported a significant impact on the estimates of interest (Supplementary Appendix S3)

Certainty of evidence assessments for NO_2_ and mortality outcomes are presented in the Supplementary Tables S3–S11. Most associations were assessed as moderate certainty, while COPD and ALRI mortality were assessed as high certainty (pooled RRs 1.04 and 1.08 correspondingly) and cerebrovascular mortality as low (RR = 1.08). Specifically, for all-cause mortality, 32 out of 34 studies estimated RR above unity and although the Egger’s test indicated funnel plot asymmetry the certainty in the evidence was not downgraded, as this was attributed to heterogeneity in the magnitude of the RRs. Heterogeneity was partly explained by regional differences, the high risk of bias studies, and whether the cohorts were based on administrative databases or were cohorts with detailed individual data. For COPD mortality, although there was high heterogeneity as reflected by the large PI, certainty was not downgraded, as when excluding the high risk of bias studies the PI no longer included unity.

#### Ozone

**FIGURE 3:**
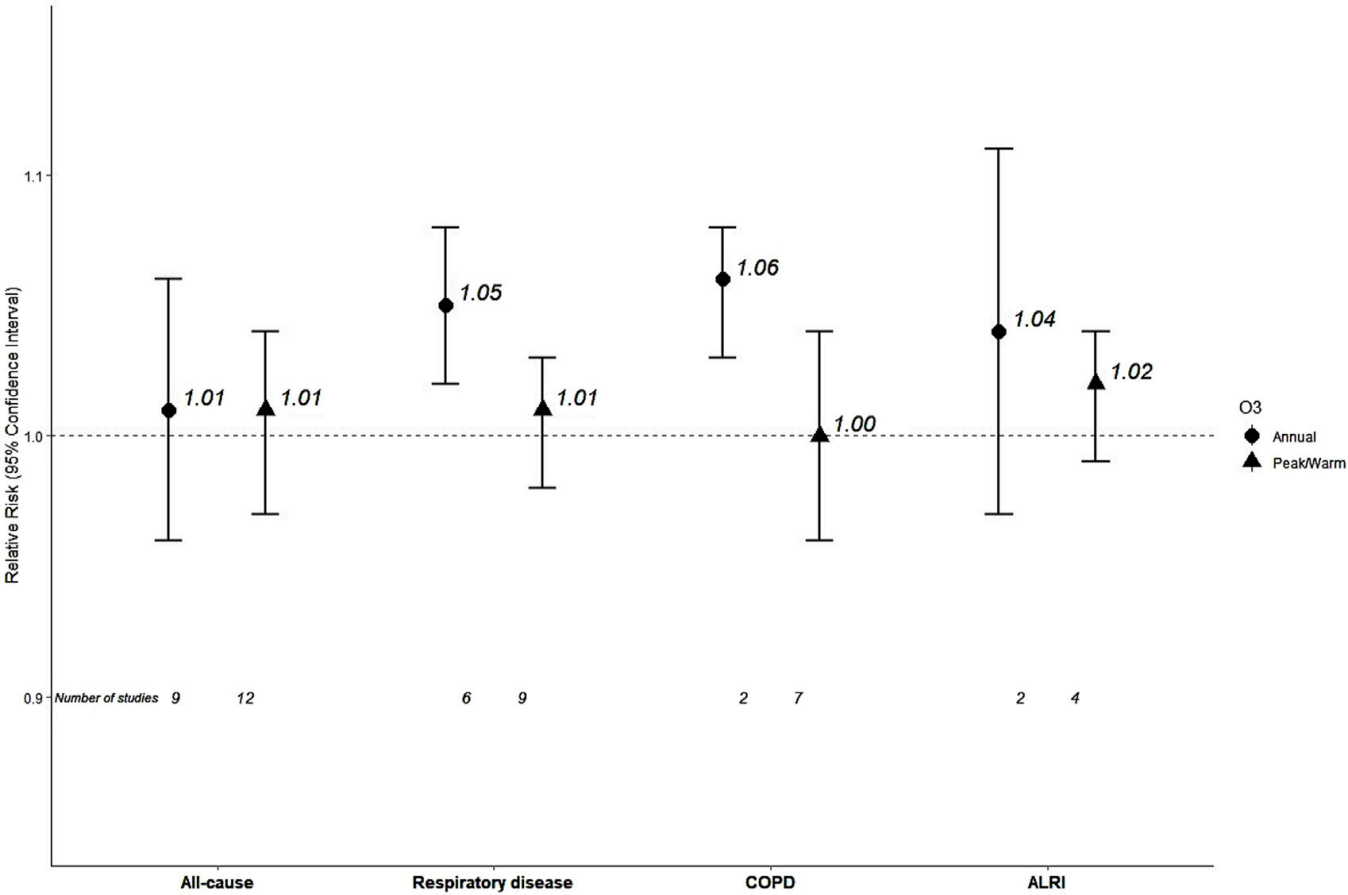
Pooled effect estimates for associations between 10 μg/m^3^ increase in long-term exposure to annual or peak/warm season ozone (O_3_) and mortality outcomes (Global, 2023-2024): COPD, Chronic Obstructive Pulmonary Disease; ALRI, Acute Lower Respiratory Infections.

Nine studies reported on annual O_3_ and all-cause mortality contributing to a pooled RR of 1.01 (95% CI: 0.96, 1.06) (Table 2; Figure 3; Supplementary Material S3 Figure S32). There was very high heterogeneity, while the funnel of indicated no sign of asymmetry (Supplementary Material S3 Figure S33). Meta-analysis stratified by WHO region (Supplementary Material S3 Figure S34) reported an RR of 0.92 for the European Region (n = 2), 1.00 Region of the Americas (n = 5) and a higher effect estimate was detected for the Western Pacific Region (RR = 1.10, n = 2). All studies were low/moderate risk of bias, while heterogeneity was not explained by O_3_ levels (meta-regression p-value = 0.501).

**TABLE 2:** Meta-analysis results and characteristics of studies informing the associations between long-term exposure to ozone (O_3_) (per 10 μg/m^3^ increase) and mortality outcomes (Global, 2023-2024).

A) Annual O_3_							
	N	RR (95% CI)	I^2^ (%)	80% prediction interval	Sample size	Age (years)	Median of O_3_ (min – max)
All-cause	9	1.01 (0.96, 1.06)	92	(0.91, 1.11)	1,622,831	>30	60.6 (42.3–94.3)
Respiratory disease	6	1.05 (1.02,1.08)	65	(1.00, 1.09)	1,544,274	>30	70.4 (50.8–94.3)
COPD	2	1.06 (1.03, 1.08)	25	-	1,217,826	>30	74.87–76.44*
ALRI	2	1.04 (0.97, 1.11)	80	-	1,217,826	>30	74.87–76.44*

B) Peak/warm season O3							
	N	RR (95% CI)	I^2^ (%)	80% Prediction Interval	Sample size	Age (years)	Median of O_3_ (min – max)
All-cause	12	1.01 (0.97, 1.04)	98	(0.92, 1.10)	107,896,036	>18	90.6 (74.9–106.6)
Respiratory disease	9	1.01 (0.98, 1.03)	92	(0.95, 1.06)	57,107,136	>25	85 (74.9–109.8)
COPD	7	1.00 (0.96, 1.04)	95	(0.92, 1.09)	56,668,296	>25	85 (74.9–109.8)
ALRI	4	1.02 (0.99, 1.04)	88	(0.97, 1.07)	23,702,383	>30	87.9 (74.9–109.8)

N = number of studies; min: minimum; max: maximum.

Seven studies reported results for O_3_ and respiratory mortality from which six contributed in the meta-analysis that estimated RR of 1.05 (95% CI: 1.02, 1.08) for mortality from respiratory diseases (Table 2; Supplementary Material S3 Figure S35). Heterogeneity was substantial and no evidence of small study bias/funnel plot asymmetry was found (Supplementary Material S3 Figure S36). One study was in European Region (RR = 0.79), four studies in the Region of the Americas with RR 1.04 and one with RR 1.20 (95% CI: 1.02, 1.41) in the Western Pacific Region (Supplementary Material S3 Figure S37). All studies were characterized as low/moderate risk of bias. Studies with levels below 69 μg/m^3^ (n = 3) estimated an RR of 1.10 versus 1.04 for those equal or above 69 μg/m^3^ (n = 3) with no statistically significant difference (p = 0.43, Supplementary Material S3 Figure S38).

The polled estimated of two studies was 1.06 (95% CI: 1.03, 1.08) for COPD mortality, with low heterogeneity (I^2^ = 25%) (Table 2; Supplementary Figure S39).

The polled estimated of two studies was 1.04 (95% CI: 0.97, 1.11) for ALRI mortality, with substantial heterogeneity (I^2^ = 80%) (Table 2; Supplementary Material S3 Figure S40).

Twelve out of 23 studies reporting on peak/warm season O_3_ and all-cause mortality, estimated a pooled 1.01 (95% CI: 0.97, 1.04) for mortality from all-causes (Table 2; Supplementary Material S3 Figure S41), with very high heterogeneity. There were no signs of publication bias/funnel plot asymmetry (Supplementary Material S3 Figure S42). Meta-analysis stratified by WHO region (Supplementary Material S3 Figure S43) reported an RR of 0.93 for the European Region (n = 3), 1.01 (for the Region of the Americas (n = 6), and a higher effect estimate for the Western Pacific Region (RR = 1.07, n = 3). Stratification by RoB for confounding resulted in an RR of 1.00 for low/moderate RoB studies (n = 11) vs. one high RoB study with an RR of 1.07 (Supplementary Material S3 Figure S44). Meta-regression on 11 studies reporting O_3_ levels indicated that a statistically significant 25.30% of the observed heterogeneity was explained by peak/warm O_3_ levels (p = 0.041), with increased risk associated with increased levels.

Nine from the 15 studies that reported on peak/warm O_3_ and respiratory mortality, estimated an RR of 1.01 (95% CI: 0.98, 1.03) (Table 2; (Supplementary Material S3 Figure S45) with very high heterogeneity, but no evidence of funnel plot asymmetry (Supplementary Material S3 Figure S46). The meta-analysis by WHO region (Supplementary Material S3 Figure S47) estimated an RR of 0.93 for the European Region (n = 2), 1.02 for the Region of the Americas (n = 6) and 1.13 for the Western Pacific Region (n = 1). Stratification by RoB for confounding estimated an RR of 1.00 for the 8 low/moderate RoB studies vs. 1.02 for the high risk of bias study (n = 1) (Supplementary Material S3 Figure S48). Studies with mean levels of peak/warm O_3_ lower than 85 μg/m^3^ (n = 4) estimated an RR of 1.02 versus 0.99 for those with levels equal to or above 85 μg/m^3^ (n = 5) (p for difference = 0.240, Supplementary Material S3 Figure S49).

Seven out of eight studies that reported effect estimates for peak/warm O_3_ and COPD mortality estimated an RR of 1.00 (95% CI: 0.96, 1.04) with high very heterogeneity (Table 2, Supplementary Material S3 Figure S50) and no evidence of funnel plot asymmetry (Supplementary Material S3 Figure S51). The RR for the European Region (n = 2) was 0.91, while it was 1.02 for the Region of the Americas (n = 4, Supplementary Material S3 Figure S52). All selected studies were characterized as low/moderate RoB. Studies with mean levels lower than 85 μg/m^3^ (n = 3) estimated an RR of 1.02 versus 0.97 (n = 4) (p for difference = 0.36, Supplementary Material S3 Figure S53).

Four studies associated a 10 μg/m^3^ increase in peak/warm O_3_ with an RR of 1.02 (95% CI: 0.99, 1.04) for ALRI mortality with high heterogeneity (Table 2; Supplementary Material S3 Figure S54), and no indication of publication bias (Supplementary Material S3 Figure S55). Only one study was in the European Region (RR = 0.93) and the rest in the Region of the Americas with an RR of 1.02 (Supplementary Material S3 Figure S56). All included studies were low/moderate for the two studies with mean levels of lower than 88 μg/m^3^ estimated an RR of 1.04 versus 1.01 for the two with levels equal or above 88 μg/m^3^ (p for difference = 0.04, Supplementary Material S3 Figure S57).

Most of the studies assessing O_3_ effects applied splines (natural or penalized splines) and assessed linearity by model fit criteria, with the vast majority reporting no deviations from linearity (Supplementary Appendix S3). Among the studies reporting multi-pollutant models, the vast majority adjusted for particles and this adjustment did not considerably influence the estimates (Supplementary Appendix S3).

Certainty of the evidence assessments for O_3_ are summarized in Supplementary Tables S12–S18. For annual O_3_ the certainty was low for all-cause (RR = 1.01) and high for respiratory mortality (RR = 1.05), while for peak/warm O_3_ the certainty of evidence was low for all the mortality outcomes (all-cause RR = 1.01; respiratory RR = 1.01; COPD RR = 1.00; ALRI: RR = 1.02).

### New Studies Published After Final Search

The updated search identified 438 articles published between May 2023 and January 2024. We assessed 36 following the initial screening and 15 fulfilled our eligibility criteria. Of these, seven [96–102], reported on NO_2_ and O_3_: three from the UK Biobank [97, 98, 101], out of which Wang et al. [101] reported larger RRs (1.12, 95%CI: 1.10, 1.14 for all-cause; 1.14, 95%CI: 1.10, 1.18 for circulatory) than those included from the UK Biobank in the meta-analysis (1.05, 95%CI: 1.01, 1.08 and 1.04, 95%CI: 0.97, 1.11 respectively). One study reported from CanCHEC [100] on NO_2_ and circulatory mortality but using a smaller sample than the included study in our analysis [20]. Results from two new European cohorts were published: 1) the RHINE III cohort [102], with 9,135 participants, reported estimates for all-cause mortality and NO_2_ (1.03, 95%CI: 0.84, 1.26 per 10 μg/m^3^) and annual O_3_ (0.91, 95%CI: 0.68, 1.21 per 10 μg/m^3^); and 2) an administrative Scottish cohort [96] reported RRs for NO_2_ and mortality outcomes, all of which were larger than the pooled RRs in our analysis but within the 80%PI, except for respiratory morality. Finally, we identified one paper using the China Family Panel with results for peak O_3_ and all-cause mortality [99] reporting an RR of 1.05 (95%CI: 1.01, 1.09).

## Discussion

We updated the evidence base that informed the WHO global air quality guidelines for the associations between long-term exposure to NO_2_ and O_3_ and mortality outcomes with new studies published between 2018 and 2023, including large administrative cohorts and new reports from the Western Pacific Region. We further assessed findings on circulatory, cerebrovascular, ischemic heart diseases and lung cancer mortality for NO_2_ that were not considered in the previous review [3].The publication of findings from large consortia in different parts of the world and the analysis during the recent years of large administrative databases including country-wide populations highlight the need for an update of the associations. Our meta-analysis identified an increasing number of studies as compared to previous reviews and estimated for NO_2_ statistically significant RRs between 1.04 and 1.08 per 10 μg/m^3^ for all cause, circulatory, IHD, respiratory, COPD, ALRI and lung cancer mortality. The RR with cerebrovascular mortality was 1.08 but did not achieve the nominal level of significance. Using the adapted GRADE tool, we assessed the certainty in the evidence as being moderate in all cases except for COPD and ALRI mortality (rated as high) and for cerebrovascular mortality (rated as low).

Evidence increase also is associated with increased effect estimates compared with those reported in the review by Huangfu & Atkinson [3]: the RRs for all-cause, respiratory, COPD and ALRI mortality were 1.05, 1.05, 1.04 and 1.08 vs. 1.02, 1.03, 1.03 and 1.06 in the previous review. The difference between the two reviews is that the current update focused on general population cohorts and did not include evidence on patient populations. In the previous review, six patient cohorts were included in the NO_2_ all-cause mortality association (out of the 24 total studies), the exclusion of which did not alter the pooled relative risk (1.02) but does highlight the increase in the number of studies from 18 general population ones in the previous review to 34 in ours. The certainty in the evidence for an association did not change in these outcomes, except for COPD mortality that was increased from moderate to high. In our review we included cardiovascular and lung cancer mortality outcomes that were assessed in more than 20 studies and all associations were rated as of moderate certainty, except for cerebrovascular that was low.

A recent review on traffic related air pollution assessed NO_2_, although studies in the review and the meta-analysis were selected after scrutiny for the pollution’s source that resulted in reports highly specific on traffic [103]. The evidence base was less than a third of ours but the pooled RRs are similar as they reported RR 1.04 for all-cause, circulatory and lung cancer mortality, 1.05 for respiratory and IHD and 1.03 for COPD. The RR for stroke (the main contributor to cerebrovascular mortality as assessed in our review) was 1.01 that is much lower than 1.08 in the current report. Researchers in the traffic review adapted a modified GRADE tool for the certainty in the quality of the body of evidence using the Office of Health Assessment and Translation (OHAT) method as a guide [104]. All mortality outcomes were rated as high certainty in the association with NO_2_, except for stroke that was moderate and COPD that was as low [105]. Our assessment in the certainty for an association is more conservative, except for COPD, but we have an evidence base of 15 studies compared with 3 in the traffic review and the tool applied is different.

The evidence base has also increased for the associations with annual or peak/warm season O_3_ compared to Huangfu & Atkinson [3], but still the studies are few compared to NO_2_ related research. Although we estimated pooled RRs above unity for either annual or peak/warm O_3_ and all-cause, respiratory, COPD and ALRI mortality, only associations between annual O_3_ and respiratory or COPD mortality were statically significant. All associations were rated as low certainty in the evidence for an association except for annual O_3_ with respiratory mortality that was rated as high. This is an upgrade since Huangfu & Atkinson [3], which that reported 0.99 (95%CI: 0.89, 1.11) and low certainty for respiratory mortality. On the other hand, we rated the associations between peak/warm season O_3_ with all-cause mortality as low certainty, while they were previously rated as moderate. Although both reviews estimated the same RR of 1.01, the increasing number of studies in our review (i.e., 12 vs. 7 before) resulted in an increase in the width of the 95%CI of the pooled RR and inclusion of 1.

There was high heterogeneity in all results, except for NO_2_ and ALRI and annual O_3_ and respiratory mortality. The precise estimates from large administrative cohorts contributed to high I^2^ values, but not necessarily to the wide PIs that estimate the expected range of true effects in similar studies. Heterogeneity is expected considering that the studies were conducted in varying locations across the globe although most came from western Europe, the United States, and Canada. No studies were identified in the African Region, the Southeast Asian Region or Latin America and the Caribbean fitting our eligibility criteria. Analysis by WHO regions and exclusion of high risk of bias studies (in the confounding domain) reduced heterogeneity. Heterogeneity between WHO regions was mostly associated with the magnitude of the RR, giving comparable results between the European and the Americas’ Regions and higher effect estimates in the Western Pacific Region for all associations except for NO_2_ with COPD and ALRI mortality. For peak/warm season O_3_ the European Region’s results differed in direction from those in other areas, as they were below unity for all outcomes. Nevertheless, these European results were mainly based on the ELAPSE [15, 55, 67, 68] study that has attributed these findings to the small exposure range for O_3_ potentially driven by the European-wide exposure assessment. The levels of the pollutants explained heterogeneity in the association between NO_2_ and respiratory and ALRI mortality, with decreasing effects with increasing levels (partly attributed to supra-linear shapes or outliers in the meta-regression) and in the opposite direction with lung cancer mortality. Increasing peak/warm season O_3_ levels were associated with higher effects for all-cause but reduced effects for ALRI mortality (the latter in accordance with NO_2_). Finally, we do not expect that the age at recruitment to have contributed to the observed heterogeneity. With the exception of one study, all assessed studies involved adult populations (≥18 years old), and the majority focused on populations ≥30 years old.

At least half the studies in any of the exposure-outcome pairs investigated the shape of the concentration-response function. There was a consensus of no deviation from linearity, especially within the most commonly observed range of the exposure distribution for any of the associations. This implies increasing effects with increasing levels in all pairs, except for the case of peak/warm season O_3_ and mortality in Europe as estimated within the ELAPSE project that assessed a downward trend with increasing levels. For pairs that indicated increasing effects with increasing levels and no deviation from linearity most pointed towards steeper slopes at lower levels. This is of particular interest considering that for all pairs the median levels for NO_2_ levels reported in the various cohorts exceeded the annual WHO AQG level [1].

Among the strengths of the study is the long time period covering our search, the large number of studies especially for new outcomes and new regions of the world, the investigation of heterogeneity and the assessment of linearity. We closely followed the rationale of the previous reviews in assessing the new studies and the updated evidence. The increase in the related publications, including large collaborative projects that cover large areas and/or large administrative data sets, highlights the importance of updating relevant reviews as there is continuously emerging evidence for new health outcomes and an increase in the information from areas outside western Europe, the United States and Canada.

Despite the increasing evidence base, however, the investigation of heterogeneity sources may still be limited for some outcomes, as was the case for cause-specific mortality, or within regions, where the number of studies decreased. Heterogeneity may partly be attributed to varying exposure assessment methods used between cohorts including the use of different spatial coverage, grid resolution, and temporality.

A potential limitation in all systematic reviews (previous and current) is the choice of effect estimates from single exposure models. This is largely guided by the insufficient statistical information reported in multi-pollutant models, which complicates the ability to properly pool effects accounting for the variance-covariance structure in meta-analysis and reflecting the varying correlations across different areas. This raises concerns about the independence of the effects of the assessed pollutants. To address this concern, we descriptively followed the rationale of the authors that did report on the associations from multi-exposures models on the change in the magnitude of the associations of interest from single to multi pollutant models. It is of interest to note that if reviews were to account only for results from multi-pollutant models, this would decrease the evidence base as not all studies applied such models and the co-pollutants varied greatly (with most adjusting for PM_2.5_).

The above complexities, when considering various exposure-outcome pairs that may additionally present different degrees of heterogeneity, especially under a health risk assessment or a burden of disease framework, have been recognized in relevant endeavors undertaken by the WHO and the Global Burden of Disease study [105, 106]. For the purpose of this report heterogeneity only weakened certainty if it could not be partly attributed to the limited set of factors considered and there was diversity on the direction of extracted effect estimates.

Limitations are also related to specific items in the Risk of Bias and the adapted GRADE tool [2, 3]. Most notably the Rob tool includes the assessment of exposure contrast that is rated as low if between-subject’ variability is larger than within-subject variability. This kind of information is not reported in published papers and could not be assessed. A smaller limitation in the RoB tool is that, depending on the design of the studies and considering the growing literature using country-wide assessments, the list of proposed confounders may be limited in identifying high risk of bias studies, as it is based on known risk factors but does not account for spatial confounding that may be particularly important in air pollution research as country-wide investigations should also adjust for potential regional differences. Regarding the use of the modified GRADE, the risks in air pollution epidemiology are considerably small compared to other risk factors and may be affected disproportionally by several factors in terms of observed heterogeneity. Similar concerns were raised by the traffic review panel [103, 107]. They noted that heterogeneity in the magnitude of effect estimates should generally not weaken the certainty in the evidence, as applied in the GRADE domain on inconsistency. They proposed that rather certainty should be strengthened, if previous evidence greatly points to the same direction of effects and called for a modification of GRADE-type frameworks to align better with features of environmental health questions and related studies. Initially we have opted to account for this rationale if part of the heterogeneity could be explained by our analysis and all evidence was consistent in direction, but this was not the case in any of the examined associations.

Although all aforementioned limitations do not limit the certainty in the associations, they warrant further development. Last but not least and considering the exponentially growing literature in air pollution epidemiology, it is necessary to acknowledge that publication time does not reflect the most recent research findings, as research is bound to delays due to data availability but also necessary time for analysis and interpretation. Nevertheless, considering the support for mostly linear or supra-linear associations in our review, potentially decreasing levels of pollution in recent years do not undermine our conclusions.

Our review and meta-analysis revealed a large number of new studies investigating long-term exposure to gases and mortality, including large consortia covering greater areas, administrative datasets that in many cases represent country-wide populations and studies from the Western Pacific Region. Our results reinforce the scientific community’s call [108] for the implementation of emission reduction policies aimed at meeting the WHO AQGs. The growing evidence and the investigation of new health outcomes highlight the importance of updating reviews of air pollution epidemiology to strengthen and expand the certainty in air pollution health effects and provide updated estimates to feed into relevant health risk assessments.
