# Supplementary material for: Long-Term Exposure to Nitrogen Dioxide and Ozone and Mortality: Update of the WHO Air Quality Guidelines Systematic Review and Meta-Analysis
Source: Int J Public Health. 2024 Oct 18;69:1607676. doi: 10.3389/ijph.2024.1607676 (PMC11527649; doi:10.3389/ijph.2024.1607676)
Supplement: Supplementary file 1 [file DataSheet3.pdf]

Journal Title: International Journal of Public Health

Article Title: Long-term exposure to nitrogen dioxide and ozone and mortality: Update of the WHO air quality guidelines systematic review and meta-analysis.

Table S1: Characteristics of studies for **Nitrogen Dioxide** (Global, 2023-2024)**.**

ACM: All-cause mortality; CIRC: Circulatory disease mortality; CBV: Cerebrovascular mortality; IHD: Ischemic Heart Disease mortality; RESP: respiratory mortality; COPD: Chronic Obstructive Pulmonary diseases mortality; ALRI: Acute Lower Respiratory Infections mortality; LC: Lung Cancer mortality.

A) For **all-cause and respiratory** mortality identified in the updated systematic review **September 2018 – May 2023**; for **circulatory and lung cancer** mortality in the systematic review **all years up to May 2023**.

Author year	Cohort	Location	Study Period	Sample size	Sex	Age	Outcome
Abbey 1999	AHSMOG	The United States	1977-1992	**5,652**	FM	27-95	**LC**
Hoek 2002	NLCS-AIR	Netherlands (Kingdom of the)	1987-1996	2,788	FM	55-69	LC
Filleul 2005	PAARC	France	1974-2000	14,284	FM	25-59	**LC**
Chen 2005	AHSMOG	California, the United States	1976-2000	3,239	FM	>=25	**IHD**
Gehring 2006	German cohort	Germany	1985-2003	4,752	F	50-59	CIRC, LC
Naess 2007	Oslo Cohort	Norway	1992-1998	143,842	FM	51-90	**CIRC, LC**
Schikowski 2007	SALIA	Germany	1985-2003	4,750	F	54.5	CIRC
Brunekreef 2009	NLCS-AIR	Netherlands (Kingdom of the)	1987-1996	120,227	FM	55-69	**LC, CIRC**
Beelen 2009	NLCS	Netherlands (Kingdom of the)	1987-1996	117,528	FM	55-69	**IHD, CBV**
Yorifuji 2010	Shizuoka Elderly Cohort	Japan	1999-2006	12,209	FM	65-84	CIRC, IHD, CBV, LC
Katanoda 2011	Three-prefecture Cohort Study	Japan	1983-2005	63,520	FM	>=40	**LC**
Lipsett 2011	CTS	The United States	1997-2005	12,336	F	>=30	**CIRC, IHD, CBV, LC**
Hart 2011	Trucking industry cohort	The United States	1985-2000	53,814	M	15.3-84.9	**CIRC, IHD, LC**
Zhang 2011	Shenyang	China	1998-2009	9,941	FM	35–103	CIRC, **CBV**
Gan 2011	Metro Vancouver	Vancouver, Canada	1999-2002	452,735	FM	45–85	**IHD**
Raaschou-Nielsen 2012	DCH	Denmark	1993-2009	52,061	FM	50-64	CIRC, IHD, CBV
Andersen 2012	DCH	Denmark	1993-2006	52,215	FM	50-65	CBV
Cesaroni 2013	RoLS	Italy	2001-2010	1,265,058	FM	>=30	CIRC, IHD, CBV, LC
Jerrett 2013	ACS CPS-II	The United States	1982-2000	73,711	FM	57.4(10.6)	CIRC, IHD, LC
Heinrich 2013	German cohort	Germany	1985-2008	4,752	F	50-59	**LC**
Yorifuji 2013	Shizuoka Elderly Cohort	Japan	1999-2009	13,412	FM	65-84	**CIRC, IHD, CBV, LC**

Chen 2013	Ontario Tax File cohort	Canada	1982-2004	205,440	FM	35-85	**CIRC, IHD, CBV**
Beelen 2014	ESCAPE	Europe	1985-2007	367,383	FM	>=41	**CIRC, IHD, CBV**
Fischer 2015	DUELS	Netherlands (Kingdom of the)	2004-2011	7,218,363	FM	>=30	**CIRC, LC**
Bentayeb 2015	Gazel cohort	France	1989-2013	20,327	FM	35-50	**CIRC**
Crouse 2015	CanCHEC	Canada	1991-2006	2,521,525	FM	25-89	**CIRC, IHD, CBV, LC**
Crouse 2015	CanCHEC	Canada	1991-2006	735,590	FM	25-89	CIRC, IHD, CBV
Tseng 2015	Civil servants and teachers	Taiwan, China	1989-2008	43,227	FM		**CIRC**
Chen 2016	Four Northern Chinese cities	China	1998-2009	39,054	FM	23-89	LC
Turner 2016	ACS CPS-II	The United States	1982-2004	669,046	FM	>=30	**CIRC, IHD, CBV, LC**
Weichenthal 2017	CanCHEC	Canada	2001-2011	2,448,500	FM	25-89	CIRC
Dehbi 2017	NSHD- SABRE	The United Kingdom	1989-2015	7,529	FM	48.45*	**CIRC**
Nieuwenhuijsen 2018	SIDIAP Barcelona	Spain	2010-2014	792,649	FM	>=18	**ACM**
Yang 2018	EHS	Hong Kong Special Administrative Region, China	1998-2011	61,386	FM	>=65	ACM, **CIRC**, RESP, COPD , ALRI, **IHD, CBV**
Dirgawati 2019	HIMS	Australia	1996-2012	11,627	M	>=65	**ACM, CBV**
Hanigan 2019	45 and Up	Australia	2005-2015	75,145	FM	45-79	**ACM**
Héritier 2019	SNC	Switzerland	2000-2008	4,404,046	FM	>=30	IHD
Hvidtfeldt 2019	DCH	Denmark	1993-2015	49,564	FM	50-64	ACM, CIRC, RESP
Lefler 2019	NHIS	The United States	1987-2015	635,539	FM	18-84	**ACM**
Lim 2019	NIH-AARP Diet and Health	The United States	1995-2011	548,780	FM	50–71	**ACM**, CIRC, **RESP, COPD ALRI**, IHD, CBV**, LC,**
Lim 2019	NIH-AARP Diet and Health	The United States	1995-2011	548,845	FM	50–71	**CIRC**, **IHD**, **CBV**
Pappin 2019	CanCHEC	Canada	1991-2016	8,500,000	FM	24-89	**ACM**
Klompmaker 2020	DNHS	Netherlands (Kingdom of the)	2013-2017	339,633	FM	>=30	**ACM, CIRC, RESP, COPD IHD, CBV, LC**
So 2020	DNC	Denmark	1993-2013	24,541	F	>=44	ACM, CIRC, RESP, COPD IHD, CBV
Yorifuji 2020	Okayama City cohort	Japan	2006-2016	73,970	FM	>=40	**ACM, CIRC, RESP, COPD ALRI, IHD, CBV, LC**
Eum 2020	Medicare	The United States	2000-2008	14.1 m	FM	65-120	ACM, CIRC, RESP, COPD ALRI, IHD, CBV, LC

Brunekreef 2021	ELAPSE administrative	Europe	2000-2017	28,153,138	FM	>=30	**COPD , IHD, CBV**
Gariazzo 2021	RoLS	Italy	2001-2015	482,259	FM	>=30	ACM, CIRC, RESP
Hales 2021	New Zealand Statistics	New Zealand	2013-2016	2,227,332	FM	>=30	**ACM, CIRC, RESP, IHD, CBV, LC**
Kim 2021	KNHANES-M	Republic of Korea	2007-2016	18,220	FM	>=30	**CIRC, RESP**
Klompmaker 2021	Dutch administrative	Netherlands (Kingdom of the)	2008-2012	10,532,360	FM	>=29	ACM, CIRC, RESP, COPD IHD, CBV, LC
Klompmaker 2021	Dutch administrative	Netherlands (Kingdom of the)	2013-2018	10,481,566	FM	>=30	ACM, CIRC, RESP, COPD **IHD, CBV**, LC
Qian 2021	Medicare	The United Kingdom	2000-2016	13,590,387	FM	>=65	ACM
Samoli 2021	ELAPSE pooled	Europe	1992-2005	325,367	FM	48.7	ACM
Sommar 2021	Umea-VIP	Sweden	1990-2014	42,580	FM	40 **	**ACM, CIRC, RESP**
Strak 2021	ELAPSE pooled	Europe	1990 -2015	325,367	FM	48.7	**ACM, CIRC, RESP, COPD IHD, CBV**
Zhang 2021	OHS	Canada	2009-2017	88,615	FM	>=30	**ACM, CIRC, RESP**
Zhang 2021	ONPHEC	Ontario, Canada	2001-2016	834,445	FM	40-85	**CIRC**
Zhang 2021	ONPHEC	Ontario, Canada	2001-2016	834,445	FM	40-85	**RESP, COPD , ALRI**
Zhao 2021	CanCHEC	Canada	2001-2016	3,209,100	FM	>=25	CBV
Bauwelinck 2022	Belgian administrative	Belgium	2001–2011	5,474,470	FM	>=30	ACM, CIRC, RESP, LC
Eum 2022	Medicare	The United States	2001-2008	49,712,702	FM	>=65	ACM, **CIRC, RESP, COPD ALRI, IHD, CBV, LC**
Ji 2022	CLHLS	China	2008-2018	11,835	FM	>=65	**ACM**
Liu 2022	ELAPSE pooled	Europe	1990 -2015	325,367	FM	48.7	**ALRI**
Shi 2022	Medicare	The United States	2001-2017	68,721,015	FM	>=65	**ACM**
So 2022	Danish administrative	Denmark	2000-2017	3,083,227	FM	>=30	ACM, CIRC, RESP, COPD ALRI, IHD, CBV, LC,
Stafoggia 2022	ELAPSE administrative	Europe	2000-2017	28,153,138	FM	>=30	**ACM, CIRC, RESP, LC**
Tian 2022	UK Biobank	England, Wales and Scotland, the United Kingdom	2006-2010	318,752	FM	40-69	ACM
Traini 2022	LIFEWORK	Netherlands (Kingdom of the)	2013-2017	86,882	FM	>=18	**ACM**
Wang 2022	UK Biobank	England, Wales and Scotland, the United Kingdom	2006-2018	386,937	FM	40-69	**ACM, CIRC, IHD, CBV**

Zhang 2022	CFPS	China	2010-2018	30,843	FM	45.6	**ACM**
Bouma 2023	Dutch administrative	Netherlands (Kingdom of the)	2013-2019	10,735,734	FM	>=30	**ACM, CIRC, RESP, LC**
Kulick 2023	WHI	The United States	1993-2010	155410	F	50-79	**CBV**
Vienneau 2023	SNC	Switzerland	2000-2014	4,188,175	FM	>=30	ACM, CIRC, RESP
Wang 2023	UK Biobank	England, Wales and Scotland, the United Kingdom	2006-2019	265,506	FM	37–73	ACM
Wang 2023	CHARLS	China	2011-2018	15,440	FM	>=45	**ACM**
Wu 2023	UK Biobank	England, Wales and Scotland, the United Kingdom	2006-2021	162,334	FM	54	ACM
Zou 2023	UK Biobank	England, Wales and Scotland, the United Kingdom	2006-2021	372,530	FM	37-73	ACM
Huang 2023	Four Northern Chinese cities	China	1998-2019	39,054	FM	43.5 *	**ACM, CIRC, RESP, LC**

**mean value; ** median value, Bold pairs selected to be included in the meta-analysis*

B) For **all-cause and respiratory** mortality identified in the Huangfu & Atkinson 2020 meta-analysis for **search up to September 2018** and included in the current update.

**Author year**	**Cohort**	**Location**	**Study Period**	**Sample size**	**Sex**	**Age**	**Outcome**
Abbey, 1999	AHSMOG	The United States	1977-1992	5,652	FM	27-95	**ACM, RESP**
HEI, 2000	ACS CPS-II	The United States	1980-1989	552,138	FM	>=30	**ACM**
Filleul, 2005	PAARC	France	1974-2000	14,284	FM	25-59	**ACM**
Naess, 2007	Oslo Cohort	Norway	1992-1998	143,842	FM	51-90	**COPD**
Brunekreef, 2009	NLCS-AIR	Netherlands (Kingdom of the)	1987-1996	120,227	FM	55-69	**ACM, RESP**
Katanoda, 2011	Three-prefecture Cohort Study	Japan	1983-2005	63,520	FM	>=40	**RESP, COPD, ALRI**
Lipsett, 2011	CTS	The United States	1997-2005	12,336	F	>=30	**ACM, RESP**
Hart, 2011	Trucking industry cohort	The United States	1985-2000	53,814	M	15.3-84.9	**ACM, RESP, COPD**

Cesaroni, 2013	RoLS	Italy	2001-2010	1,265,058	FM	>=30	ACM, RESP
Hart, 2013	NHS	The United States	1990-2008	84,562	F	30-55	**ACM**
Gan, 2013	Vancouver cohort	Canada	1999-2002	467,994	FM	45-85	**COPD**
Yorifuji, 2013	Shizuoka Elderly Cohort	Japan	1999-2009	13,412	FM	65-84	**ACM, RESP, COPD, ALRI**
Beelen, 2014	ESCAPE	Europe	1985-2005	367,251	FM	All	**ACM**
Dimakopoulou, 2014	ESCAPE	Europe	1985-2005	307,553	FM	All	**RESP**
Fischer, 2015	DUELS	Netherlands (Kingdom of the)	2004-2011	7,218,363	FM	>=30	**ACM, RESP**
Bentayeb, 2015	Gazel cohort	France	1989-2013	20,327	FM	35-50	**ACM**
Crouse, 2015	CanCHEC	Canada	1991-2006	2,521,525	FM	25-89	ACM, **RESP, COPD**
Chen, 2016	Four Northern Chinese cities	China	1998-2009	39,054	FM	23-89	ACM
Turner, 2016	ACS CPS-II	The United States	1982-2004	669,046	FM	>=30	**ACM, RESP, COPD, ALRI**
Weichenthal, 2017	CanCHEC	Canada	2001-2011	2,448,500	FM	25-89	ACM, **RESP**
Yang, 2018	EHS	Hong Kong Special Administrative Region, China	1998-2011	61,386	FM	>=65	**ACM, RESP, COPD, ALRI**

Bold pairs selected to be included in the meta-analysis

Table S2: Characteristics of studies **for Ozone and all-cause and respiratory mortality** (Global, 2023-2024).

ACM: All-cause mortality; RESP: respiratory mortality; COPD: Chronic Obstructive Pulmonary diseases mortality; ALRI: Acute Lower Respiratory Infections mortality.

A) Identified in the updated systematic review **September 2018 – May 2023**

**Author year**	**Cohort**	**O_3***	**Location**	**Study Period**	**Sample size**	**Sex**	**Age**	**Outcome**
Hvidtfeldt 2019	DCH	Annual	Denmark	1993-2015	49,564	FM	50-64	**ACM**, RESP
Lefler 2019	NHIS	P/W	The United States	1987-2015	635,539	FM	18-84	**ACM**
Lim 2019	NIH-AARP Diet and Health	Annual; P/W	The United States	1995-2011	548,780	FM	50–71	**ACM, RESP, COPD , ALRI**
Pappin 2019	CanCHEC	P/W	Canada	1991-2016	8,500,000	FM	24-89	**ACM**
Kazemiparkouhi 2020	Medicare	P/W	The United States	2000–2008	22,159,190	FM	>=65	ACM, **RESP, COPD , ALRI**
Brunekreef 2021	ELAPSE administrative	P/W	Europe	2000-2017	28,153,138	FM	>=30	**COPD**
Sommar 2021	Umea-VIP	Annual***	Sweden	1990-2014	42,580	FM	40**	**ACM, RESP**
Strak 2021	ELAPSE pooled	P/W	Europe	1990 -2015	325,367	FM	48.7	**ACM, RESP, COPD**
Bauwelinck 2022	Belgian administrative	P/W	Belgium	2001–2011	5,474,470	FM	>=30	ACM, RESP
Byun 2022	NHIS-NSC	Annual; P/W	Republic of Korea	2006–2015	179,806	FM	>=30	**ACM, RESP**
Liu 2022	ELAPSE pooled	P/W	Europe	1990 -2015	325,367	FM	48.7	**ALRI**
Shi 2022	Medicare	P/W	The United States	2001-2017	68,721,015	FM	>=65	**ACM**
So 2022	Danish administrative	P/W	Denmark	2000-2017	3,083,227	FM	>=30	ACM, RESP, COPD ALRI
Stafoggia 2022	ELAPSE administrative	P/W	Europe	2000-2017	28,153,138	FM	>=30	**ACM, RESP**
Vienneau 2023	SNC	P/W	Switzerland	2000-2014	4,188,175	FM	>=30	ACM, RESP
Yuan 2023	CHARLS	Annual; P/W	China	2011-2018	20,882	FM	>=40	**ACM**
Zhang 2023	CLHLS	P/W	China	2005-2018	20,352	FM	>=65	**ACM**

**P/W: Peak/Warm Ozone ** median value, ***1hour Ozone, Bold pairs selected to be included in the meta-analysis*

**Author year**	**Cohort**	**O_3***	**Location**	**Study Period**	**Sample size**	**Sex**	**Age**	**Outcome**
Abbey, 1999	AHSMOG	Annual	The United States	1977-1992	5,652	FM	59.2	**ACM, RESP**
HEI, 2000	Harvard Six Cities	Annual	The United States	1974-1989	8,111	FM	49.7	**ACM**
Lipsett, 2011	CTS	Annual; P/W	The United States	1997-2005	101,784	F	>=30	**ACM, RESP**
Bentayeb, 2015	Gazel cohort	P/W	France	1989-2013	20,327	FM	43.7	**ACM**
Crouse, 2015	CanCHEC	P/W	Canada	1991-2006	2,521,525	FM	25-90	ACM**, RESP, COPD**
Turner, 2016	ACS CPS-II	Annual; P/W	The United States	1982-2004	669,046	FM	>=30	**ACM, RESP, COPD , ALRI**
Di, 2017	Medicare	P/W	The United States	2000-2012	60,925,443	FM	70.1	ACM
Weichenthal, 2017	CanCHEC	P/W	Canada	2001-2011	2,448,500	FM	25-89	ACM, **RESP**
Cakmak, 2018	CanCHEC	P/W	Canada	1991-2011	2,291,250	FM	25-90	ACM, **COPD**

B) Identified in Huangfu & Atkinson (2020) meta-analysis for search **up to September 2018**.

**P/W: Peak/Warm Ozone Bold pairs selected to be included in the meta-analysis*

Figure S1. Funnel plot with Egger's test p-value for the meta-analysis of the association between all-cause mortality and 10μg/m^3 increase in NO_2 (Global, 2023-2024).

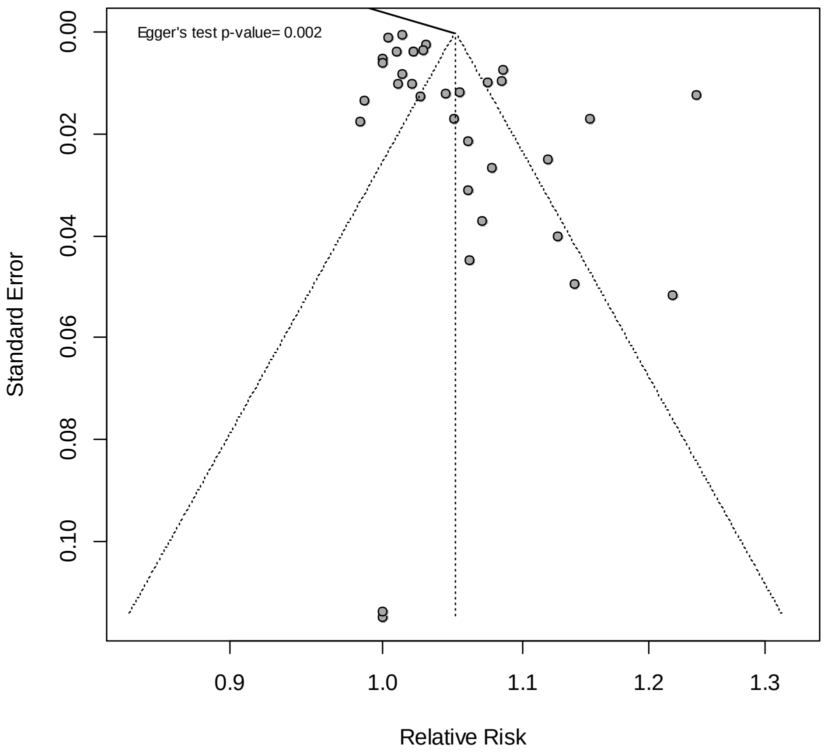

Figure S2. Forest plot for the relative risk (RR) in all-cause mortality associated with 10μg/m³ increase in NO_2 by WHO region (Global, 2023-2024).

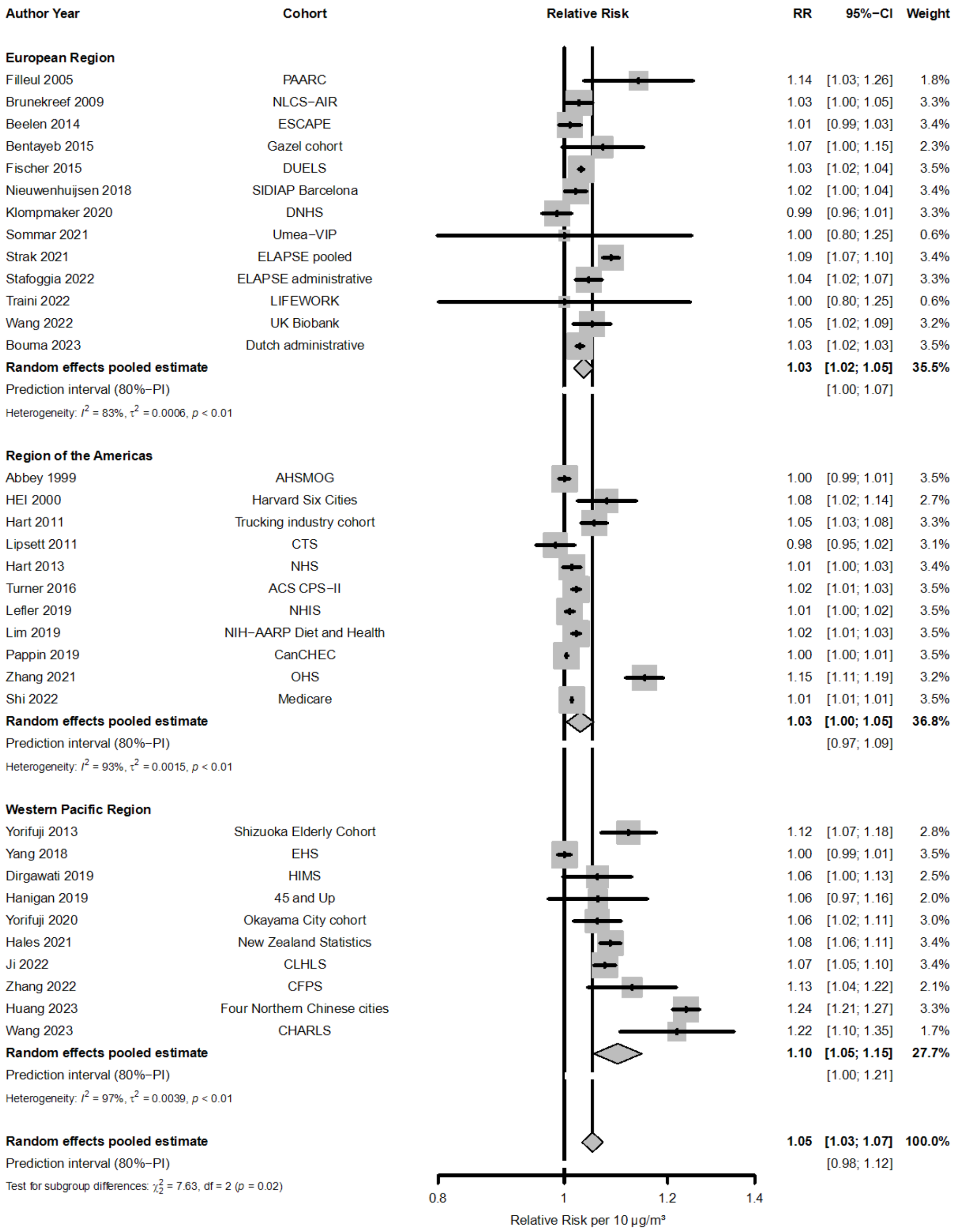

Figure S3. Forest plot for the relative risk (RR) in all-cause mortality associated with 10μg/m^3 increase in NO_2 by RoB assessment in the confounding domain (Global, 2023-2024)..

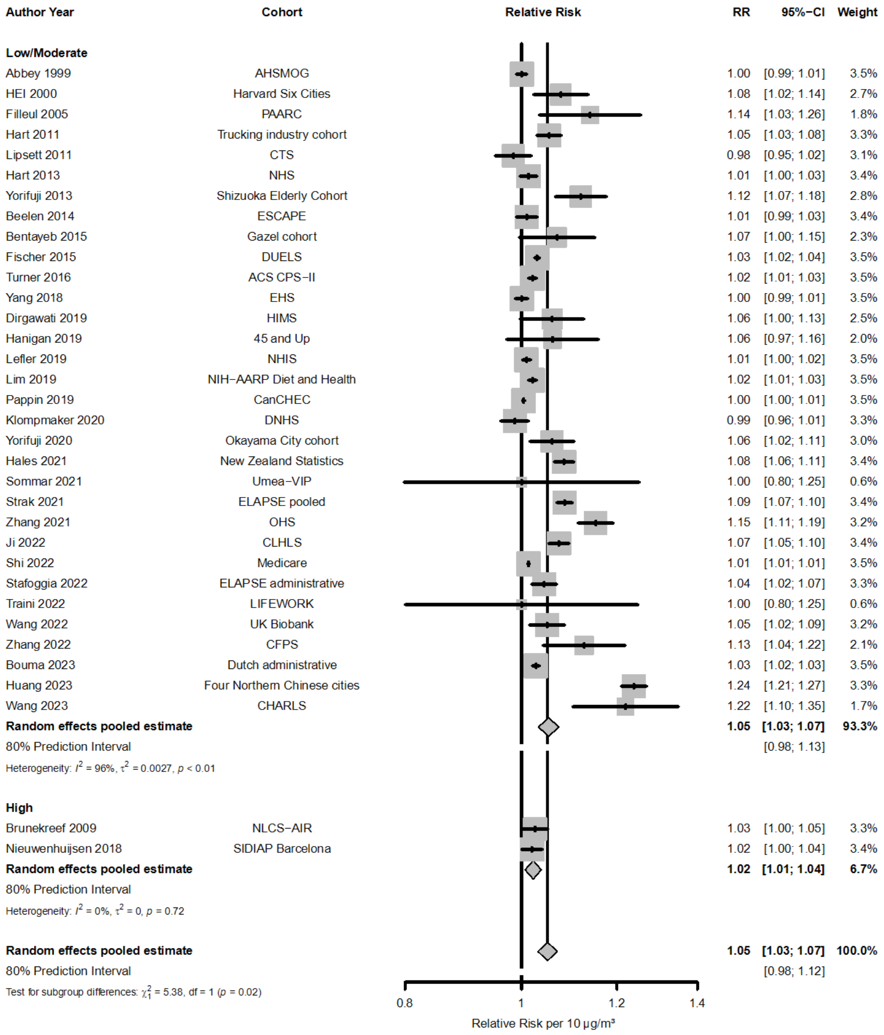

Figure S4. Forest plot for the relative risk (RR) in all-cause mortality associated with 10µg/m^3 increase in NO_2 by administrative cohorts or cohorts with detailed individual data (Global, 2023-2024).

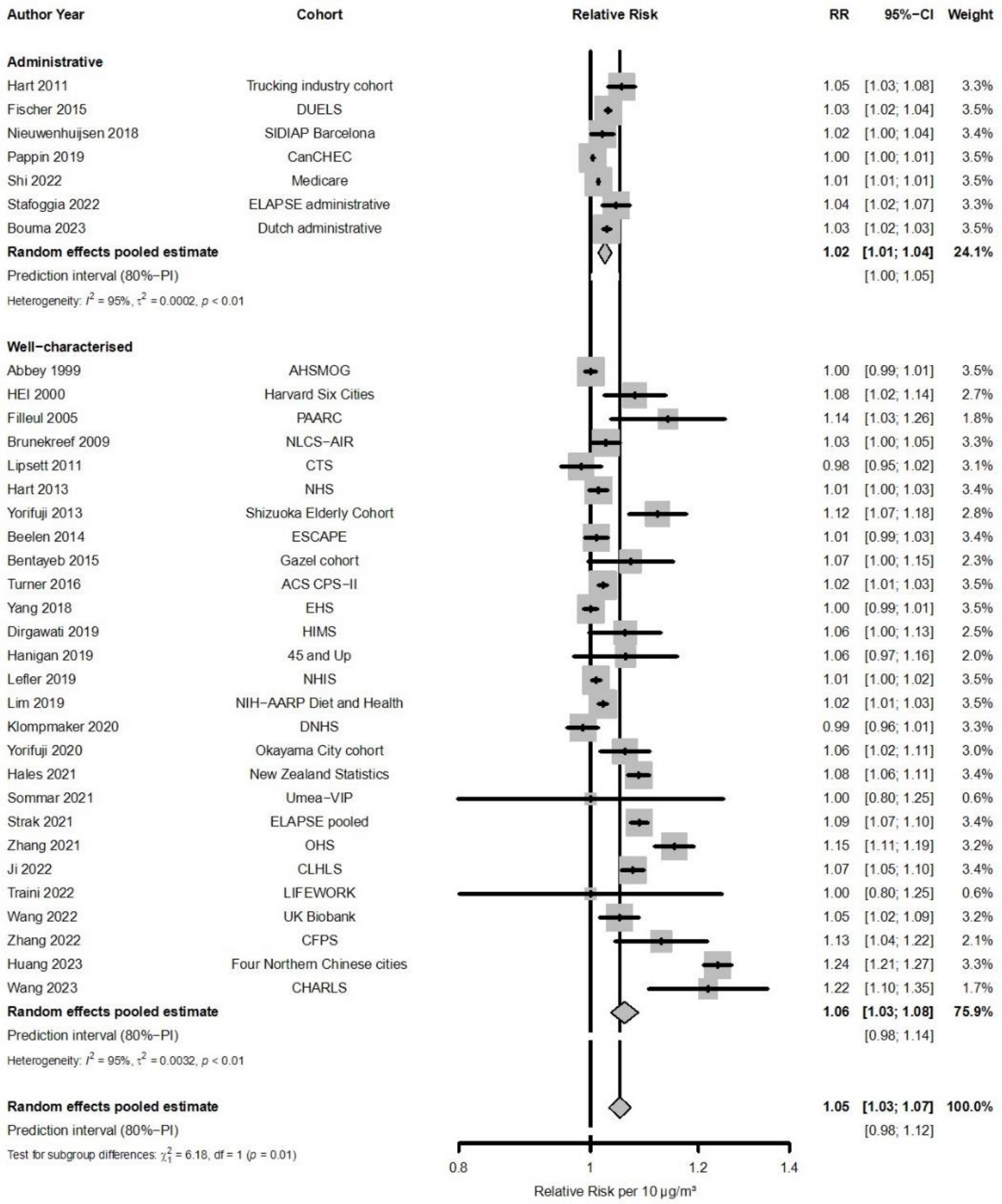

Figure S5. Forest plot of 28 studies on the risk of circulatory mortality associated with 10μg/m³ increase in NO_2 (Global, 2023-2024).

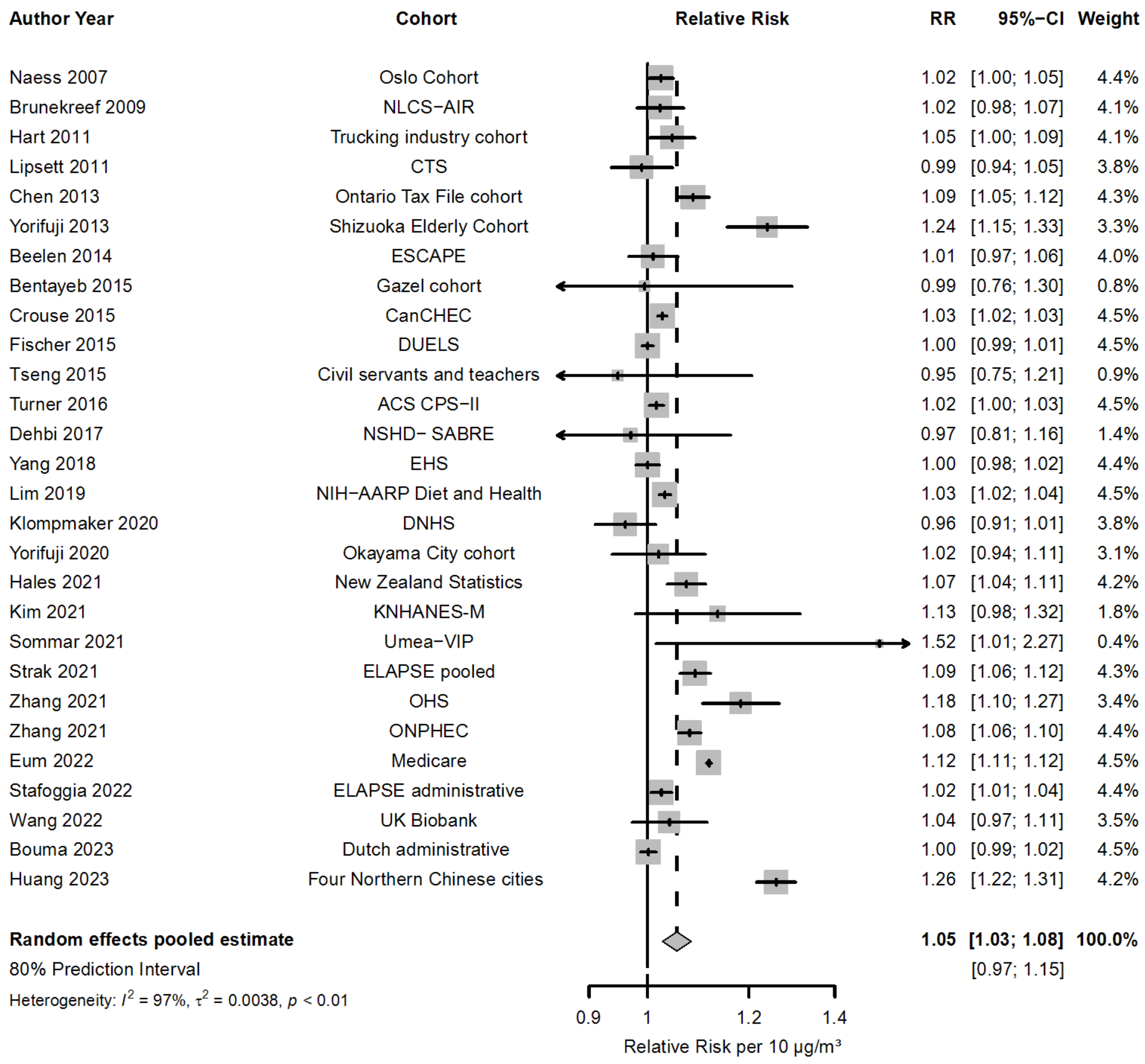

Figure S6. Funnel plot with Egger's test p-value for the association between circulatory mortality and 10μg/m^3 increase in NO_2 (Global, 2023-2024).

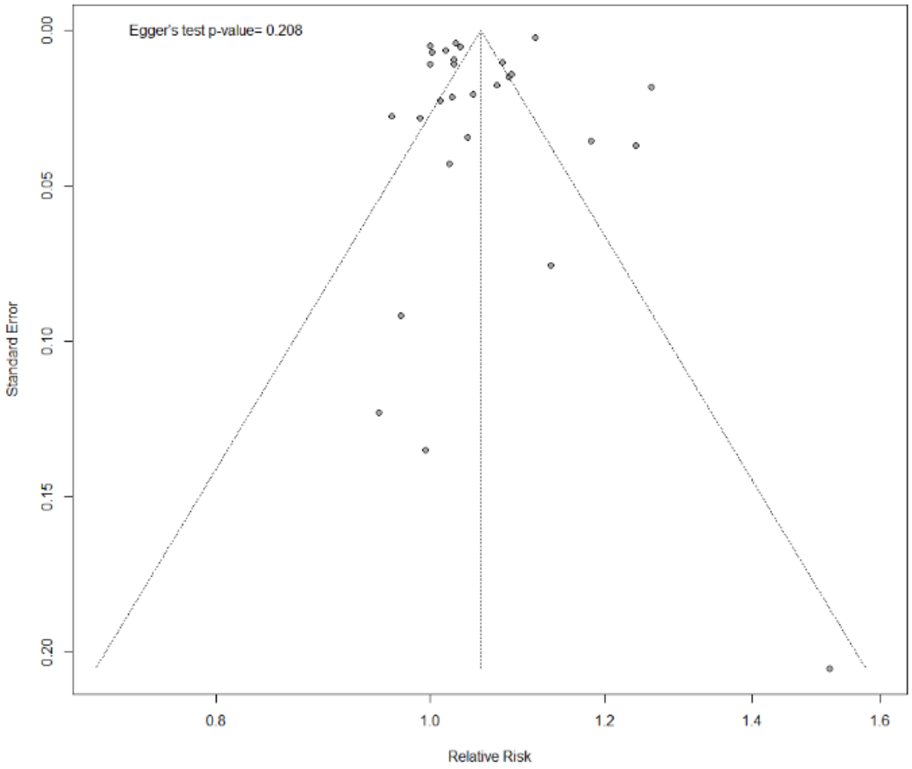

Figure S7. Forest plot for the relative risk (RR) in circulatory mortality associated with 10μg/m^3 increase in NO_2 by WHO region (Global, 2023-2024).

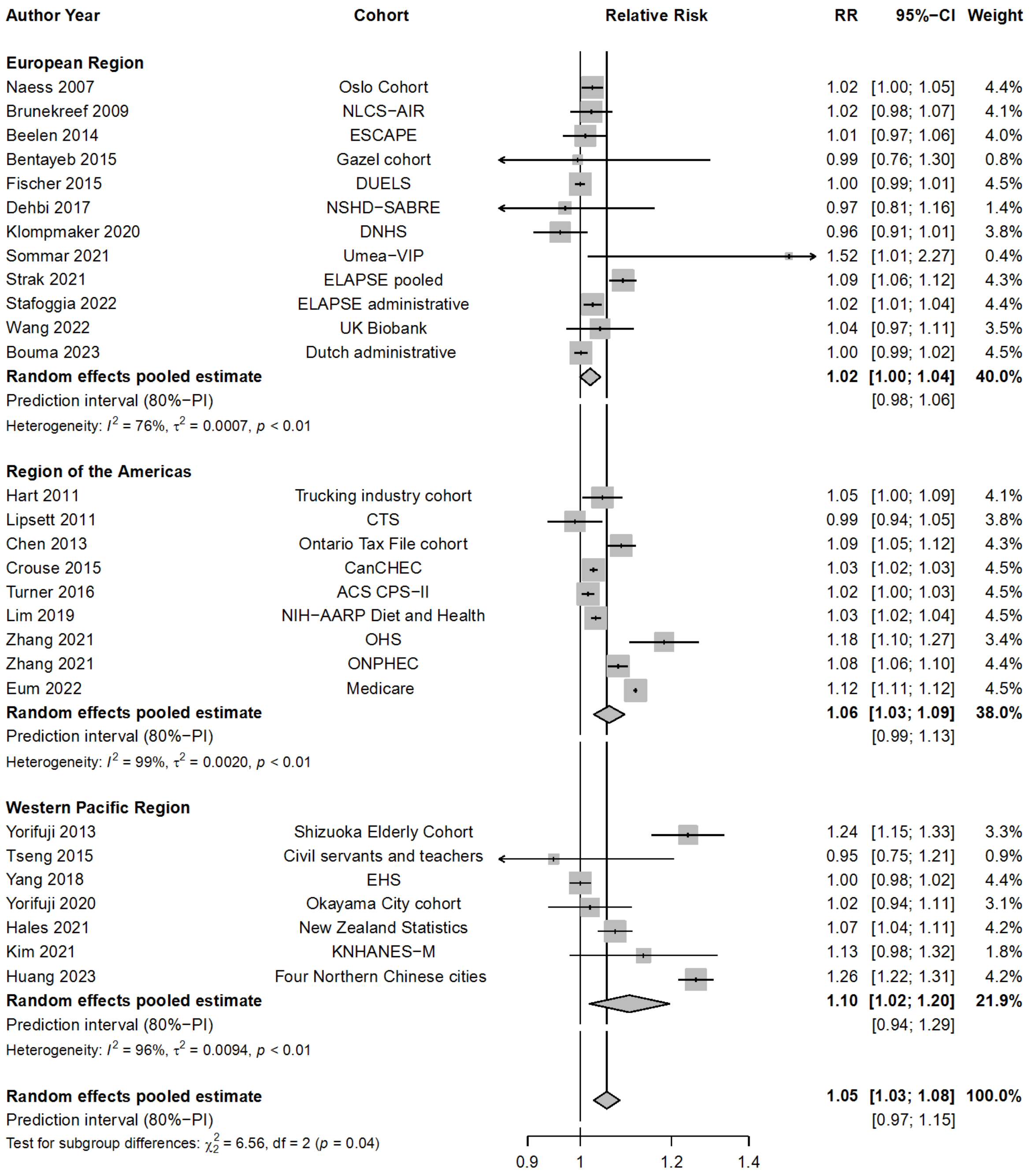

Figure S8. Forest plot for the relative risk (RR) in circulatory mortality associated with 10µg/m³ increase in NO_2 by RoB assessment in the confounding domain (Global, 2023-2024).

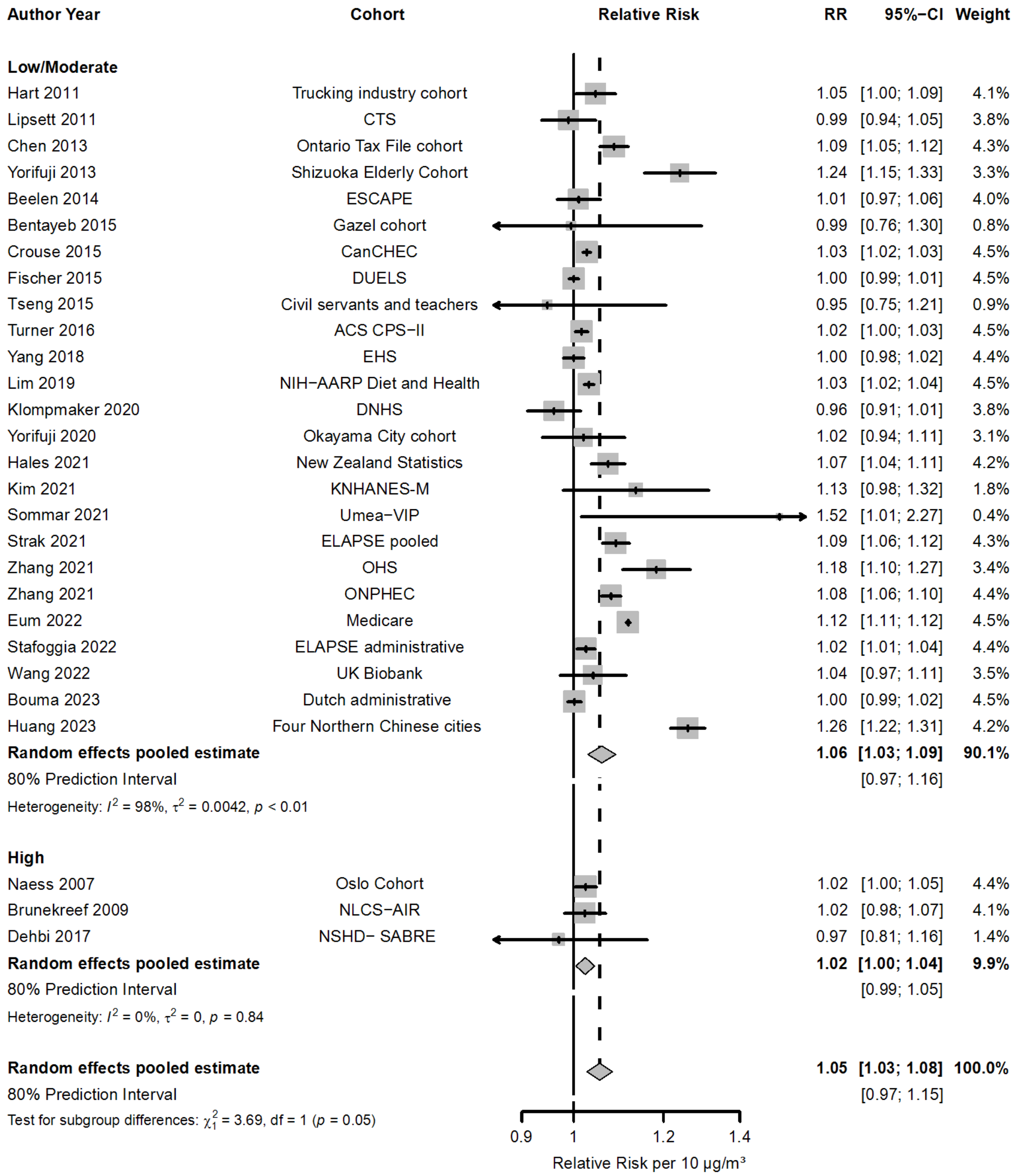

Figure S9. Forest plot of 20 studies on risk of ischemic heart disease mortality associated with 10μg/m^3 increase in NO_2 (Global, 2023-2024).

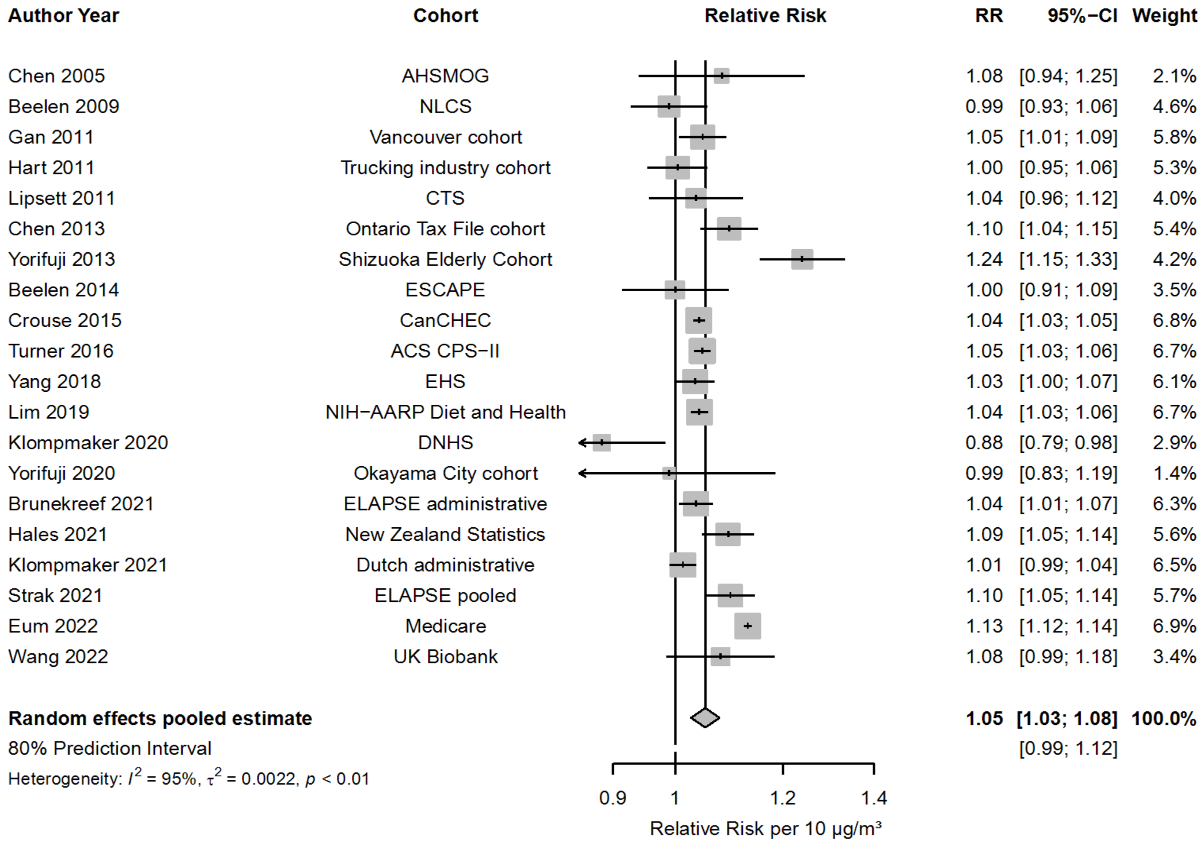

Figure S10. Funnel plot with Egger's test p-value for the association ischemic heart disease mortality and 10μg/m^3 increase in NO_2 (Global, 2023-2024).

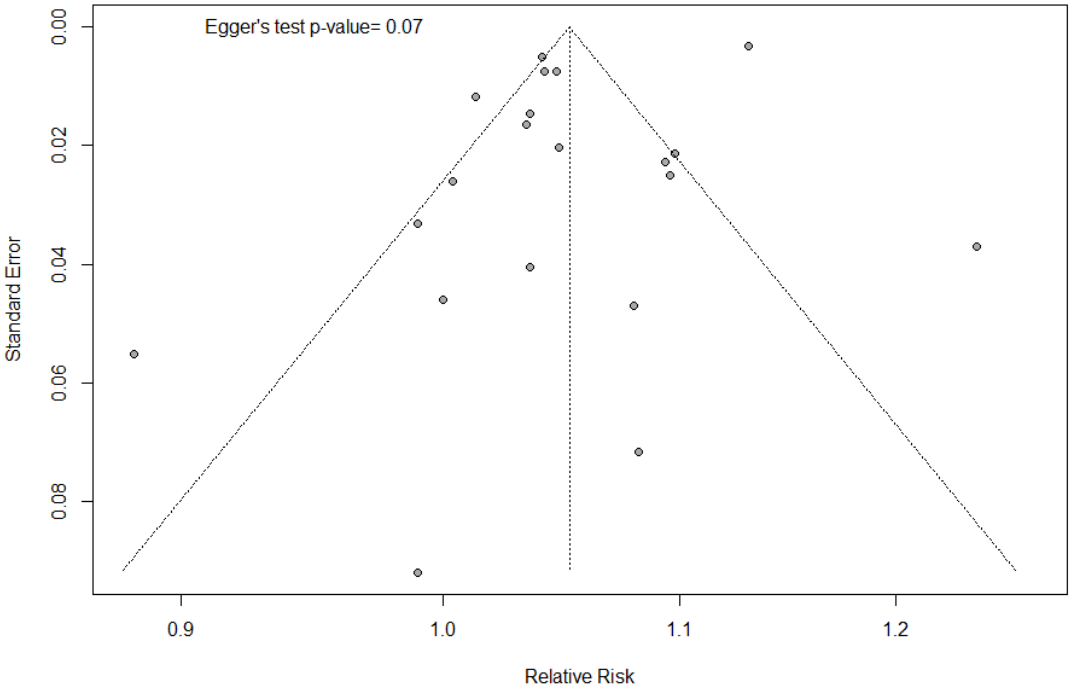

Figure S11. Forest plot for the relative risk (RR) in ischemic heart disease mortality associated with 10μg/m^3 increase in NO_2 by WHO region (Global, 2023-2024).

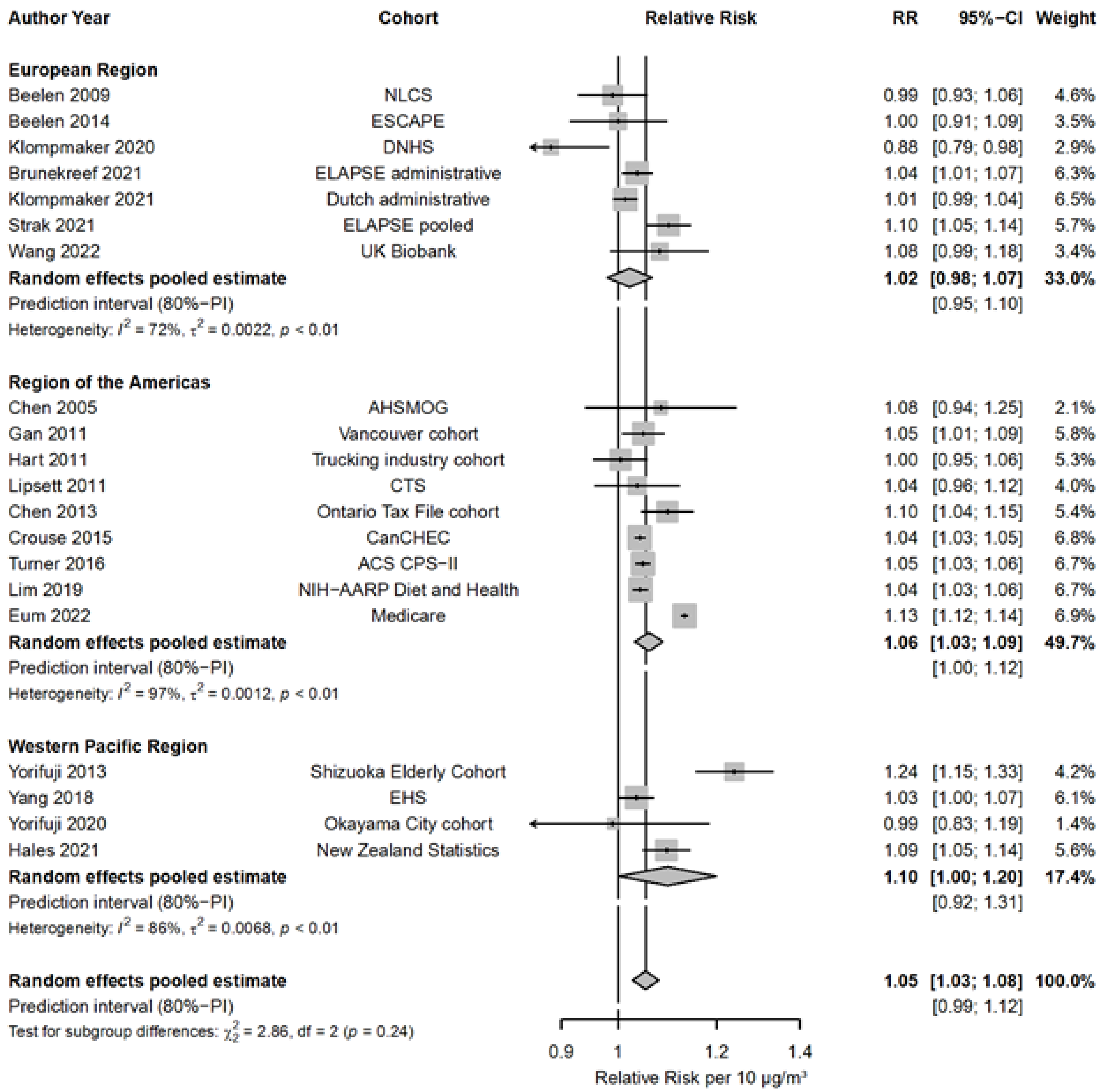

Figure S12. Forest plot of 20 studies on risk of cerebrovascular disease mortality associated with 10μg/m^3 increase in NO_2 (Global, 2023-2024).

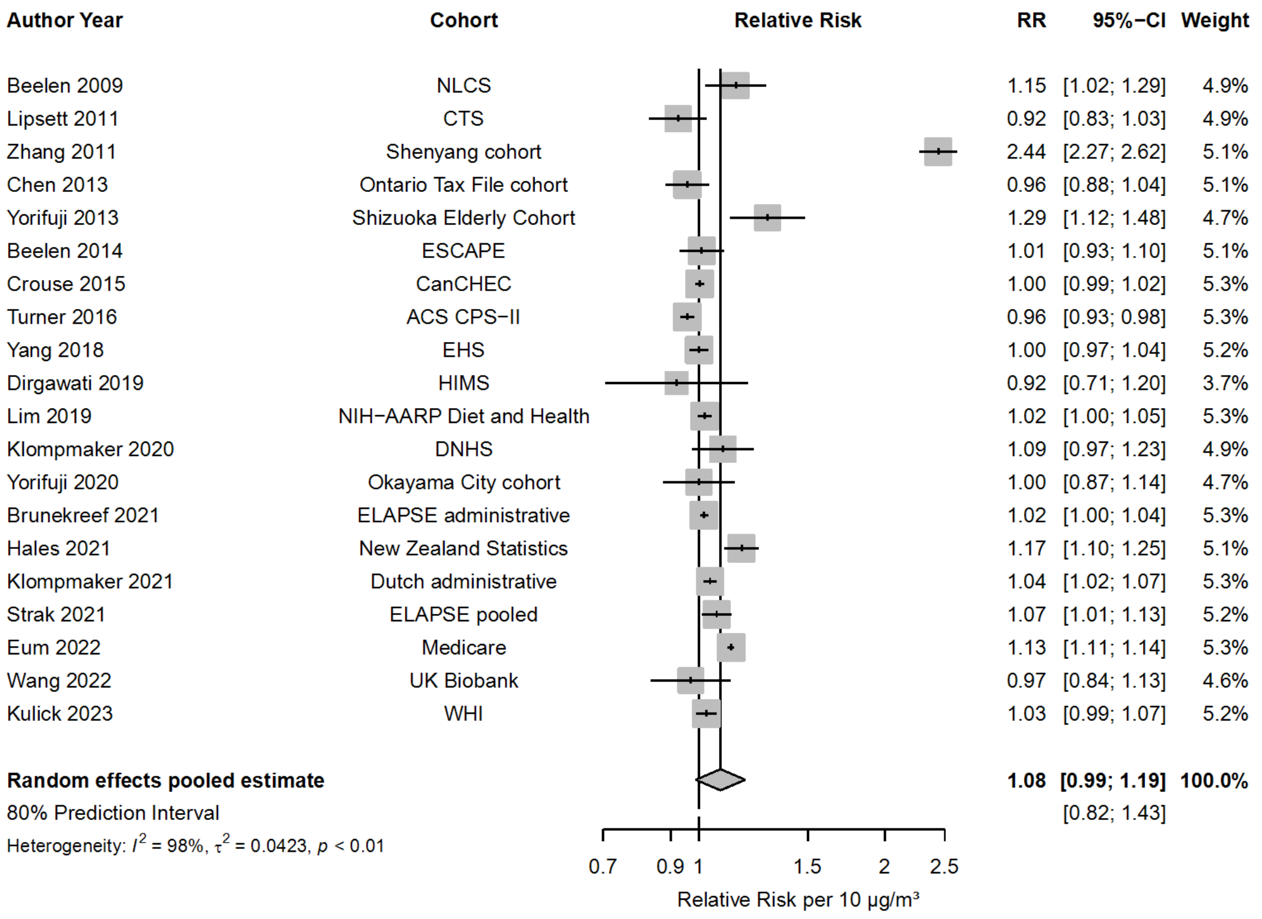

Figure S13. Funnel plot with Egger's test p-value for the association cerebrovascular disease mortality and 10μg/m^3 increase in NO_2 (Global, 2023-2024).

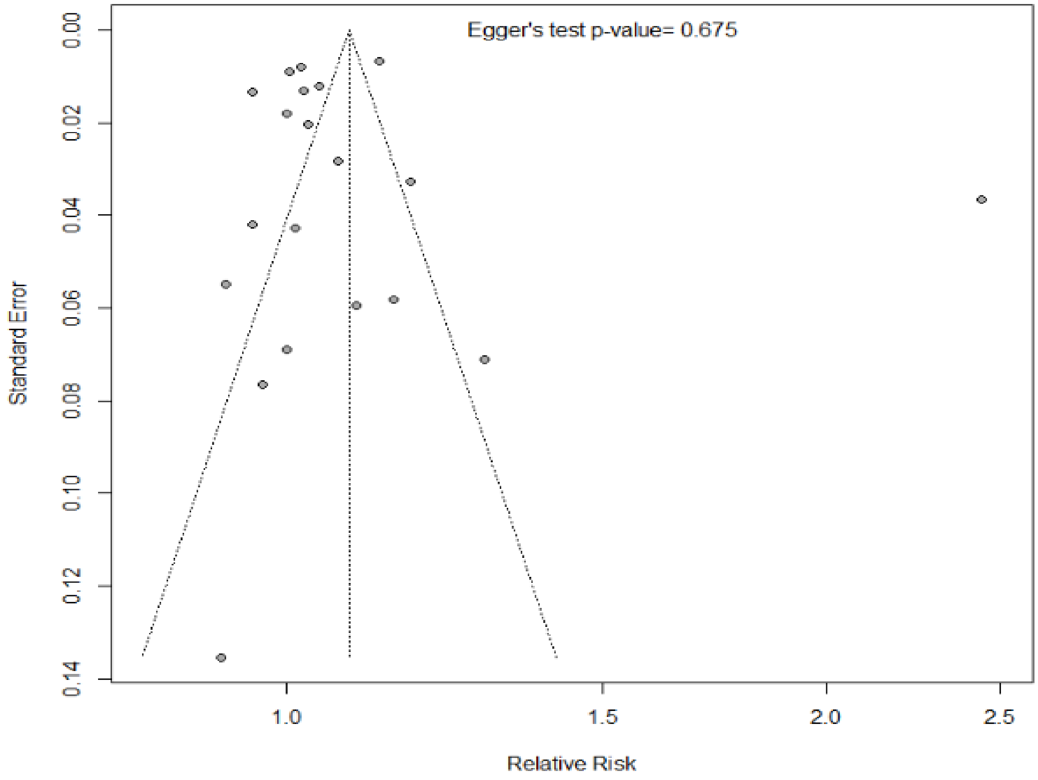

Figure S14. Forest plot for the relative risk (RR) in cerebrovascular disease mortality associated with 10μg/m³ increase in NO_2 by WHO region (Global, 2023-2024).

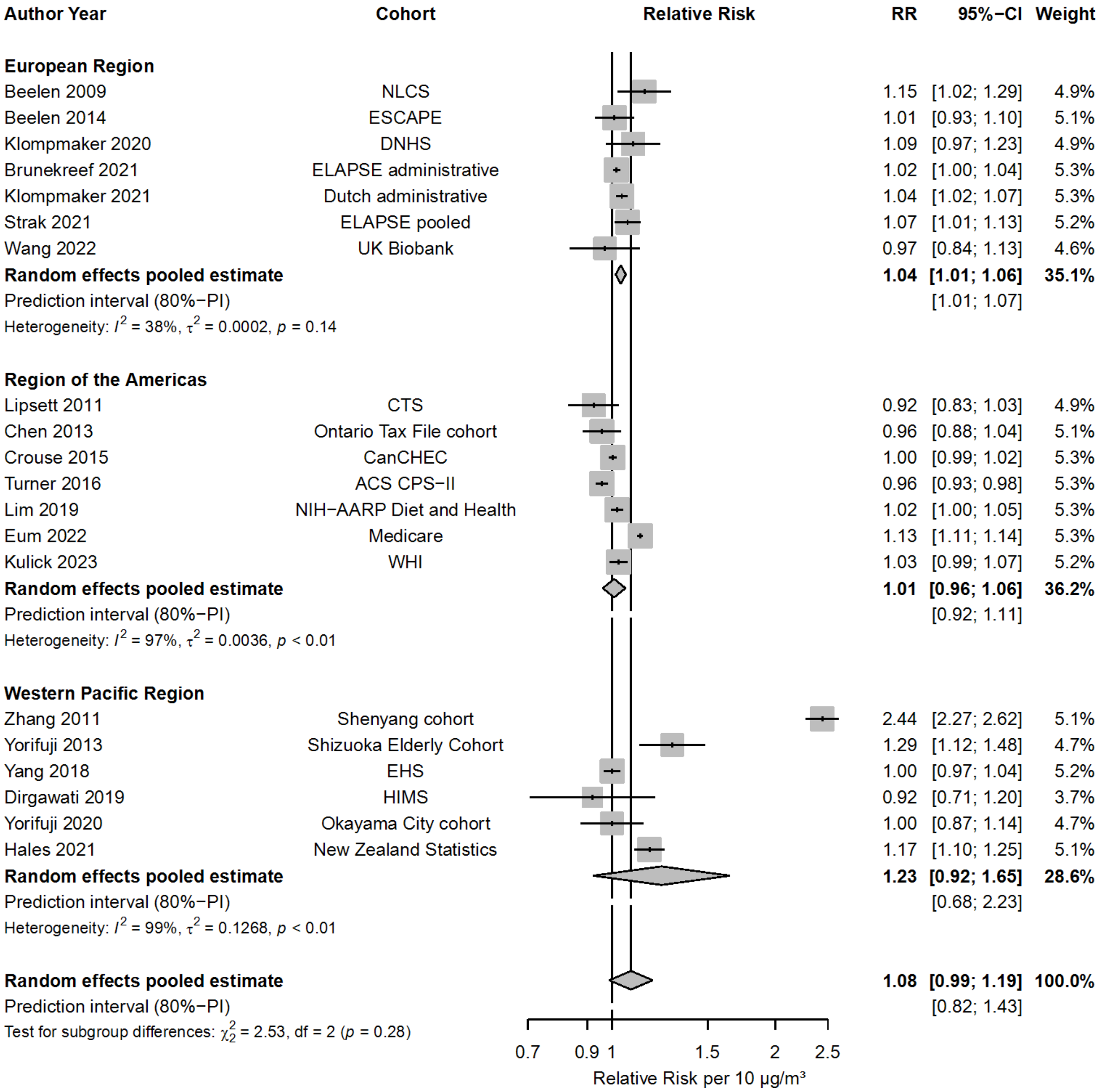

Figure S15. Forest plot for the relative risk (RR) in cerebrovascular disease mortality associated with 10µg/m^3 increase in NO_2 by RoB assessment in the confounding domain (Global, 2023-2024).

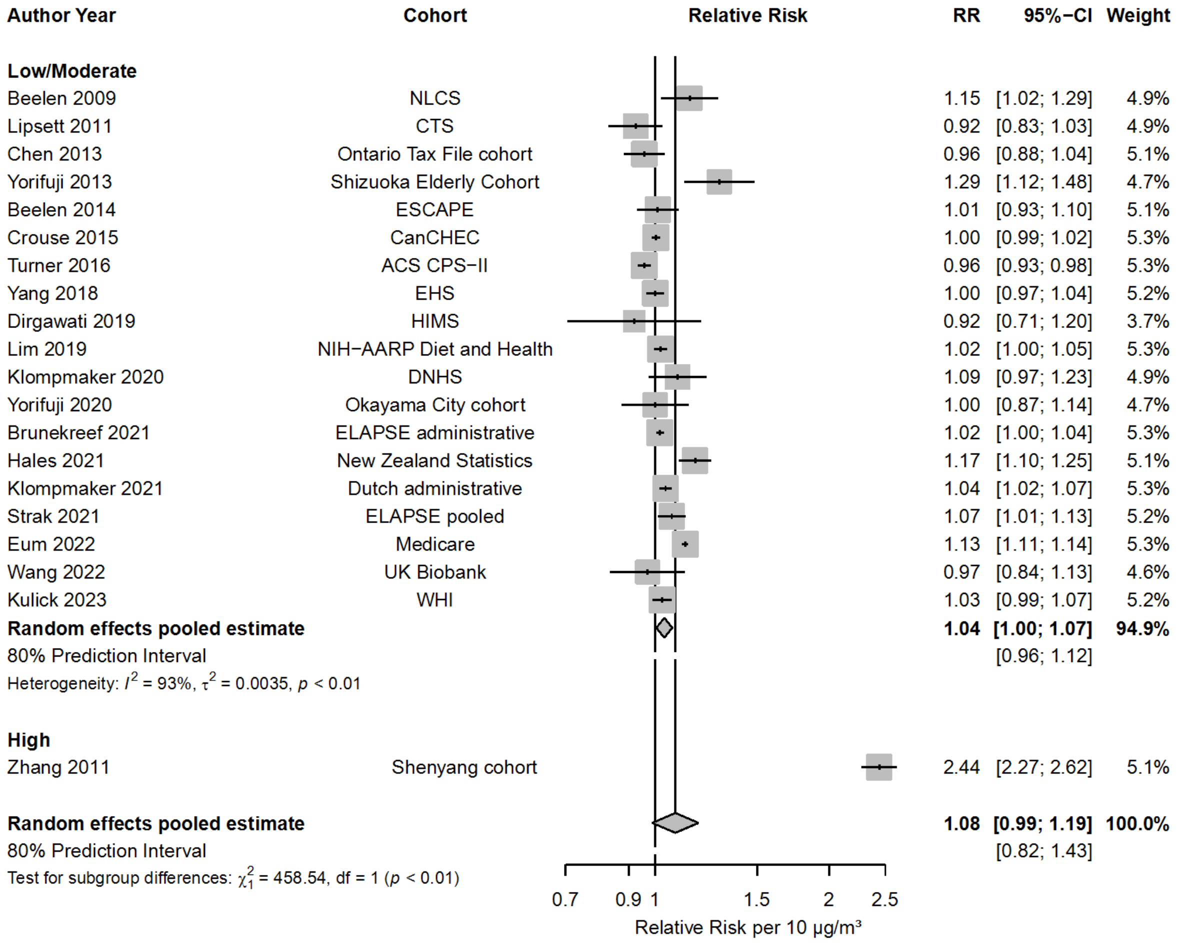

Figure S16. Forest plot of 25 studies on risk of respiratory disease mortality associated with 10μg/m^3 increase in NO_2 (Global, 2023-2024).

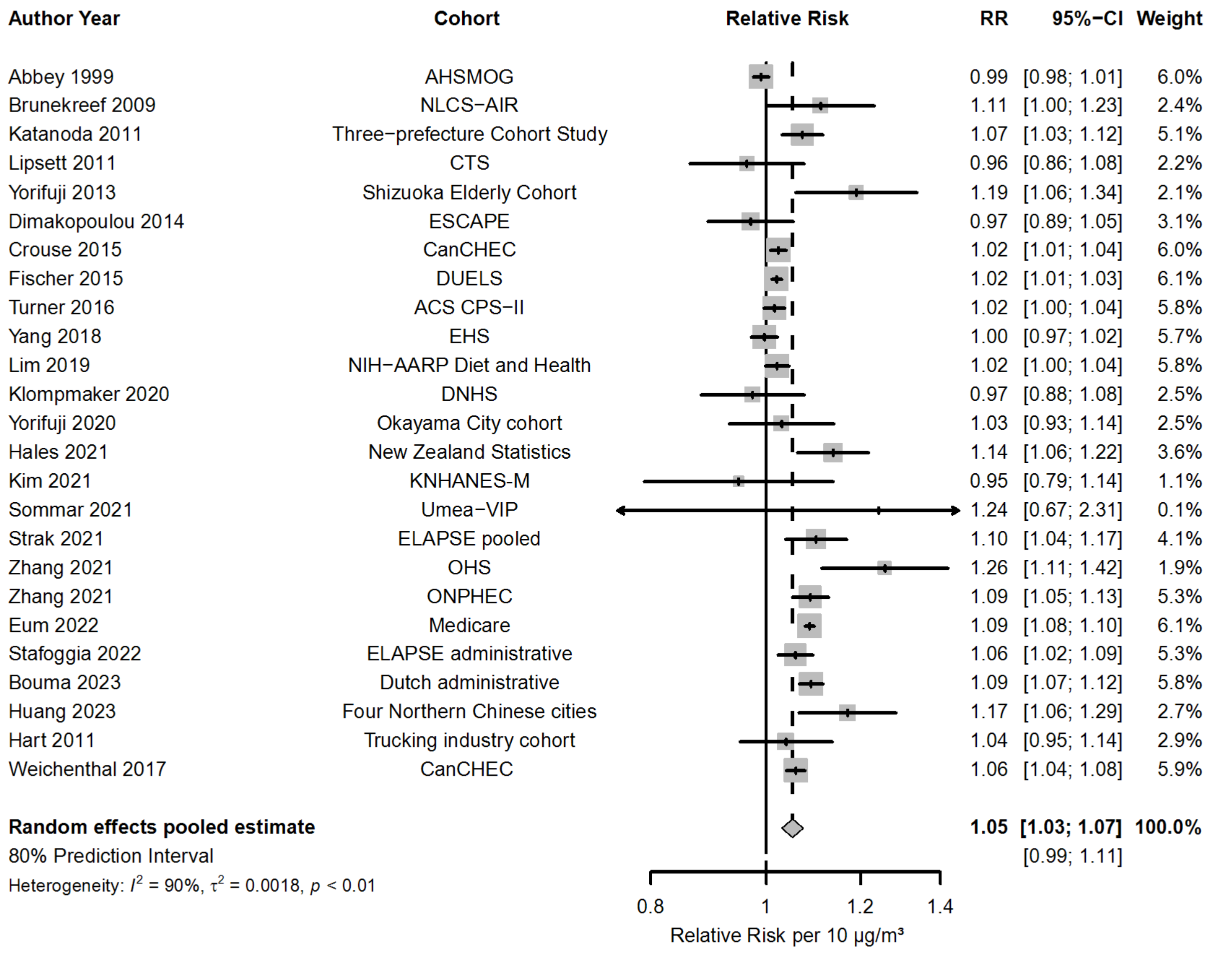

Figure S17. Funnel plot with Egger's test p-value for the association respiratory disease mortality and 10μg/m^3 increase in NO_2 (Global, 2023-2024).

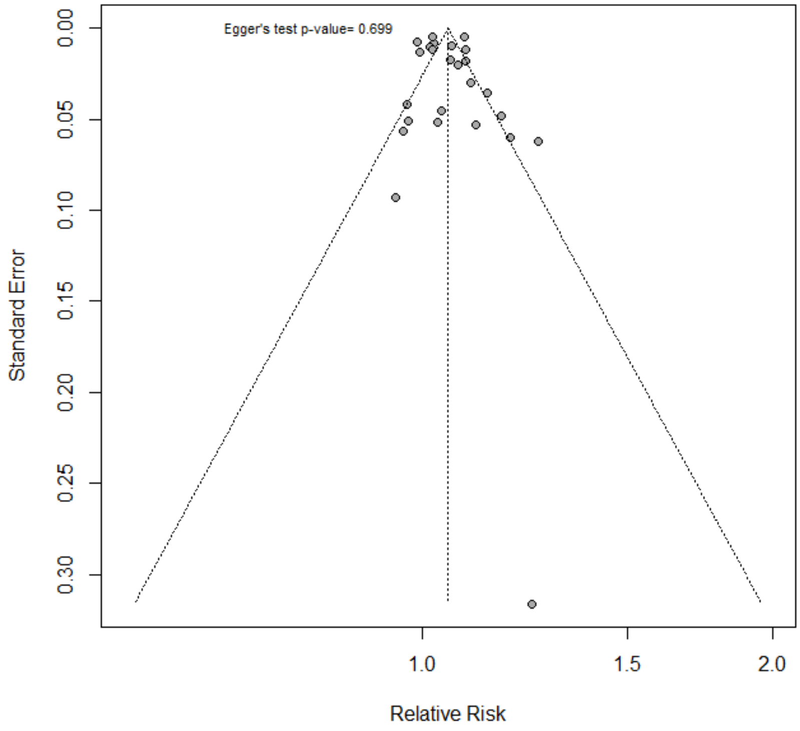

Figure S18. Forest plot for the relative risk (RR) in respiratory disease mortality associated with 10μg/m^3 increase in NO_2 by WHO region (Global, 2023-2024).

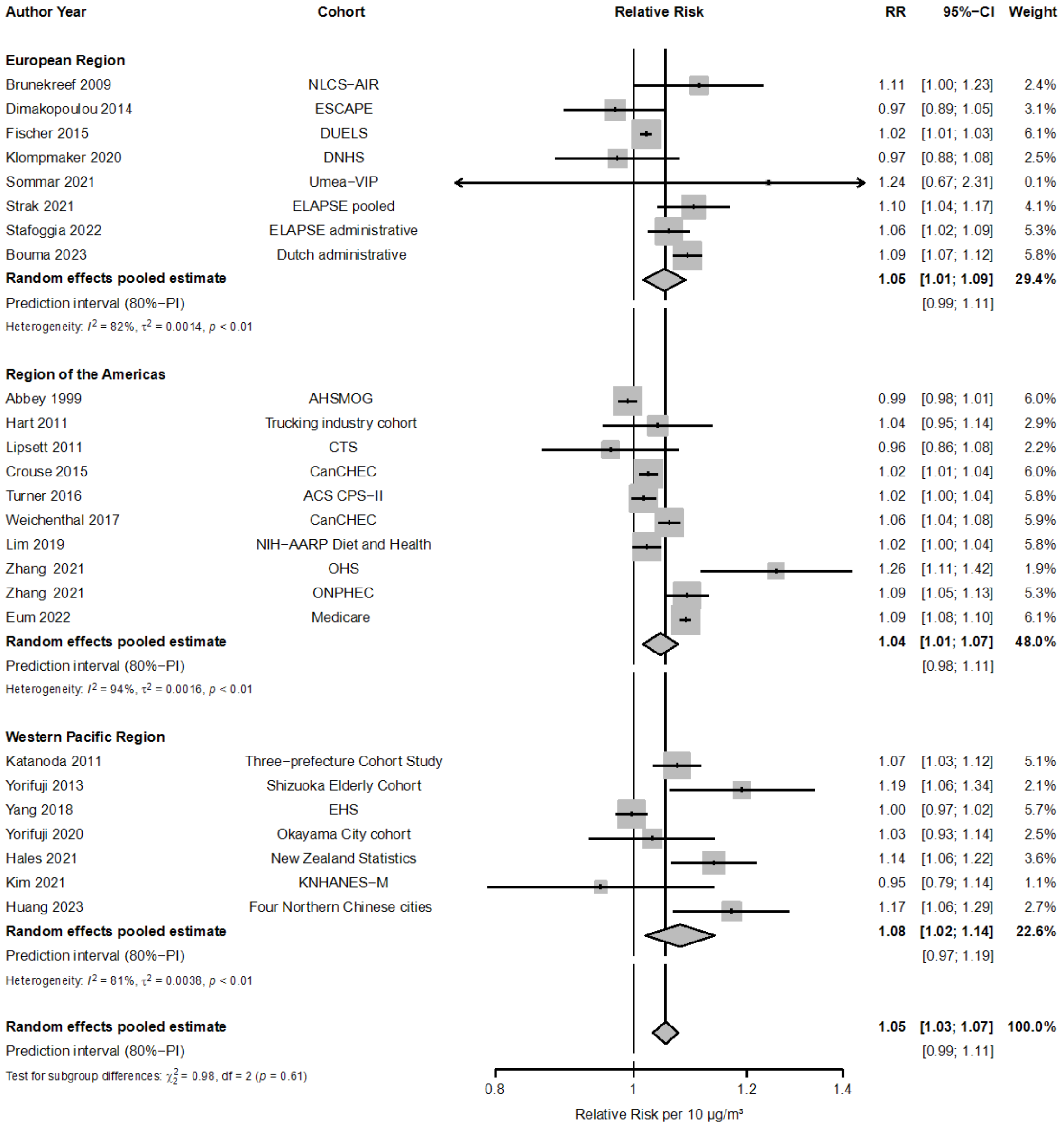

Figure S19. Forest plot for the relative risk (RR) in respiratory disease mortality associated with 10μg/m^3 increase in NO_2 by RoB assessment in the confounding domain (Global, 2023-2024).

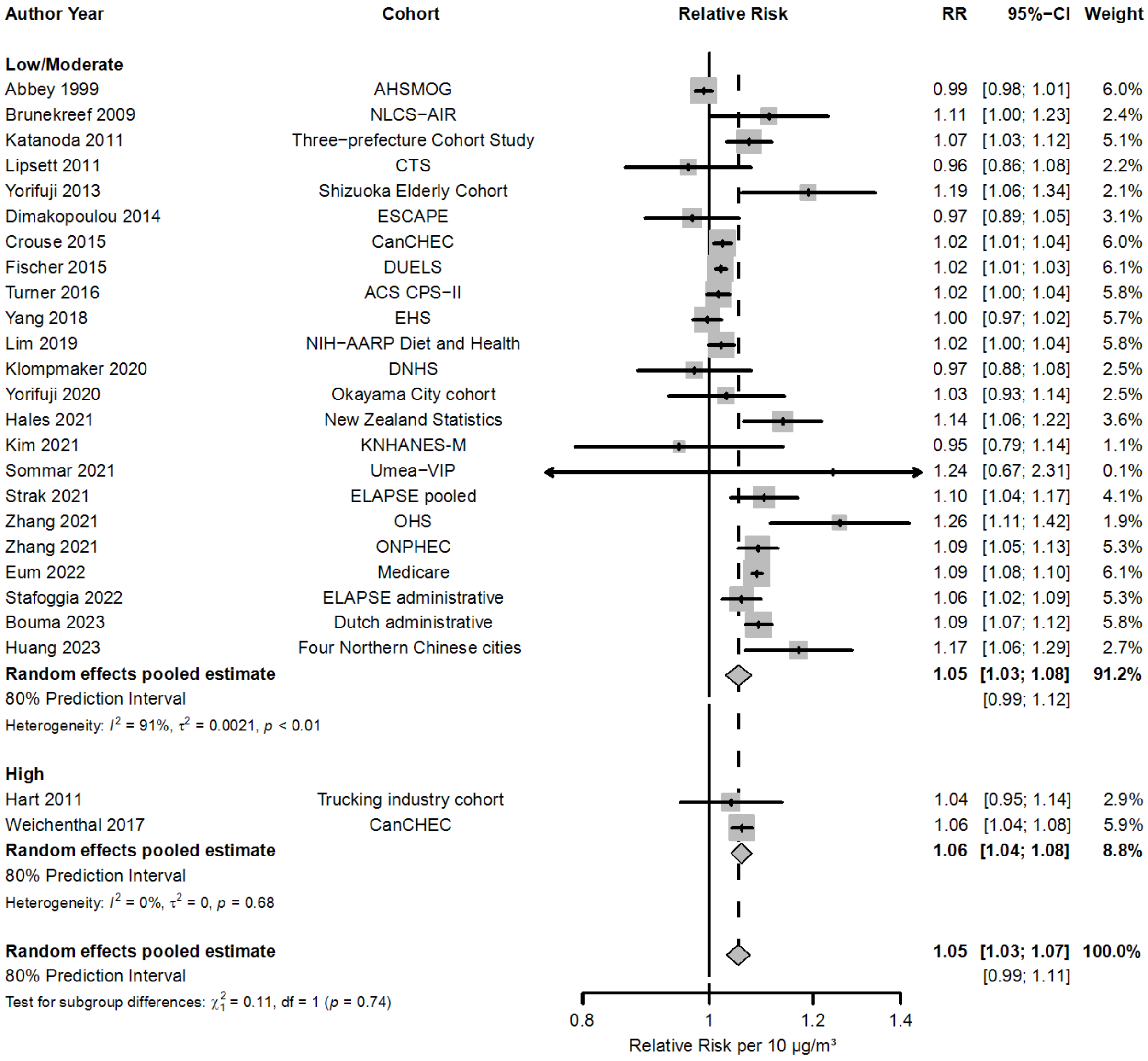

.

Figure S20. Forest plot of 15 studies on risk of COPD mortality associated with 10μg/m^3 increase in NO_2 (Global, 2023-2024).

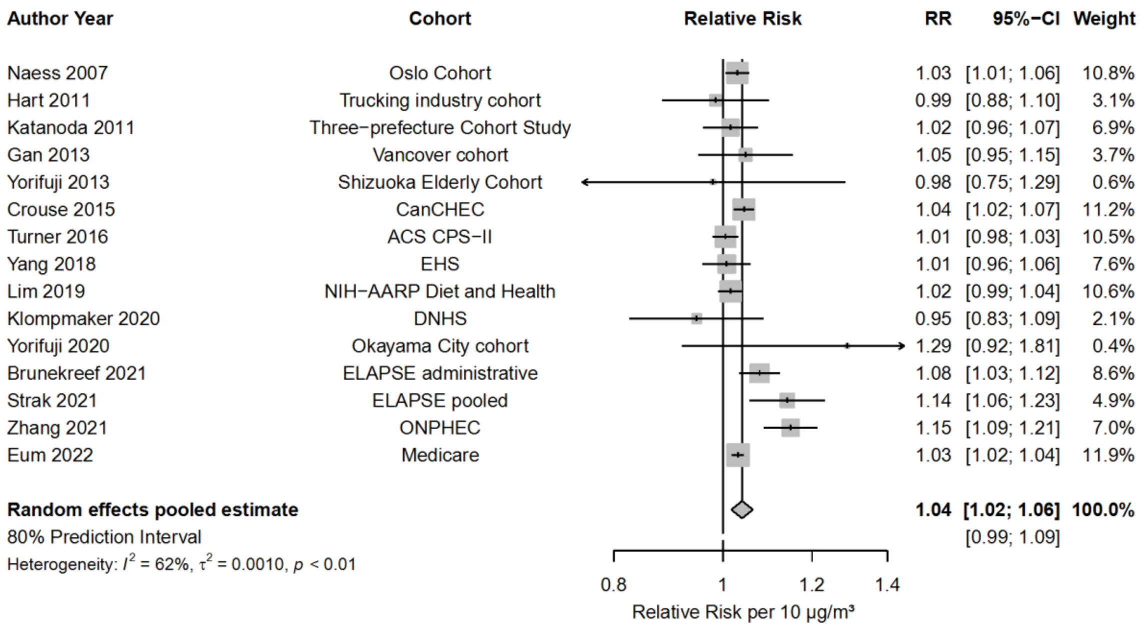

.

Figure S21. Funnel plot with Egger's test p-value for the association between COPD mortality and 10μg/m^3 increase in NO_2 (Global, 2023-2024).

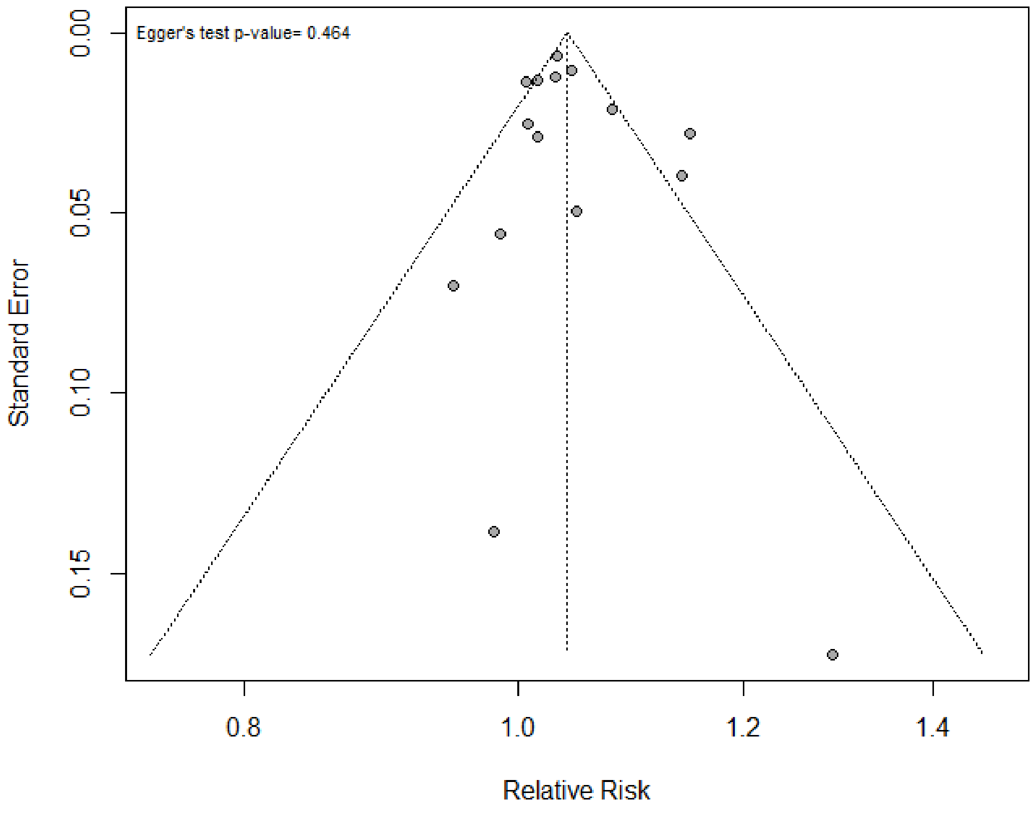

Figure S22. Forest plot for the relative risk (RR) in COPD mortality associated with 10μg/m³ increase in NO_2 by WHO region (Global, 2023-2024).

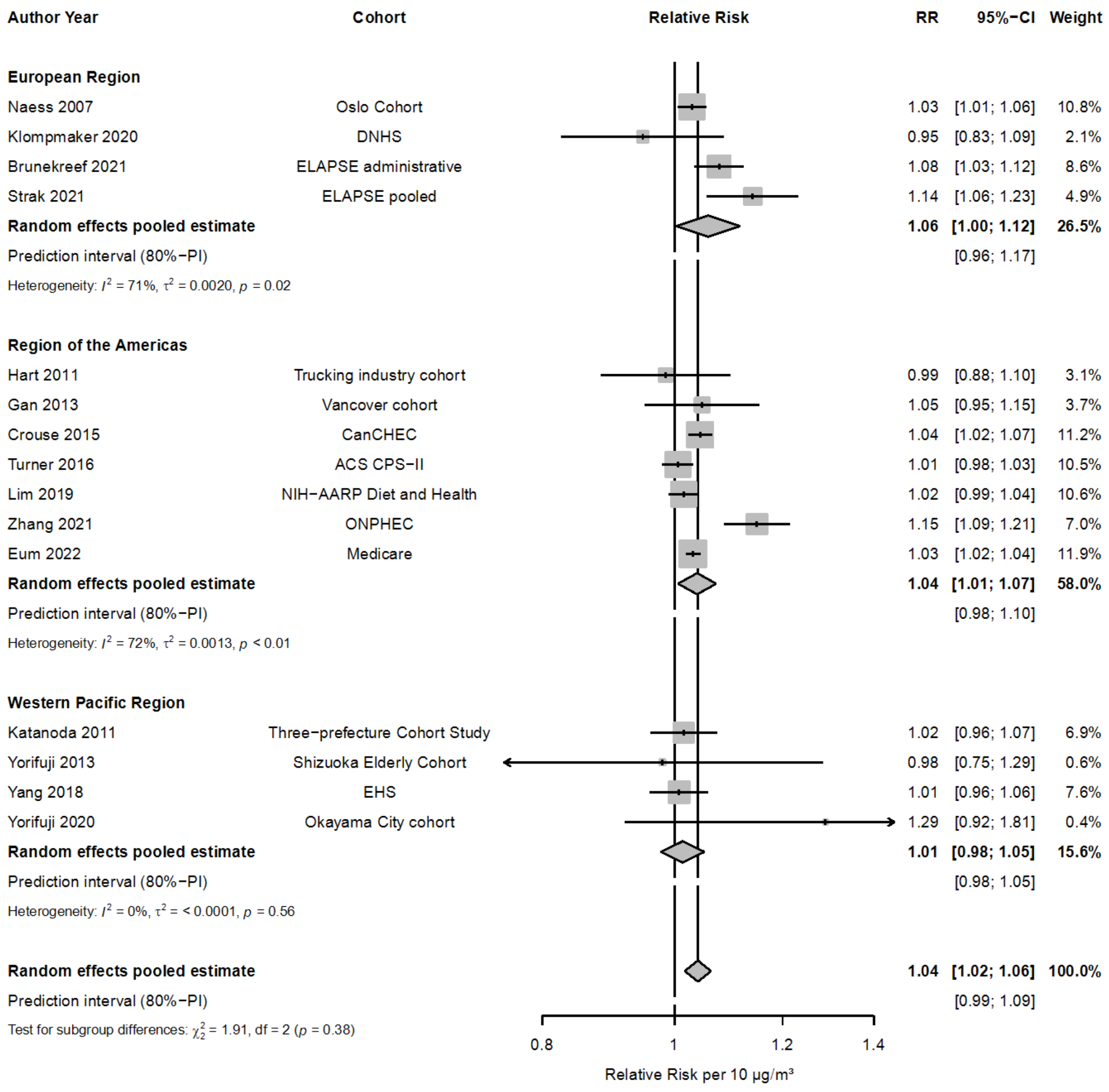

Figure S23. Forest plot for the relative risk (RR) in COPD mortality associated with 10μg/m^3 increase in NO_2 by RoB assessment in the confounding domain (Global, 2023-2024).

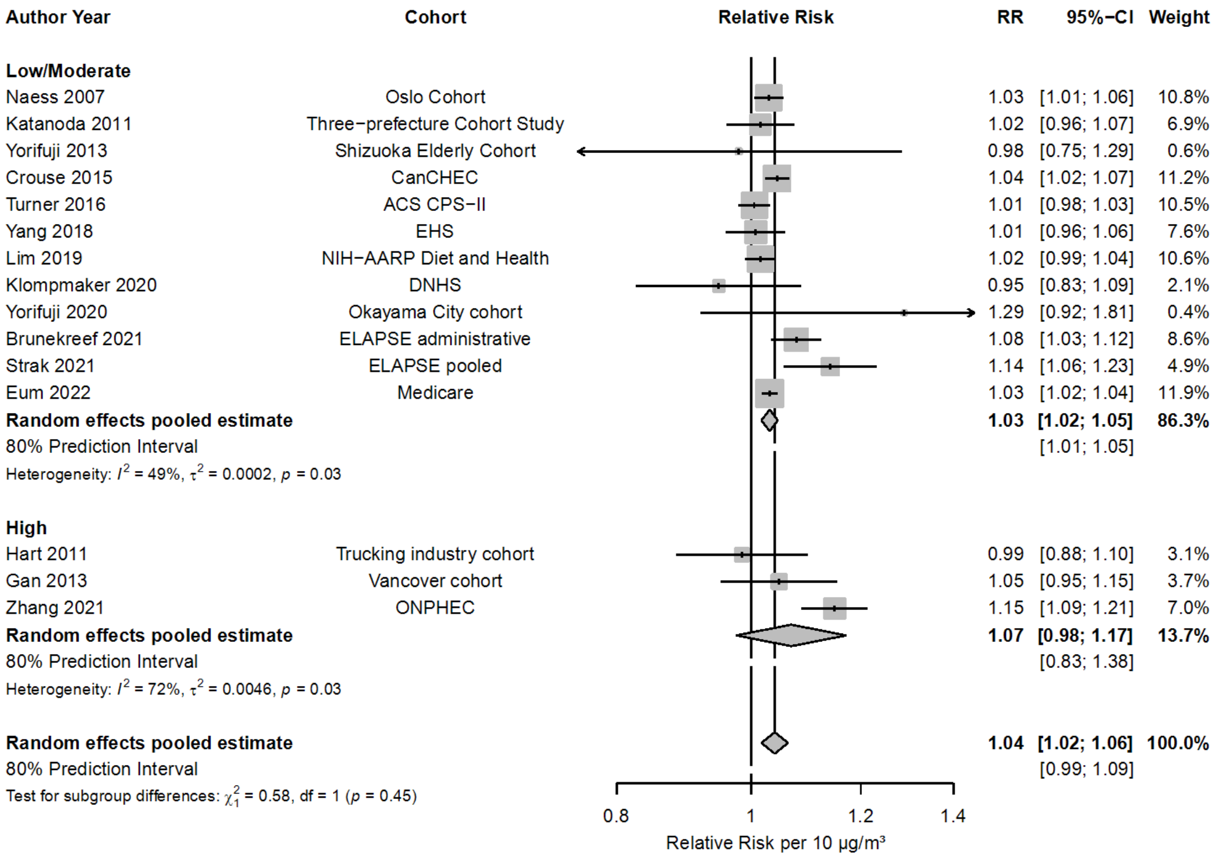

Figure S24. Forest plot of 9 studies on risk of ALRI mortality associated with 10μg/m^3 increase in NO_2 (Global, 2023-2024).

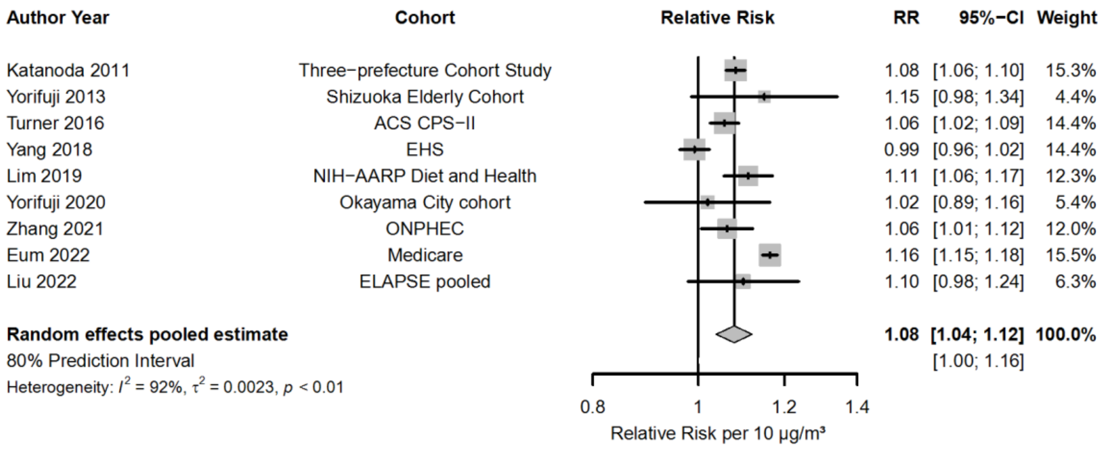

.

Figure S25. Funnel plot for the meta-analysis of the association between ALRI mortality and 10µg/m^3 increase in NO_2 (Global, 2023-2024).

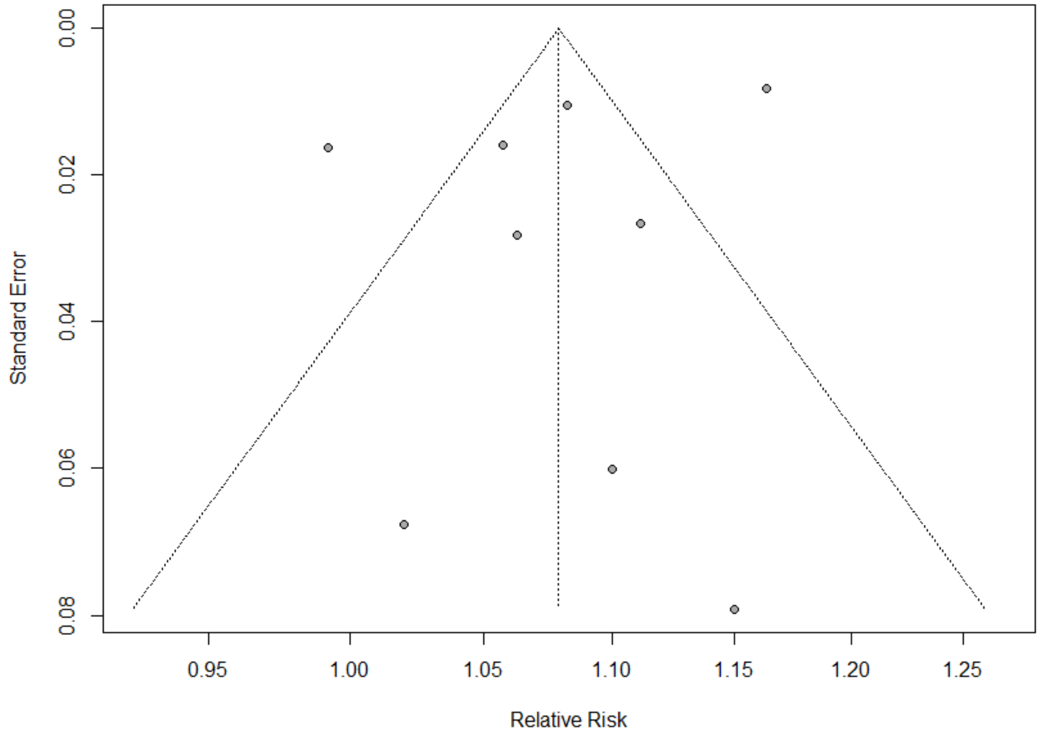

Figure S26. Forest plot for the relative risk (RR) in ALRI mortality associated with 10μg/m³ increase in NO_2 by WHO region (Global, 2023-2024).

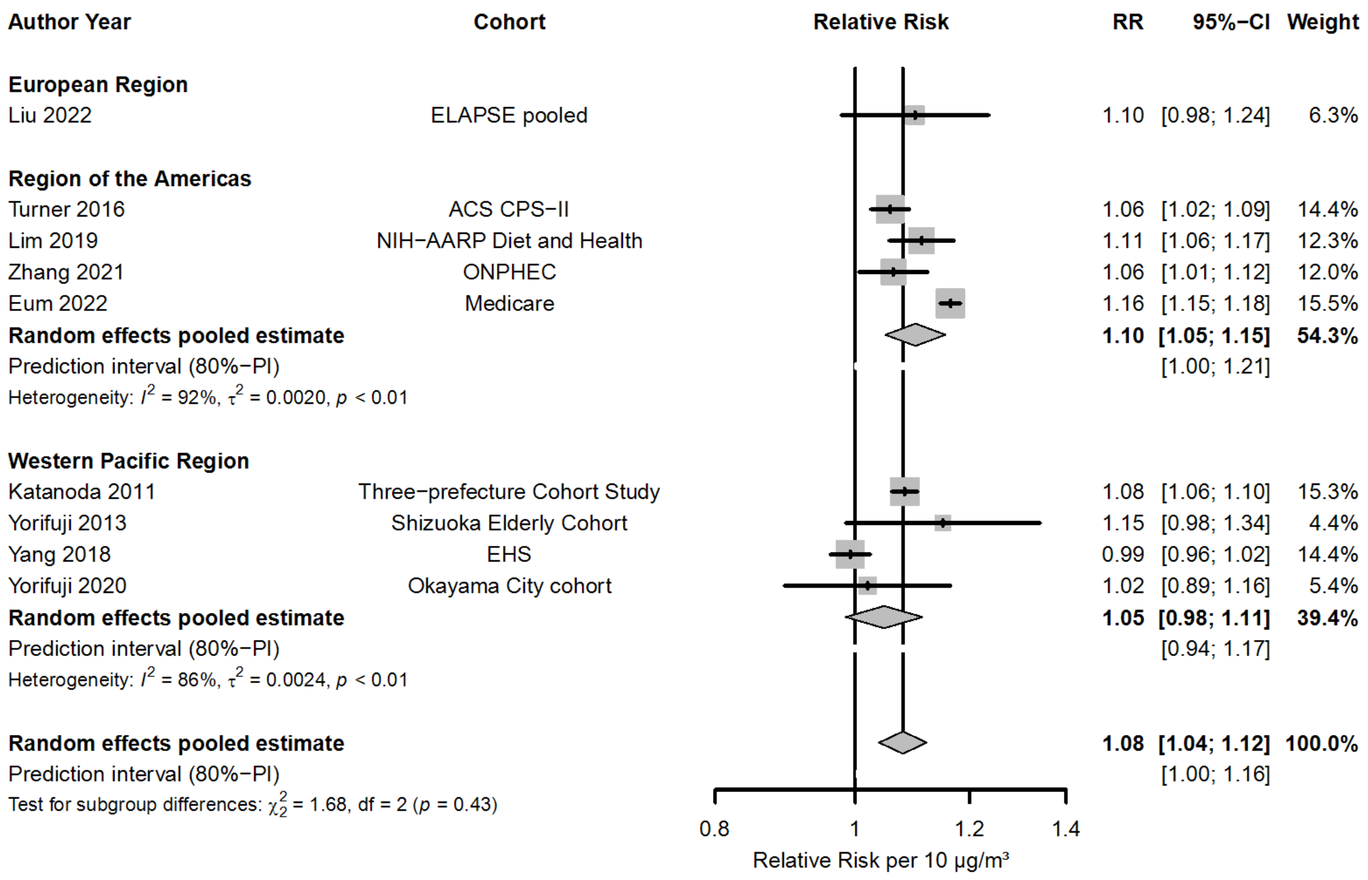

Figure S27. Forest plot for the relative risk (RR) in ALRI mortality associated with 10μg/m^3 increase in NO_2 by NO_2 levels (Global, 2023-2024).

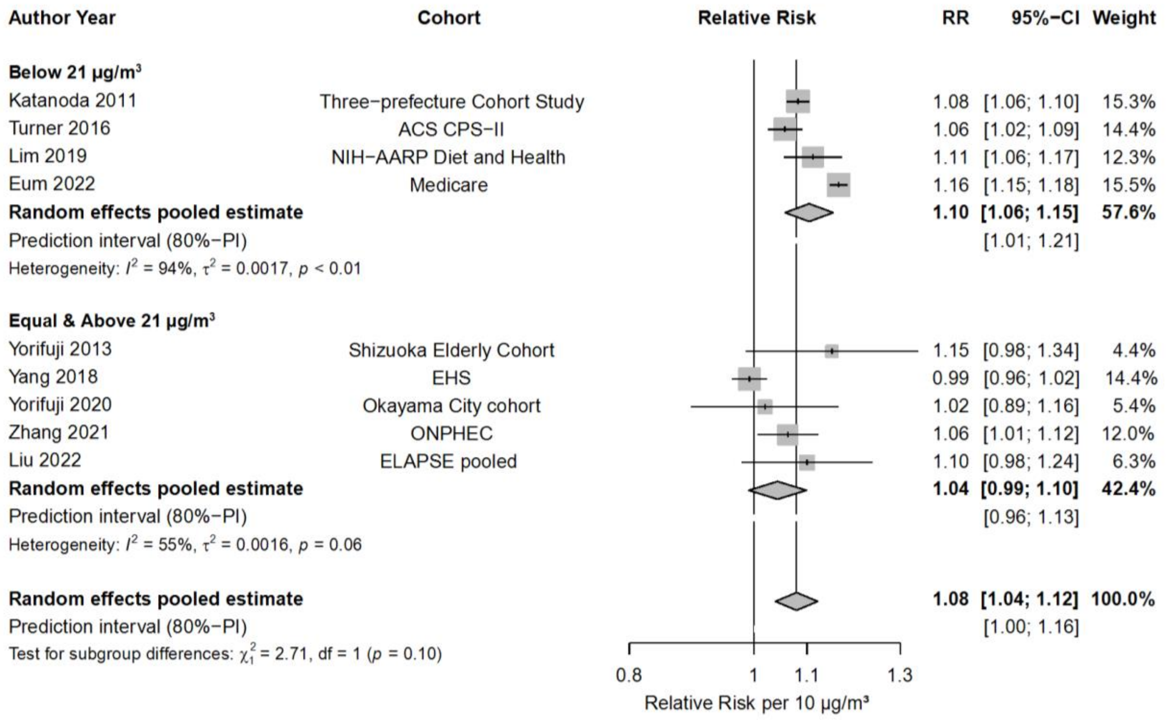

Figure S28. Forest plot of 20 studies on risk of lung cancer mortality associated with 10μg/m^3 increase in NO_2 (Global, 2023-2024).

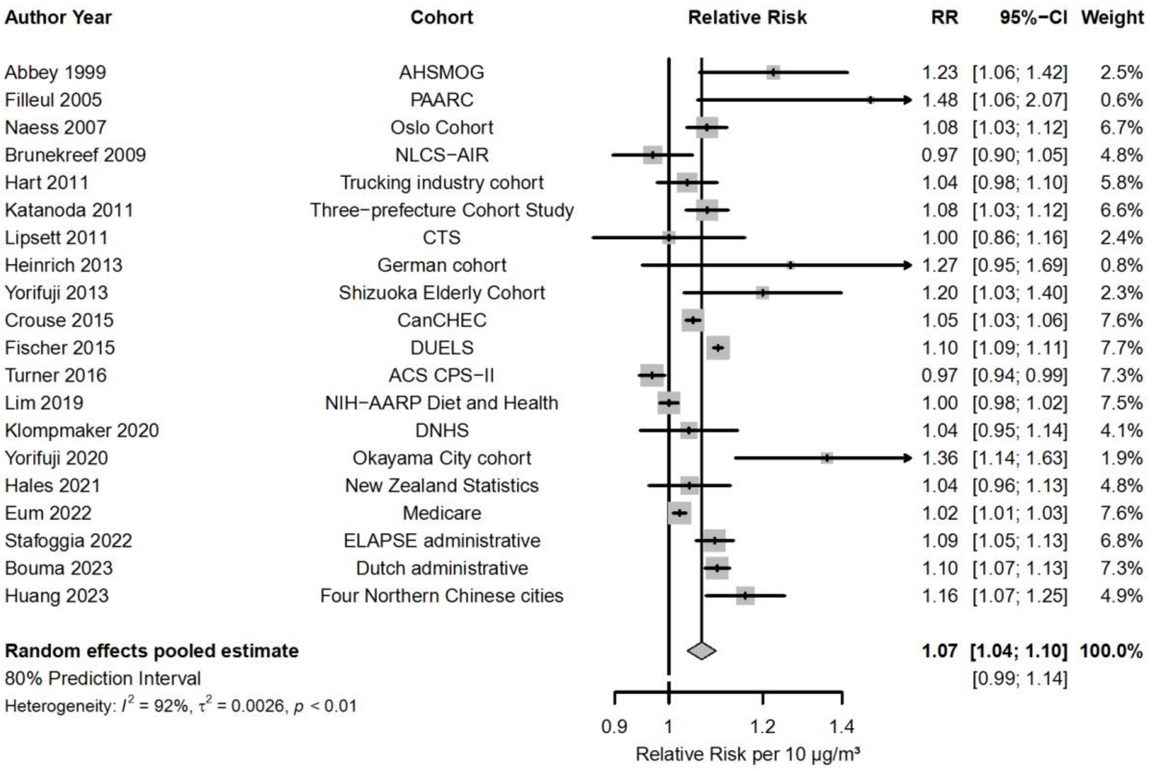

.

Figure S29. Funnel plot with Egger's test p-value for the association lung cancer mortality and 10μg/m3 increase in NO_2 (Global, 2023-2024).

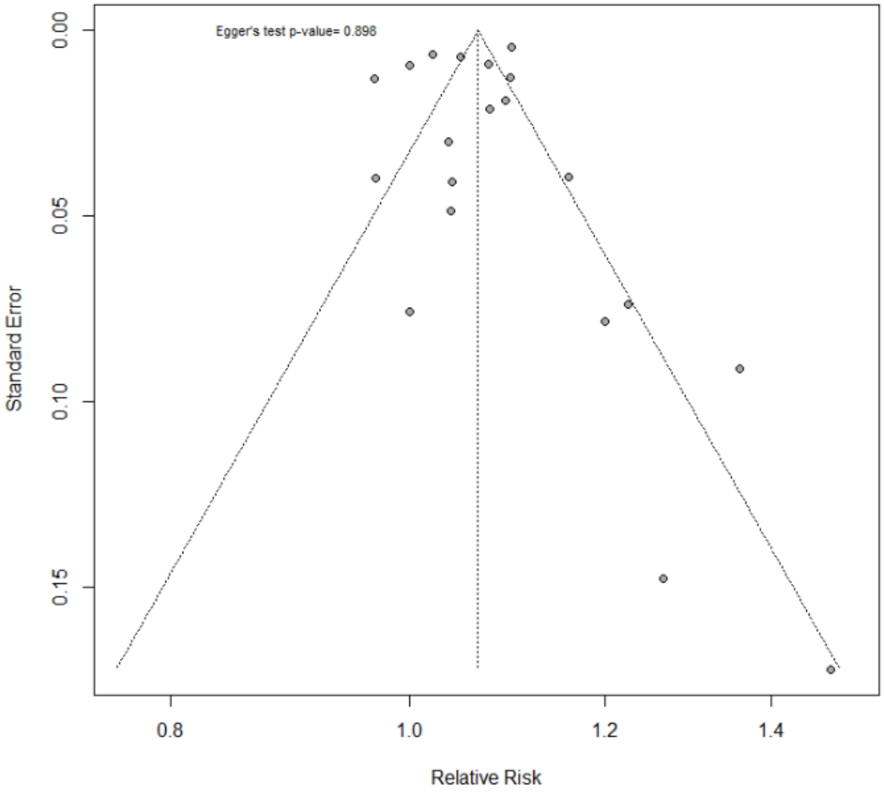

Figure S30. Forest plot for the relative risk (RR) in lung cancer mortality associated with 10μg/m^3 increase in NO_2 by WHO region (Global, 2023-2024).

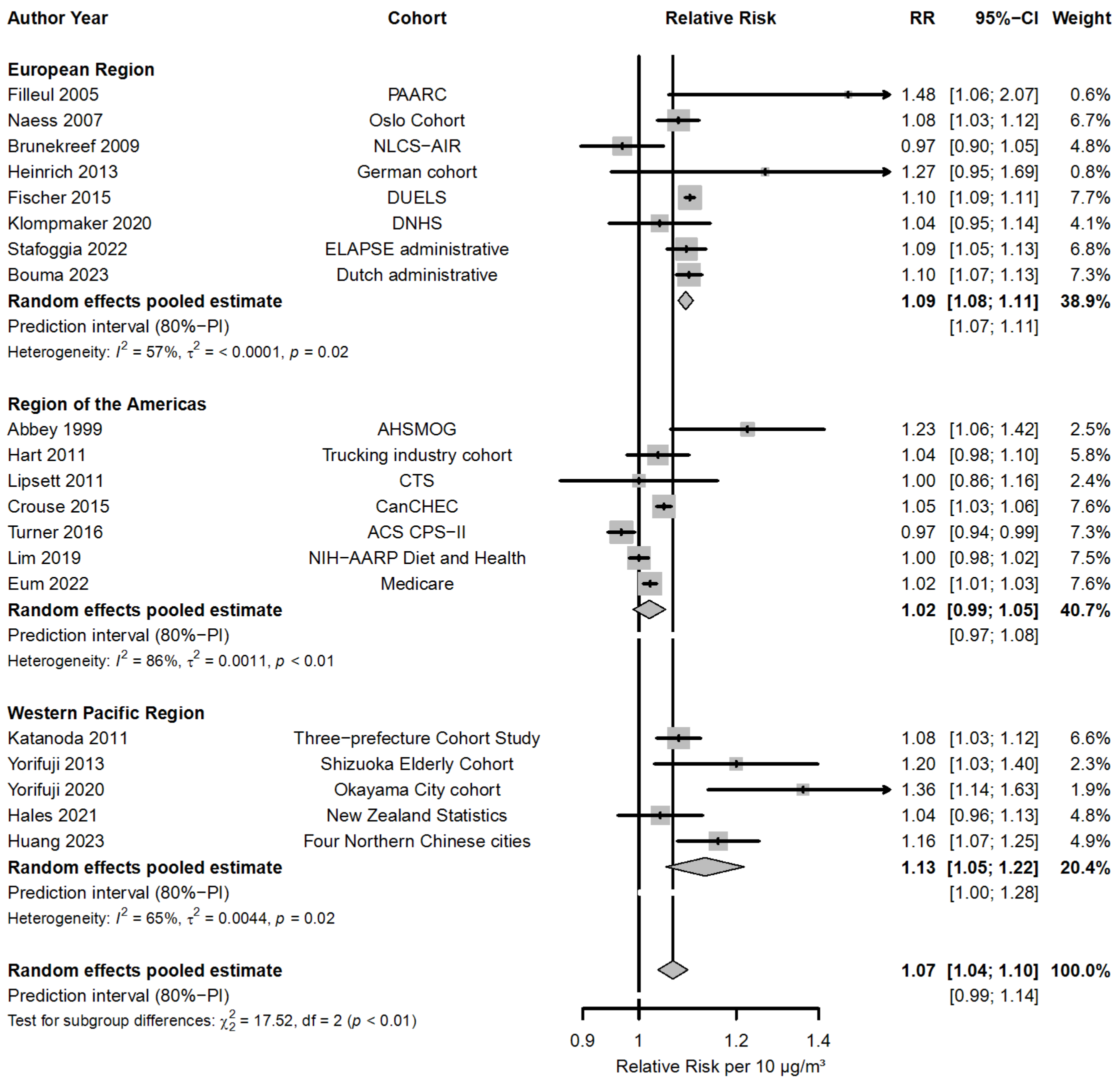

Figure S31. Forest plot for the relative risk (RR) in lung cancer mortality associated with 10μg/m^3 increase in NO_2 by RoB assessment in the confounding domain (Global, 2023-2024).

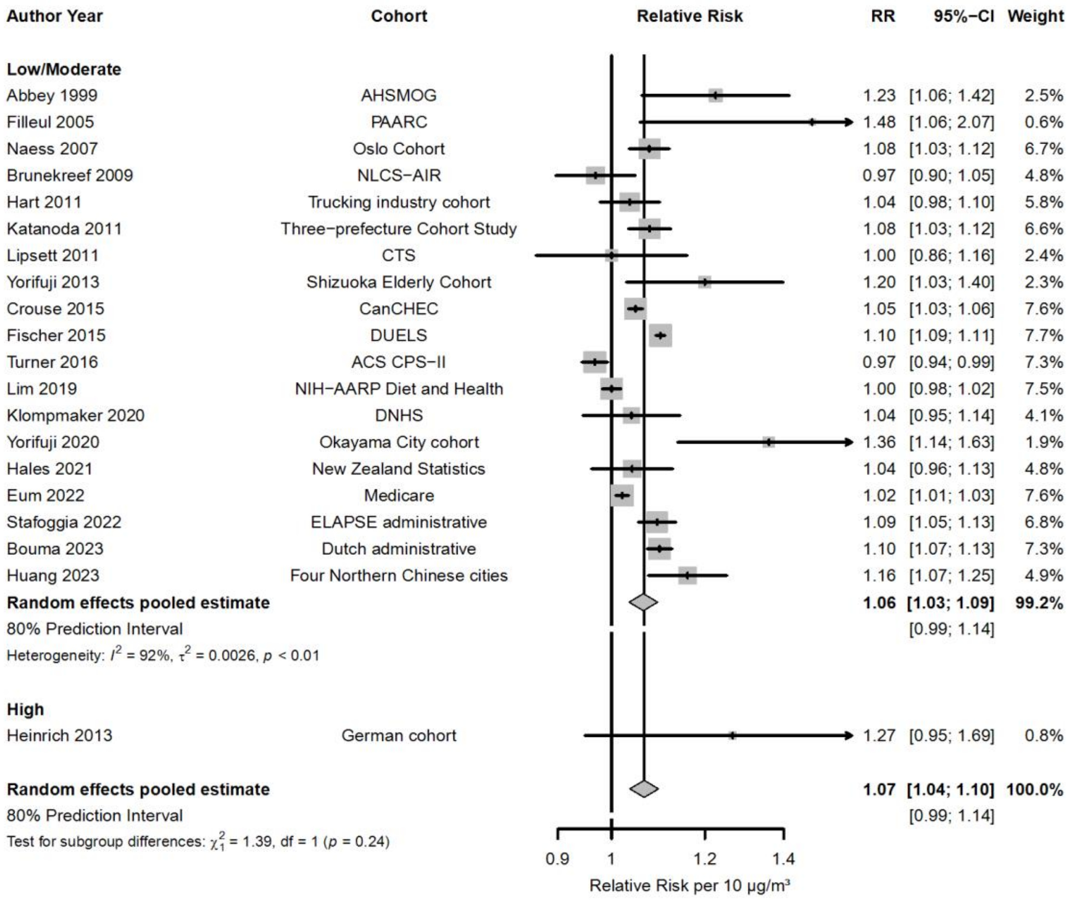

Table S3. GRADE assessment for long-term exposure to NO_2 and mortality outcomes (Global, 2023-2024).

**Mortality Outcome**	**Limitations**	**Indirectness**	**Inconsistency**	**Imprecision**	**Publication Bias**	**Large Effect Size**	**Confounding**	**Concentration Response**	**GRADE**
All-cause	0	0	-1	0	0	0	0	+1	MODERATE
Circulatory	0	0	-1	0	0	0	0	+1	MODERATE
IHD	0	0	-1	0	0	0	0	+1	MODERATE
Cerebrovascular	0	0	-1	0	0	0	0	0	LOW
Respiratory	0	0	-1	0	0	0	0	+1	MODERATE
COPD	0	0	0	0	0	0	0	+1	HIGH
ALRI	0	0	0	0	0	0	0	+1	HIGH
Lung Cancer	0	0	-1	0	0	0	0	+1	MODERATE

0=No downgrade/upgrade, -1=Downgrade one level, +1=Upgrade one level

Table S4. GRADE assessment – NO_2 and all-cause mortality (Global, 2023-2024).

**Domain**	**Rationale**	**Down/Up Grade**
Limitation in studies	N=34 included studies. Risk of bias moderate because although not all studies adjusted for all confounders, exclusion of high risk of bias studies did not reduce the summary RR	No downgrading
Indirectness	All studies included the desired population, exposures and outcomes	No downgrading
Inconsistency	The 80% prediction interval included 1 & > twice CI. High level of heterogeneity. Heterogeneity partly attributed to higher, less precise, risks reported in Western Pacific and lower risk reported in 2 studies with high risk of bias in confounding domain.	Downgrade one level
Imprecision	The number of person years in the included studies was greater than 940 000.	No downgrading
Publication Bias	Funnel plot asymmetry linked to heterogeneity as 32 out of 34 studies reported >1 and asymmetry was not restricted in small studies	No downgrading
Large Effect Size	Summary RR = 1.05. Insufficient information on unmeasured potential confounders available.	No upgrading
Confounding	Confounding direction unknown but precision may be affected.	No upgrading
Concentration-response	A linear dose–response relationship was assumed in all studies. 16 studies investigated the shape of the concentration-response relationship indicating linear or supra linear shapes. 95% CI for linear RR excluded 1.	Upgrade one level
**GRADE conclusion**	Downgrade one level and upgrade one level	MODERATE CERTAINTY. Evidence for mean RR unadjusted for co-pollutants is 1.05 per 10 μg/m^3

Table S5. GRADE assessment – NO_2 and circulatory mortality (Global, 2023-2024).

**Domain**	**Rationale**	**Down/Up Grade**
Limitation in studies	N= 28 included studies. Risk of bias moderate because although not all studies adjusted for all confounders, exclusion of high risk of bias studies increased the summary RR.	No downgrading
Indirectness	All studies included the desired population, exposures and outcomes	No downgrading
Inconsistency	The 80% prediction interval included 1 & > twice CI. High level of heterogeneity partly explained by regional differences.	Downgrade one level
Imprecision	The number of person years in the included studies was greater than 940 000.	No downgrading
Publication Bias	According to the funnel plot and Egger's test, there were no sign of publication bias/funnel plot asymmetry.	No downgrading
Large Effect Size	Summary RR = 1.05. Insufficient information on unmeasured potential confounders available.	No upgrading
Confounding	Confounding direction unknown but precision may be affected.	No upgrading
Concentration-response	A linear dose–response relationship was assumed in all studies. 15 studies investigated the shape of the concentration-response relationship most supporting linear or supra-linear associations. All but one of them indicted increase in risk with increased levels. 95% CI for linear RR excluded 1.	Upgrade one level
**GRADE conclusion**	Downgrade one level and upgrade one level	MODERATE CERTAINTY. Evidence for mean RR unadjusted for co-pollutants is 1.05 per 10 $\mu g/m^3$

Table S6. GRADE assessment – NO_2 and IHD mortality (Global, 2023-2024).

**Domain**	**Rationale**	**Down/Up Grade**
Limitation in studies	N= 20 included studies. Risk of bias for all rated as low or moderate.	No downgrading
Indirectness	All studies included the desired population, exposures and outcomes	No downgrading
Inconsistency	The 80% prediction interval included 1 & > twice CI. High level of heterogeneity not explained by regional differences or NO_2 levels.	Downgrade one level
Imprecision	The number of person years in the included studies was greater than 940 000.	No downgrading
Publication Bias	According to the funnel plot there were no sign of small studies bias.	No downgrading
Large Effect Size	Summary RR = 1.05. Insufficient information on unmeasured potential confounders available.	No upgrading
Confounding	Confounding direction unknown but precision may be affected.	No upgrading
Concentration-response	A linear dose–response relationship was assumed in all studies. 5 studies investigated the shape of the concentration-response relationship with no evidence to suggest non-linear and 4 indicated increased risk with increased levels. 95% CI for linear RR excluded 1.	Upgrade one level
**GRADE conclusion**	Downgrade one level and upgrade one level	MODERATE CERTAINNTY. Evidence for mean RR unadjusted for co-pollutants is 1.05 per 10 $\mu g/m^3$

Table S7. GRADE assessment – NO_2 and cerebrovascular mortality (Global, 2023-2024).

**Domain**	**Rationale**	**Down/Up Grade**
Limitation in studies	N=20 included studies. Risk of bias moderate because although not all studies adjusted for all confounders, exclusion of one high risk of bias study reduced the summary of RR but increased its precision and significance.	No downgrading
Indirectness	All studies included the desired population, exposures and outcomes	No downgrading
Inconsistency	The 80% prediction interval included 1 & > twice CI. High level of heterogeneity not explained by regional differences or NO_2 levels.	Downgrade one level
Imprecision	The number of person years in the included studies was greater than 940 000.	No downgrading
Publication Bias	According to the funnel plot there were no sign of small study bias.	No downgrading
Large Effect Size	Summary RR = 1.08. Insufficient information on unmeasured potential confounders available.	No upgrading
Confounding	Confounding direction unknown but precision may be affected.	No upgrading
Concentration-response	A linear dose–response relationship was assumed in all studies. 5 studies investigated the shape of the concentration-response relationship indicating increased risk with increased levels. 95% CI for linear RR included 1.	No upgrading
**GRADE conclusion**	Downgrade one level and upgrade one level	LOW CERTAINTY. Evidence for mean RR unadjusted for co-pollutants is 1.08 per 10 $\mu g/m^3$

Table S8. GRADE assessment – NO_2 and respiratory mortality (Global, 2023-2024).

**Domain**	**Rationale**	**Down/Up Grade**
Limitation in studies	N=25 included studies. Risk of bias moderate because although not all studies adjusted for all confounders, exclusion of high risk of bias studies did not reduce the summary RR.	No downgrading
Indirectness	All studies included the desired population, exposures and outcomes	No downgrading
Inconsistency	The 80% prediction interval included 1 & > twice CI. High level of heterogeneity partly explained by NO_2 levels.	Downgrade one level
Imprecision	The number of person years in the included studies was greater than 940 000.	No downgrading
Publication Bias	According to the funnel plot and Egger's test there were no sign of publication bias/funnel plot asymmetry.	No downgrading
Large Effect Size	Summary RR = 1.05. Insufficient information on unmeasured potential confounders available.	No upgrading
Confounding	Confounding direction unknown but precision may be affected.	No upgrading
Concentration-response	A linear dose–response relationship was assumed in all studies. 8 studies investigated the shape of the concentration-response relationship suggesting linear or supra-linear shapes and 6 out of 8 increased risk with increased levels. 95% CI for linear RR excluded 1.	Upgrade one level
**GRADE conclusion**	Downgrade one level and upgrade one level	MODERATE CERTAINTY. Evidence for mean RR unadjusted for co-pollutants is 1.05 per 10 $\mu g/m^3$

Table S9. GRADE assessment – NO_2 and COPD mortality (Global, 2023-2024).

**Domain**	**Rationale**	**Down/Up Grade**
Limitation in studies	N=15 included studies. Risk of bias moderate because although not all studies adjusted for all confounders, exclusion of high risk of bias studies did not affect the summary RR.	No downgrading
Indirectness	All studies included the desired population, exposures and outcomes	No downgrading
Inconsistency	12 out of the 14 estimates were greater than 1and heterogeneity greatly reduced when excluding high RoB studies.	No downgrading
Imprecision	The number of person years in the included studies was greater than 940 000.	No downgrading
Publication Bias	According to the funnel plot and Egger's test there were no sign of publication bias/funnel plot asymmetry.	No downgrading
Large Effect Size	Summary RR = 1.04. Insufficient information on unmeasured potential confounders available.	No upgrading
Confounding	Confounding direction unknown but precision may be affected.	No upgrading
Concentration-response	A linear dose–response relationship was assumed in all studies. 4 studies investigated the shape of the concentration-response relationship with 3 indicating increased risk with increased levels. 95% CI for linear RR excluded 1.	Upgrade one level
**GRADE conclusion**	Downgrade one level and upgrade one level	HIGH CERTAINTY. Evidence for mean RR unadjusted for co-pollutants is 1.04 per 10 $\mu g/m^3$

Table S10. GRADE assessment – NO_2 and ALRI mortality (Global, 2023-2024).

**Domain**	**Rationale**	**Down/Up Grade**
Limitation in studies	N=9 included studies. Risk of bias moderate because although not all studies adjusted for all confounders, with no high risk of bias studies.	No downgrading
Indirectness	All studies included the desired population, exposures and outcomes	No downgrading
Inconsistency	The 80% prediction interval did not include 1 & just twice CI. High level of heterogeneity in general population partly explained by region and NO_2 levels. All study specific >1 and the pooled RR is not driven by smaller studies.	No downgrade
Imprecision	The number of person years in the included studies was greater than 940 000.	No downgrading
Publication Bias	According to the funnel plot and Egger's test (P < 0.1), there were no sign of publication bias/funnel plot asymmetry.	No downgrading
Large Effect Size	Summary RR = 1.08. Insufficient information on unmeasured potential confounders available.	No upgrading
Confounding	Confounding direction unknown but precision may be affected.	No upgrading
Concentration-response	A linear dose–response relationship was assumed in all studies. 2 studies investigated the shape of the concentration-response relationship with no evidence to suggest non-linear and increased risk with increased level. 95% CI for linear RR excluded 1.	Upgrade one level
**GRADE conclusion**	Downgrade one level and upgrade one level	HIGH CERTAINTY. Evidence for mean RR unadjusted for co-pollutants is 1.08 per 10 $\mu g/m^3$

Table S11. GRADE assessment – NO_2 and lung cancer mortality (Global, 2023-2024).

**Domain**	**Rationale**	**Down/Up Grade**
Limitation in studies	N=20 included studies. Risk of bias moderate because although not all studies adjusted for all confounders, exclusion of high risk of bias studies did not reduce the summary RR.	No downgrading
Indirectness	All studies included the desired population, exposures and outcomes	No downgrading
Inconsistency	The 80% prediction interval included 1 & > twice CI. High level of heterogeneity partly explained by higher RR in the Western pacific region and NO_2 levels.	Downgrade one level
Imprecision	The number of person years in the included studies was greater than 940 000.	No downgrading
Publication Bias	According to the funnel plot there were no sign of small study bias.	No downgrading
Large Effect Size	Summary RR = 1.07. Insufficient information on unmeasured potential confounders available.	No upgrading
Confounding	Confounding direction unknown but precision may be affected.	No upgrading
Concentration-response	A linear dose–response relationship was assumed in all studies. 5 studies investigated the shape of the concentration-response relationship indicating increased risk with increased levels. 95% CI for linear RR excluded 1.	Upgrade one level
**GRADE conclusion**	Downgrade one level and upgrade one level	MODERATE CERTAINTY. Evidence for mean RR unadjusted for co-pollutants is 1.07 per 10 μg/m^3

Figure S32. Forest plot of 9 studies on the risk of all-cause mortality associated with 10μg/m^3 increase in long-term exposure to annual O_3 (Global, 2023-2024).

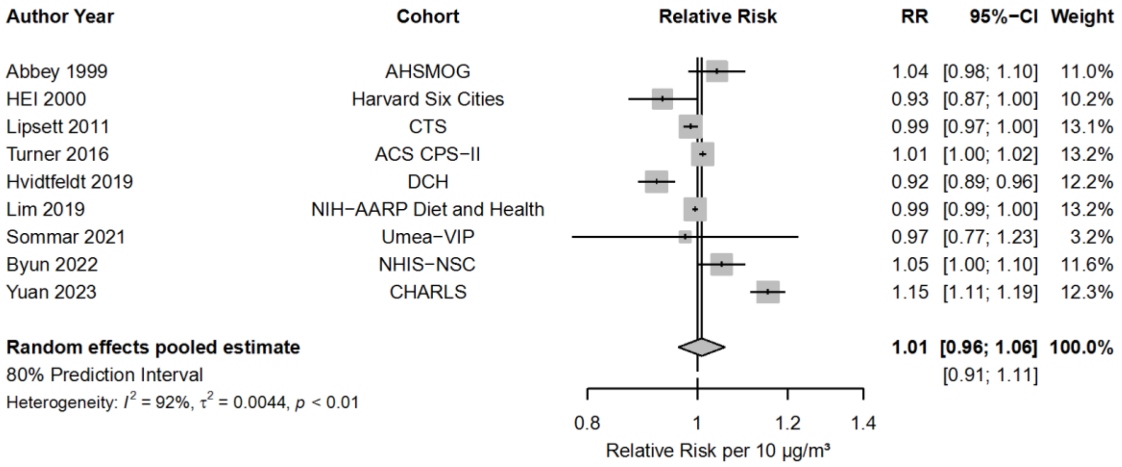

Figure S33. Funnel plot of the association between all-cause mortality and 10μg/m^3 increase in annual O_3 (Global, 2023-2024).

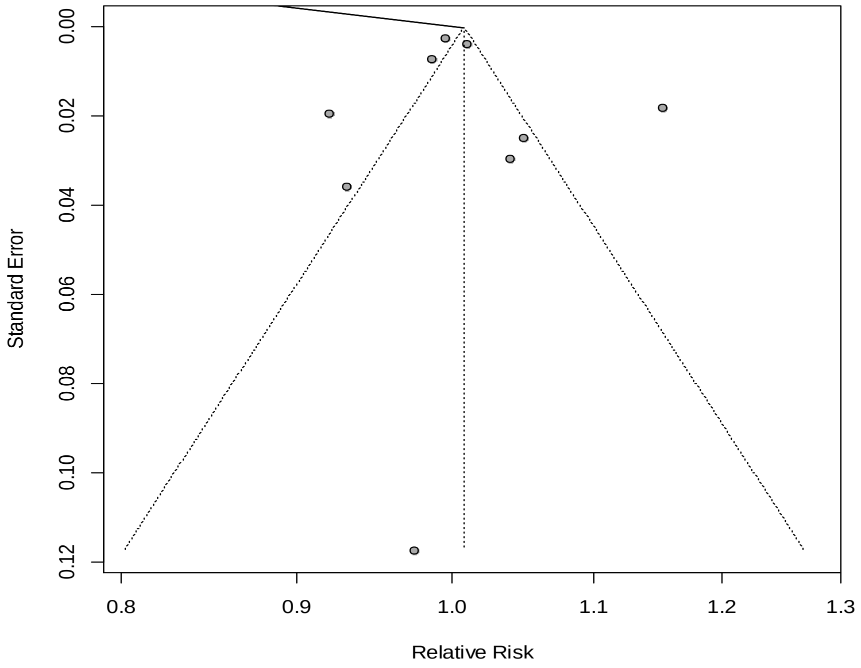

Figure S34. Forest plot for the relative risk (RR) in all-cause mortality associated with 10µg/m[3] increase in annual O_3 by WHO region (Global, 2023-2024).

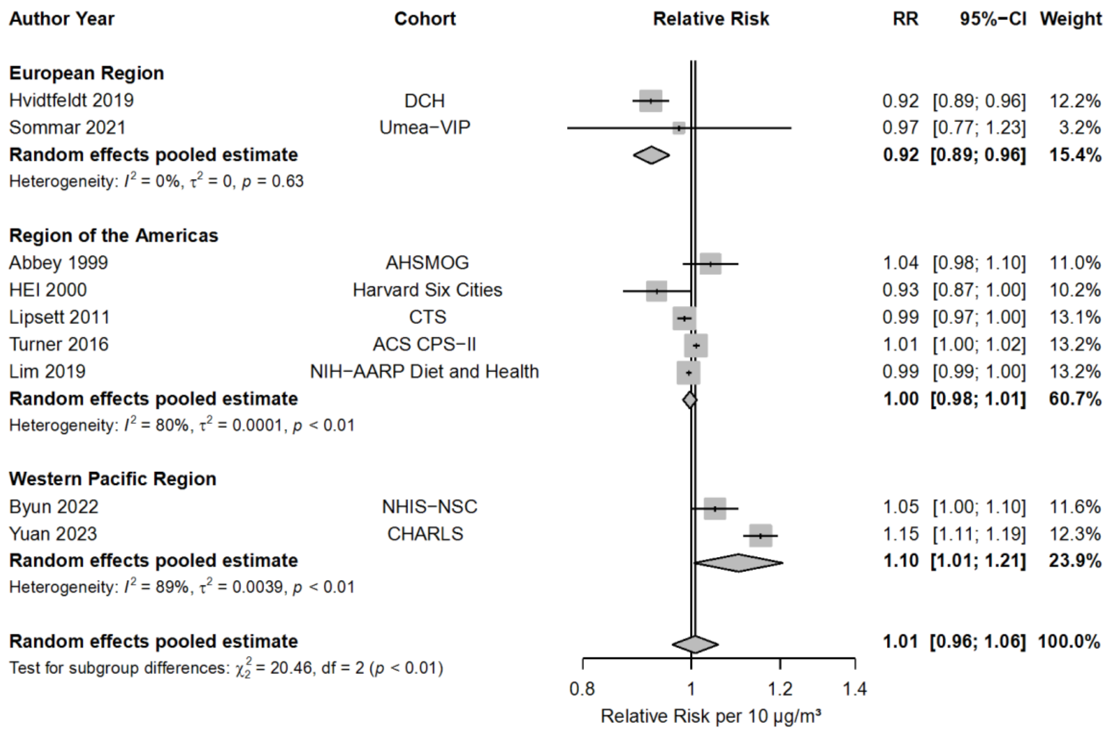

Figure S35. Forest plot of 6 studies on the risk of respiratory disease mortality associated with 10μg/m^3 increase in annual O_3 (Global, 2023-2024).

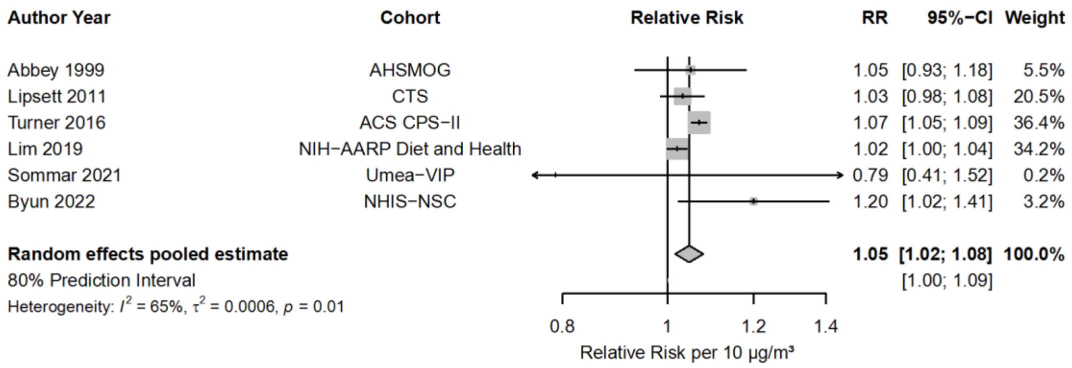

Figure S36. Funnel plot of the association respiratory disease mortality and 10μg/m^3 increase in annual O_3 (Global, 2023-2024).

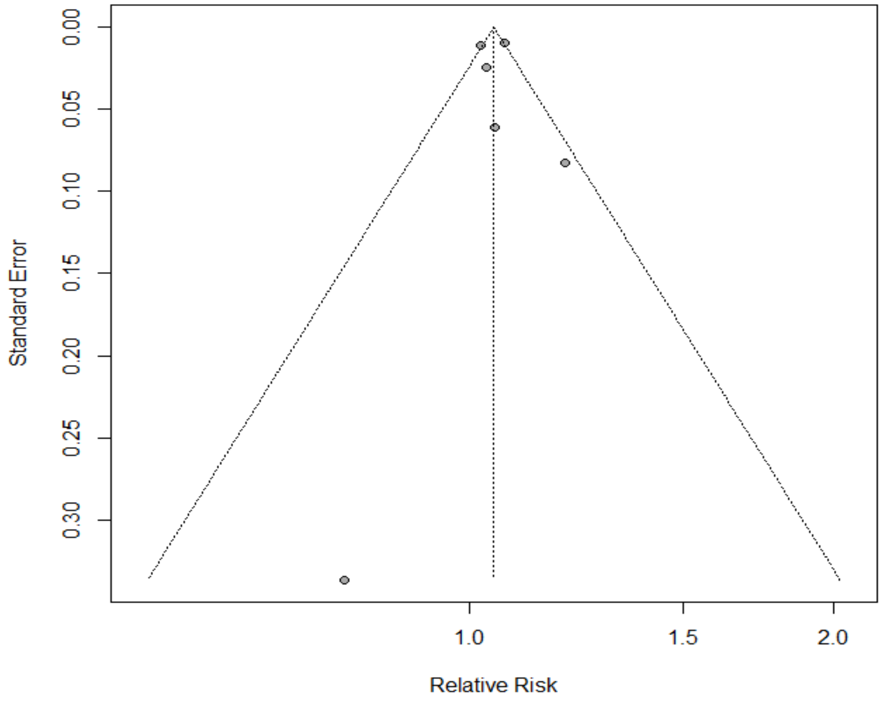

Figure S37. Forest plot for the relative risk (RR) in respiratory disease mortality associated with 10μg/m^3 increase in annual O_3 by WHO region (Global, 2023-2024).

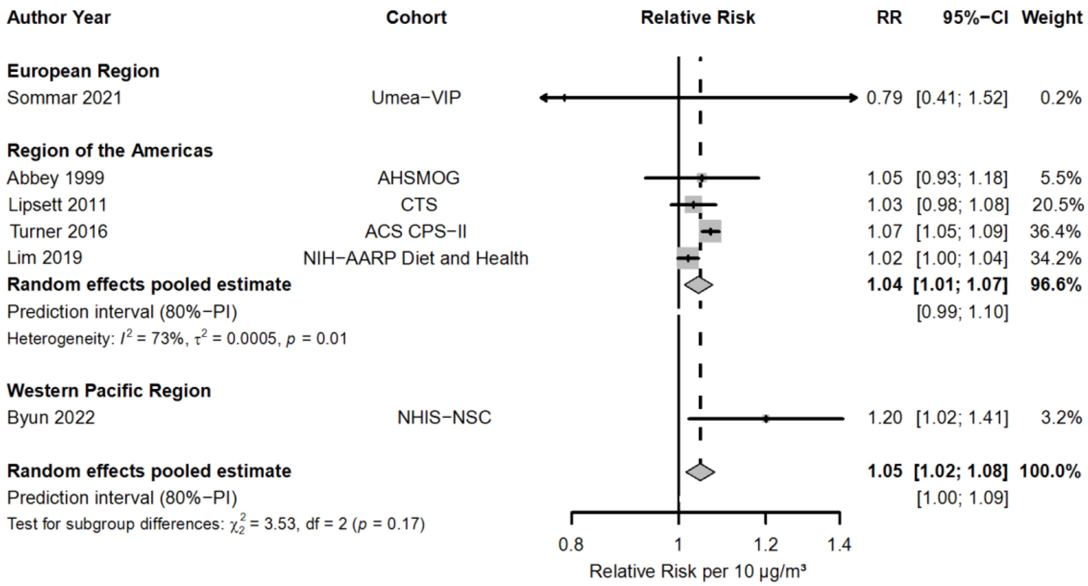

Figure S38. Forest plot for the relative risk (RR) in respiratory mortality associated with 10μg/m^3 increase in annual O_3 by annual O_3 levels (Global, 2023-2024).

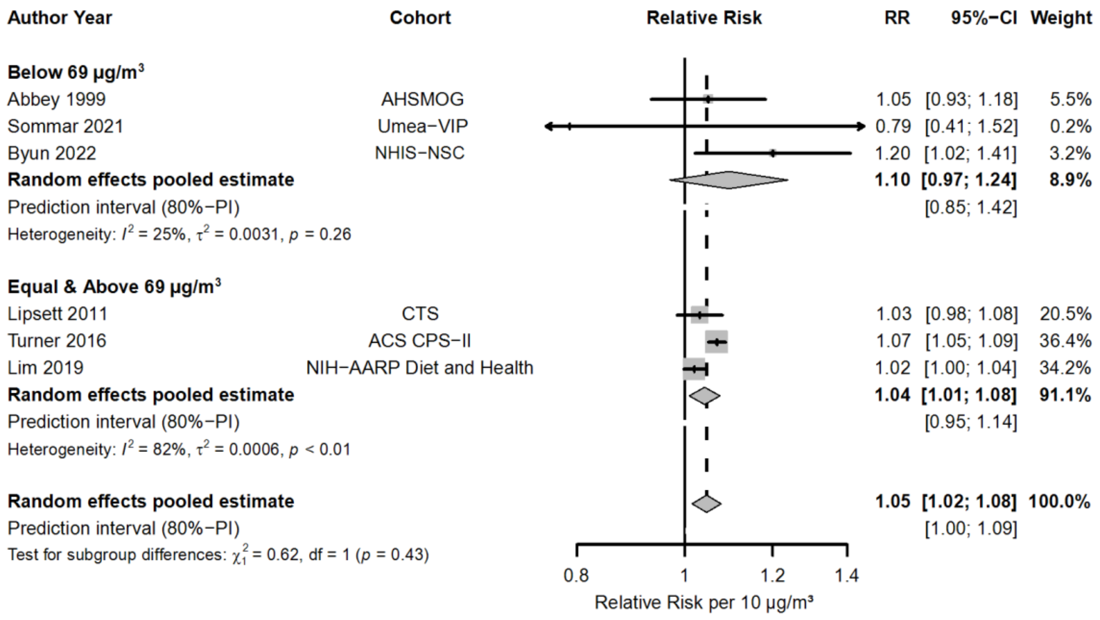

Figure S39 Forest plot of 2 studies on risk of COPD mortality associated with 10µg/m^3 increase in annual O_3 (Global, 2023-2024).

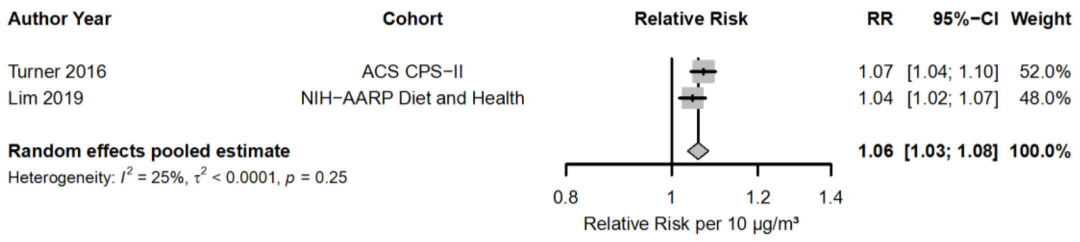

Figure S40. Forest plot of 2 studies on risk of ALRI mortality associated with 10µg/m^3 increase in annual O_3 (Global, 2023-2024).

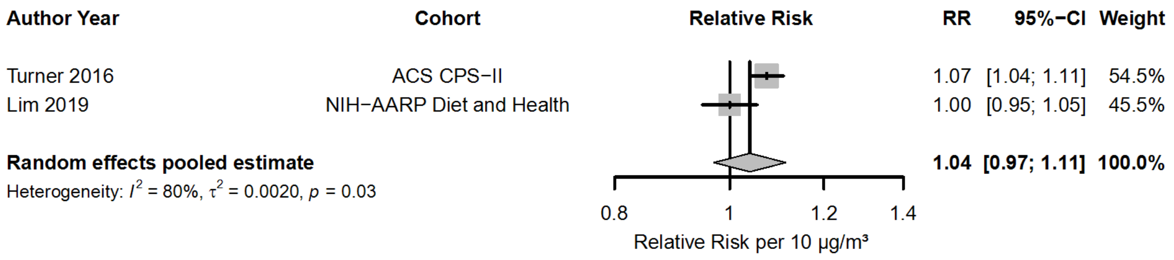

Figure S41 Forest plot of 12 studies on the risk of all-cause mortality associated with 10μg/m^3 increase in peak/warm period O_3 (Global, 2023-2024).

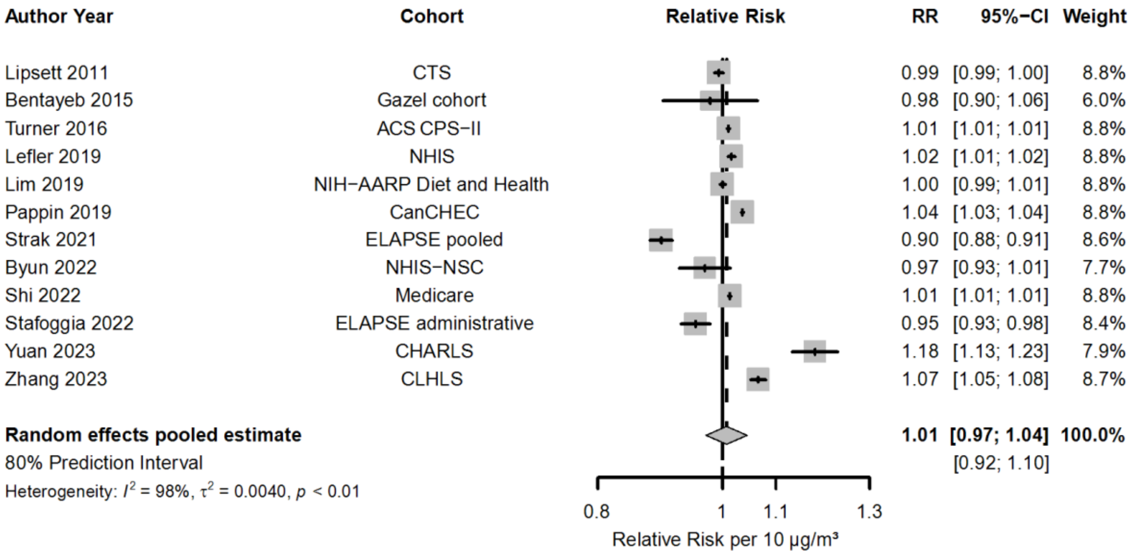

Figure S42. Funnel plot with Egger's test p-value of the association between all-cause mortality and 10μg/m^3 increase in peak/warm period O_3 (Global, 2023-2024).

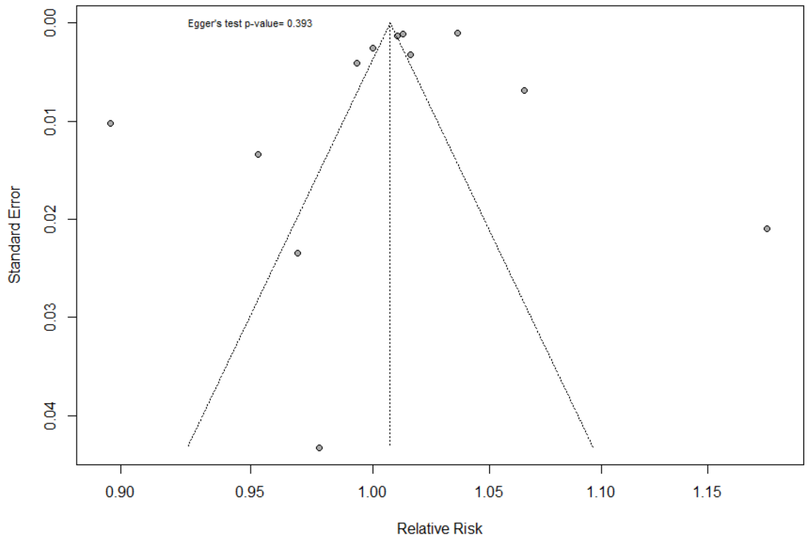

Figure S43. Forest plot for the relative risk (RR) in all-cause mortality associated with 10µg/m^3 increase in peak/warm period O_3 by WHO region (Global, 2023-2024).

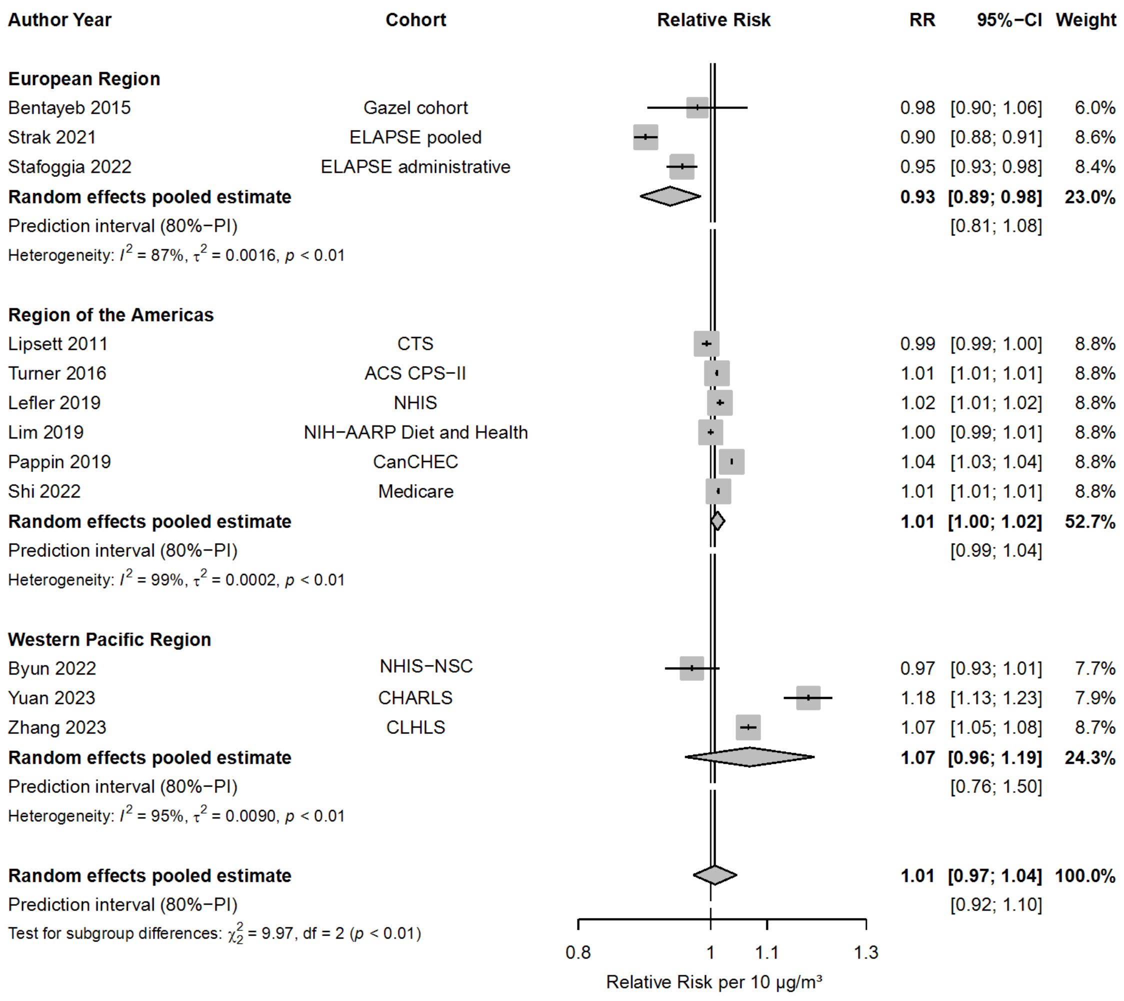

Figure S44. Forest plot for the relative risk (RR) in all-cause mortality associated with 10μg/m^3 increase in peak/warm period O_3 by RoB assessment in the confounding domain (Global, 2023-2024).

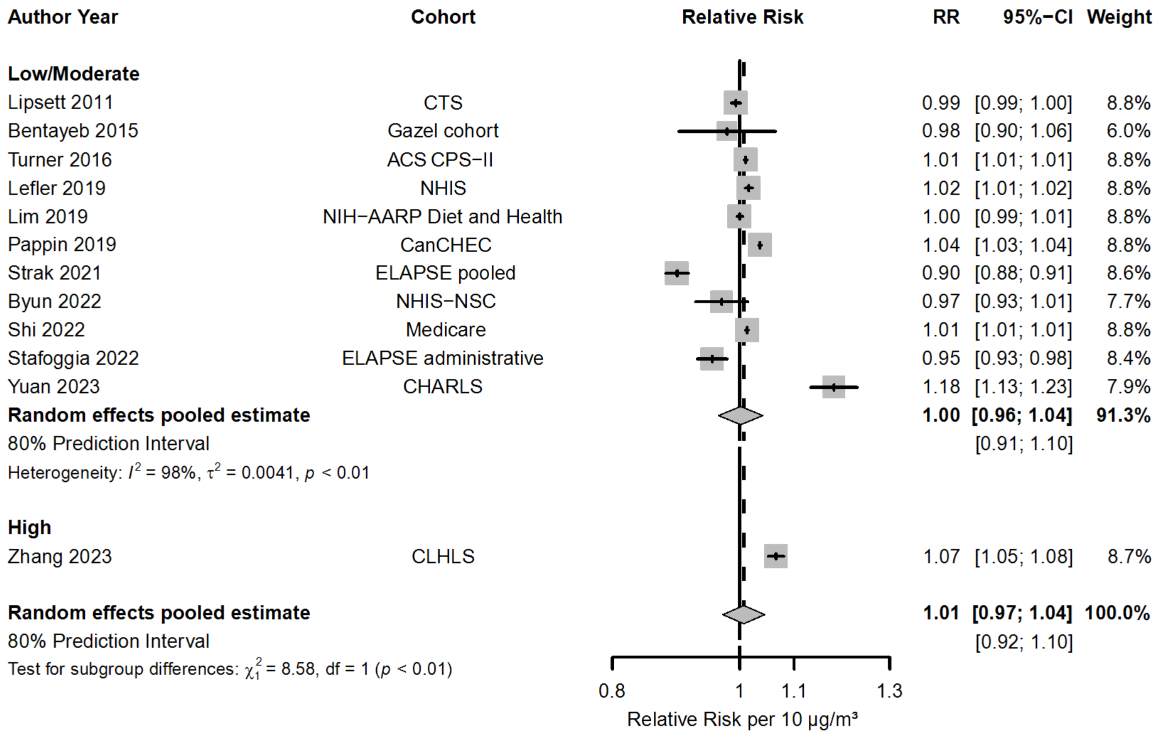

Figure S45. Forest plot of 9 studies on the risk of respiratory disease mortality associated with 10μg/m^3 increase in peak/warm period O_3 (Global, 2023-2024).

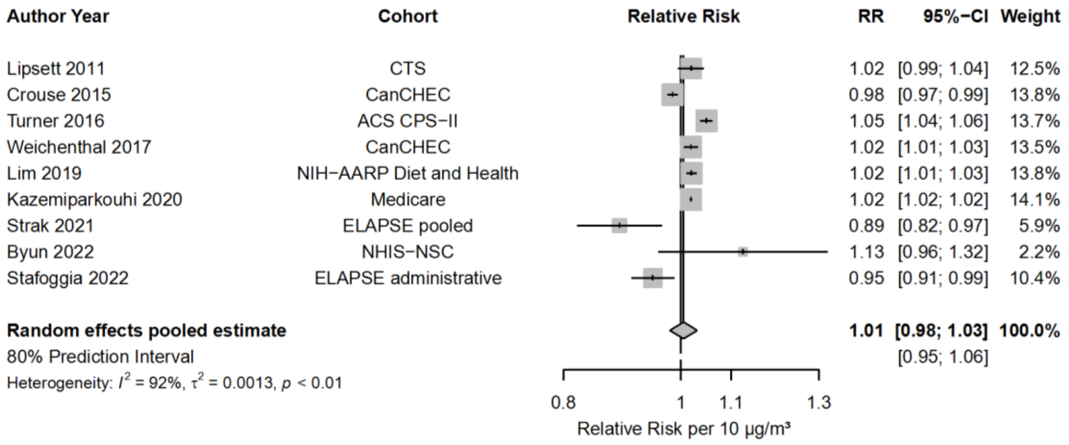

Figure S46. Funnel plot for the association respiratory disease mortality and 10μg/m^3 increase in peak/warm period O_3 (Global, 2023-2024).

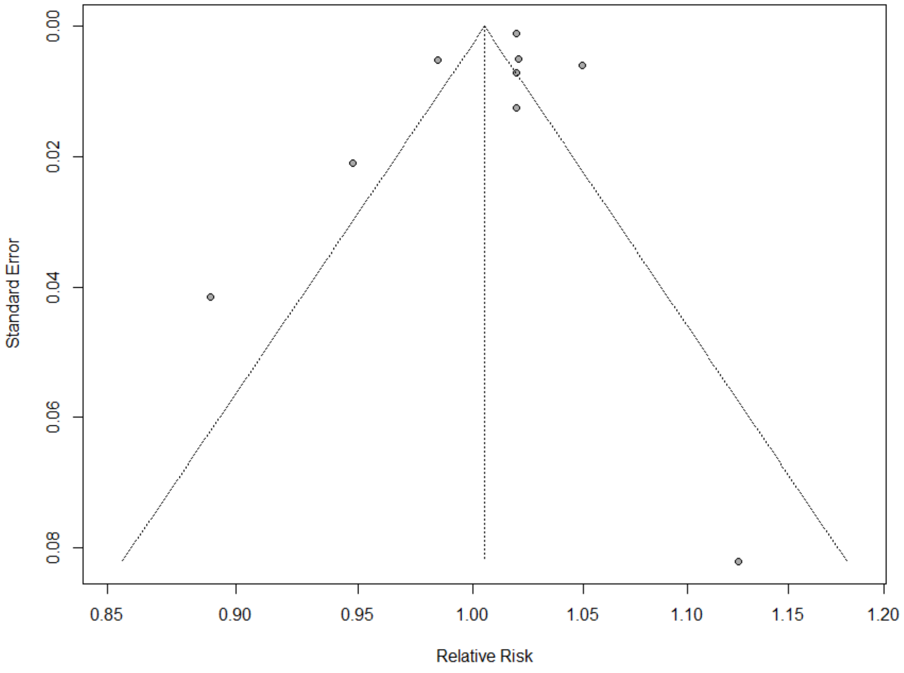

Figure S47. Forest plot for the relative risk (RR) in respiratory disease mortality associated with 10μg/m^3 increase in peak/warm period O_3 by WHO region (Global, 2023-2024).

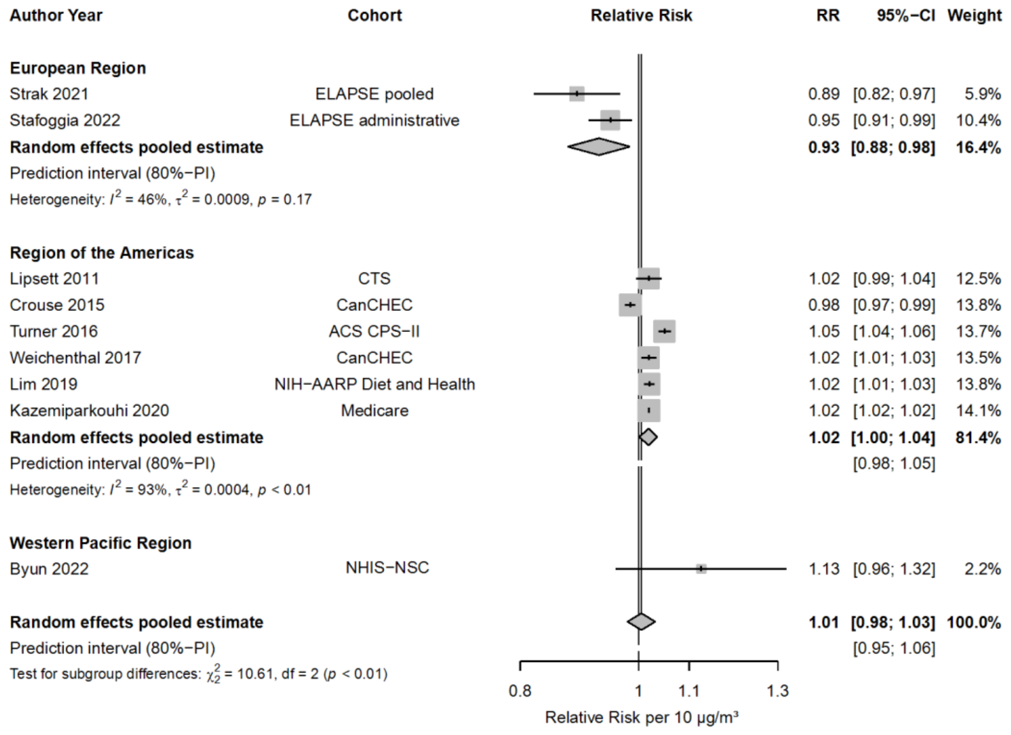

Figure S48. Forest plot for the relative risk (RR) in in respiratory mortality associated with 10µg/m^3 increase in peak/warm period O_3 by RoB assessment in the confounding domain (Global, 2023-2024).

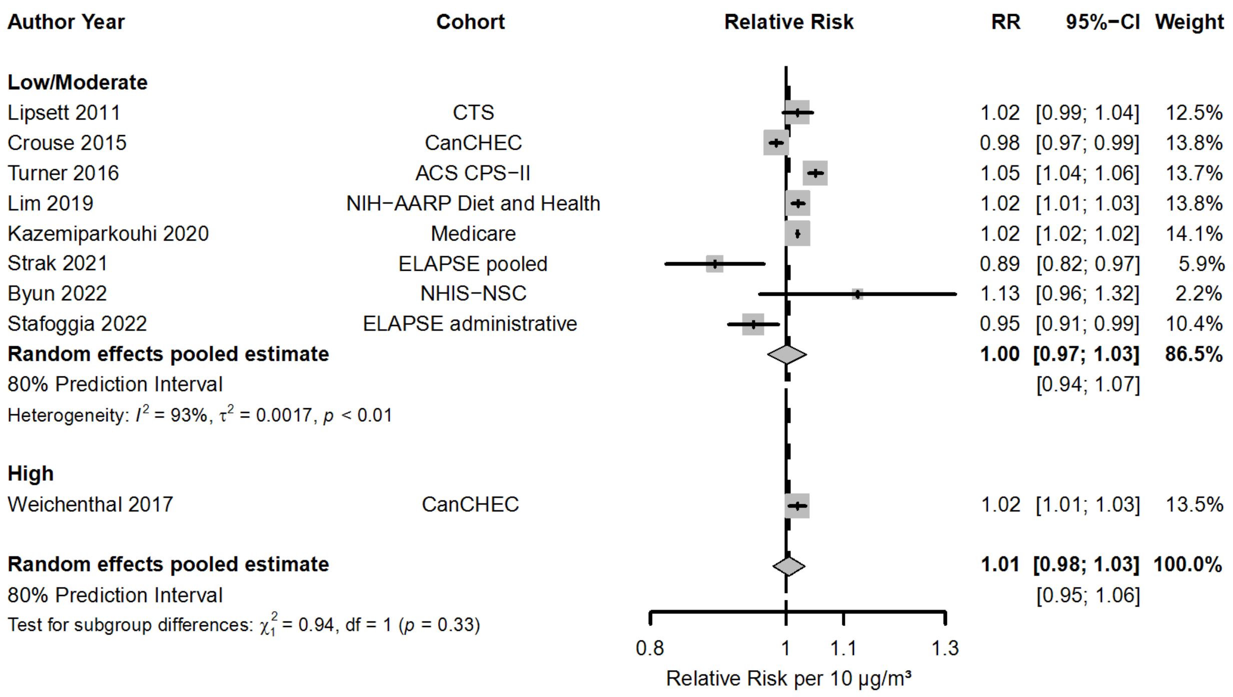

.

Figure S49. Forest plot for the relative risk (RR) in in respiratory mortality associated with 10μg/m^3 increase in peak/warm period O_3 by peak/warm O_3 levels (Global, 2023-2024).

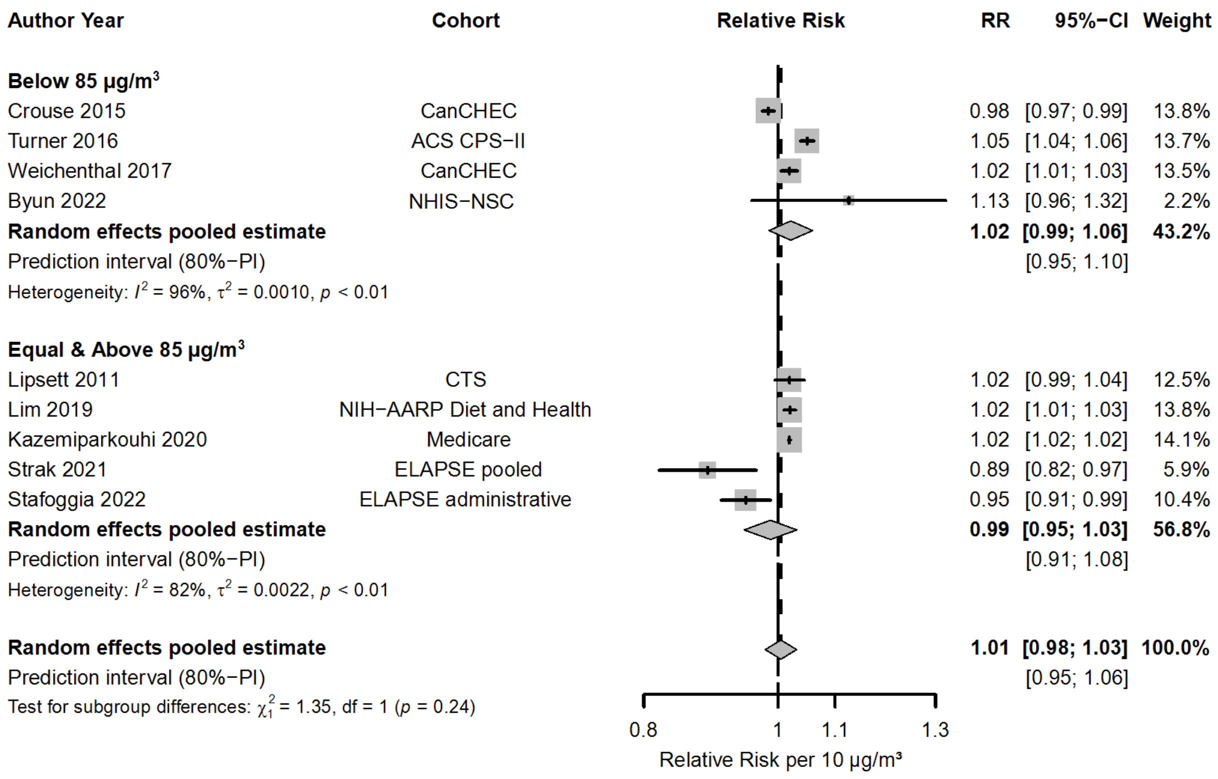

Figure S50. Forest plot of 7 studies on the risk of COPD mortality associated with 10μg/m^3 increase in peak/warm period O_3 (Global, 2023-2024).

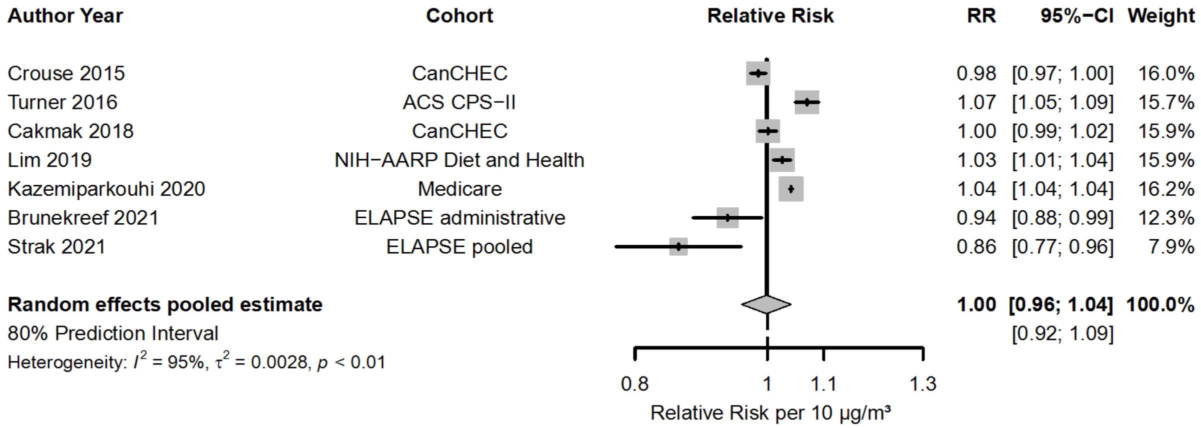

.

Figure S51. Funnel plot for the association COPD mortality and 10μg/m^3 increase in peak/warm period O_3 (Global, 2023-2024).

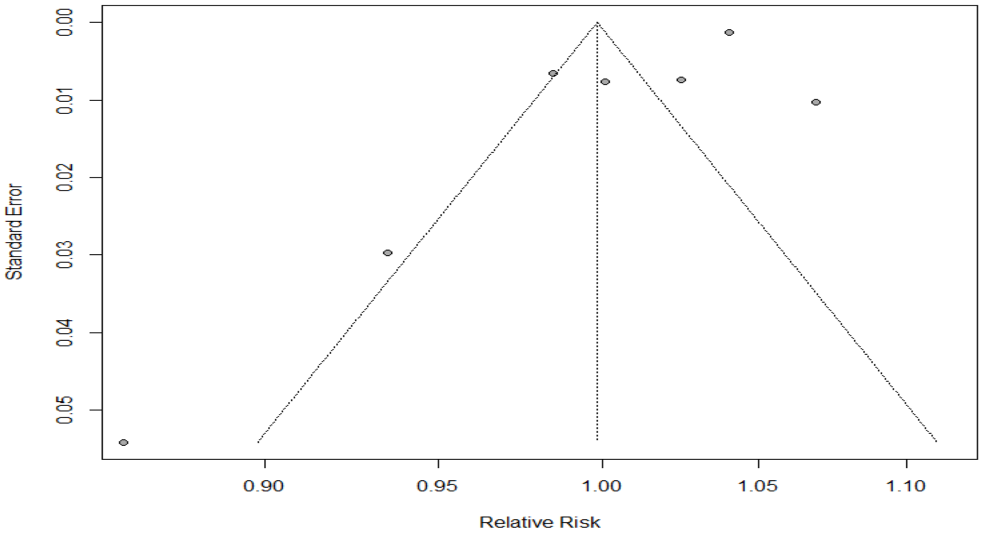

Figure S52. Forest plot for the relative risk (RR) in in COPD mortality associated with 10µg/m^3 increase in peak/warm period O_3 by WHO region (Global, 2023-2024).

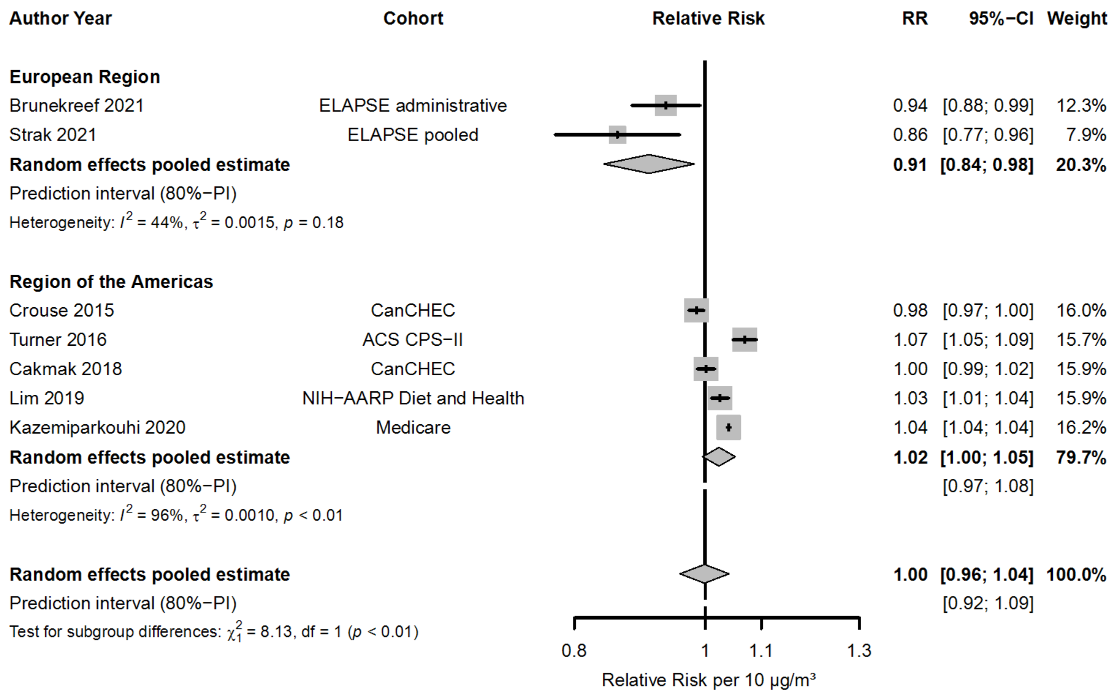

Figure S53. Forest plot for the relative risk (RR) in in COPD mortality associated with 10µg/m^3 increase in peak/warm period O_3 by peak/warm O_3 levels (Global, 2023-2024).

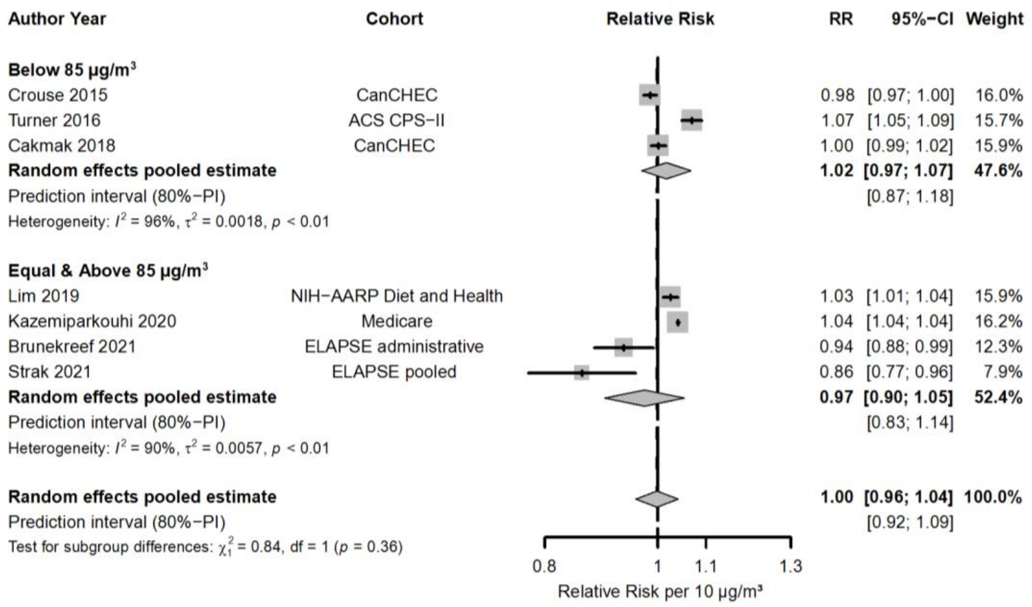

Figure S54. Forest plot of 4 studies on the risk of ALRI mortality associated with 10µg/m^3 increase in peak/warm period O_3 (Global, 2023-2024).

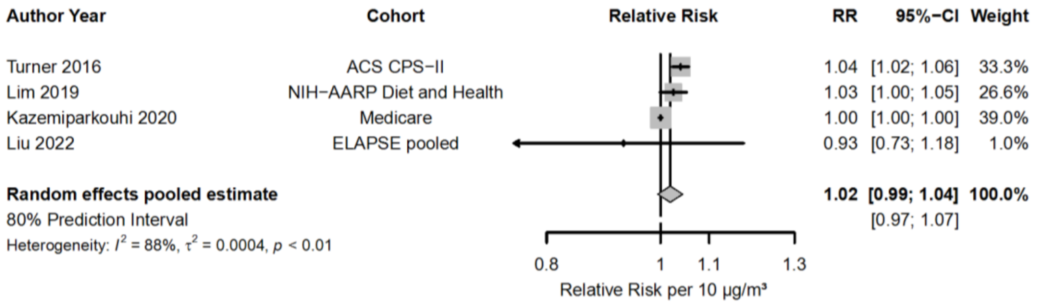

.

Figure S55. Funnel plot for the association between all-cause mortality and 10µg/m^3 increase in annual O_3 (Global, 2023-2024).

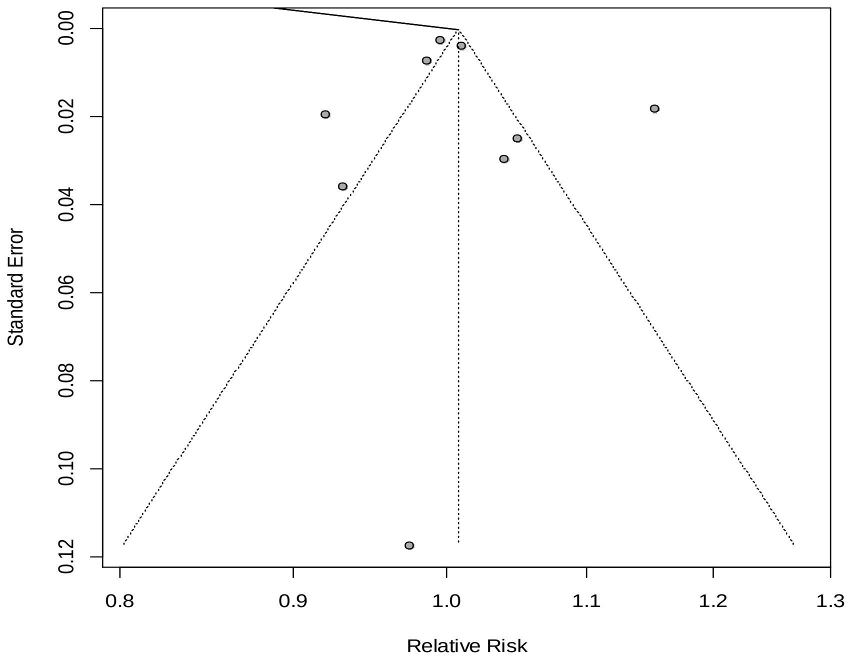

Figure S56. Forest plot for the relative risk (RR) in in ALRI mortality associated with 10µg/m³ increase in peak/warm period O_3 by WHO region (Global, 2023-2024).

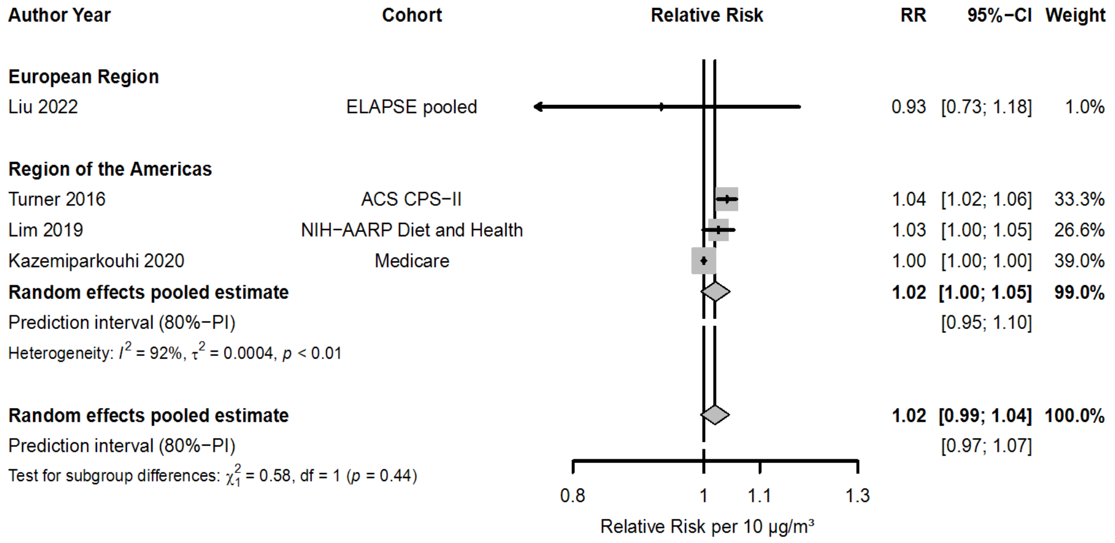

Figure S57. Forest plot for the relative risk (RR) in in ALRI mortality associated with 10µg/m³ increase in peak/warm period O_3 by peak/warm O_3 levels (Global, 2023-2024).

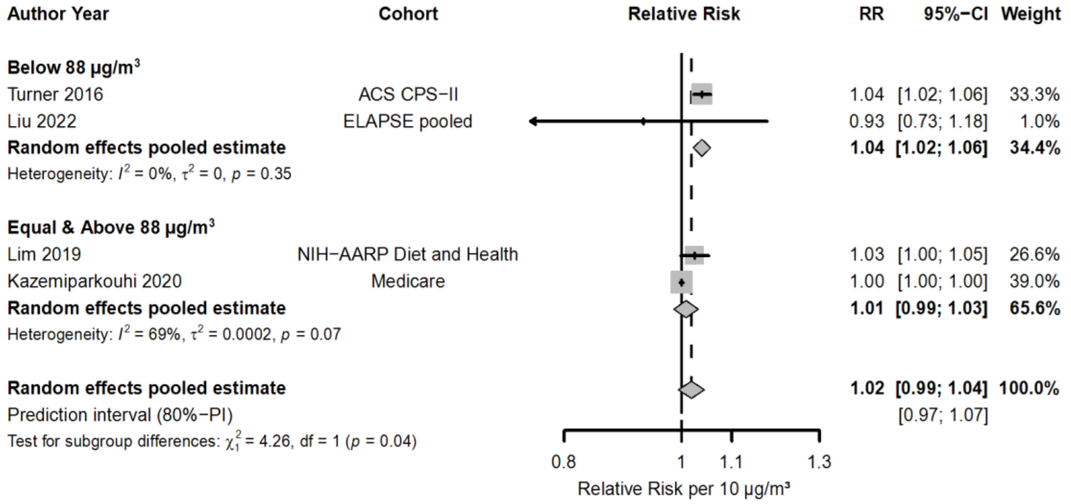

Table S12. GRADE assessment for long-term exposure to a) Annual and b) Peak/Warm season O_3 and mortality outcomes (Global, 2023-2024).

A) Annual O_3

**Mortality Outcome**	**Limitations**	**Indirectness**	**Inconsistency**	**Imprecision**	**Publication Bias**	**Large Effect Size**	**Confounding**	**Concentration Response**	**GRADE**
All-cause	0	0	-1	0	0	0	0	0	LOW
Respiratory	0	0	0	0	0	0	0	+1	HIGH

0=No downgrade/upgrade, -1=Downgrade one level, +1=Upgrade one level

B) Peak/Warm season O_3

**Mortality Outcome**	**Limitations**	**Indirectness**	**Inconsistency**	**Imprecision**	**Publication Bias**	**Large Effect Size**	**Confounding**	**Concentration Response**	**GRADE**
All-cause	0	0	-1	0	0	0	0	0	LOW
Respiratory	0	0	-1	0	0	0	0	+1	LOW
COPD	0	0	-1	0	0	0	0	+1	LOW
ALRI	0	0	-1	0	0	0	0	+1	LOW

0=No downgrade/upgrade, -1=Downgrade one level, +1=Upgrade one level

Table S13. GRADE assessment – O_3 annual and All-cause mortality (Global, 2023-2024).

Domain	Rationale	Down/Up Grade
Limitation in studies	N=9 included studies. Risk of bias low or moderate.	No downgrading
Indirectness	All studies included the desired population, exposures and outcomes	No downgrading
Inconsistency	The 80% prediction interval included 1 & twice the CI. High level of heterogeneity partly attributed to higher, less precise, risks reported in Western Pacific.	Downgrade one level
Imprecision	The number of person years in the included studies was greater than 940 000.	No downgrading
Publication Bias	According to the funnel there were no sign of asymmetry.	No downgrading
Large Effect Size	Summary RR = 1.01. Insufficient information on unmeasured potential confounders available.	No upgrading
Confounding	Confounding direction unknown but precision may be affected.	No upgrading
Concentration-response	A linear dose–response relationship was assumed in all studies. 3 studies investigated the shape of the concentration-response relationship with no evidence to suggest non-linear but indicated decreased risk with increased exposure. CI included 1.	No upgrading
**GRADE conclusion**	Downgrade one level and no upgrade	LOW CERTAINTY. Evidence for mean RR unadjusted for co-pollutants is 1.01 per 10 $\mu g/m^3$

Table S14. GRADE assessment – Annual O_3- Respiratory mortality (Global, 2023-2024).

**Domain**	**Rationale**	**Down/Up Grade**
Limitation in studies	N=6 included studies. Risk of bias moderate although not all studies adjusted for all confounders.	No downgrading
Indirectness	All studies included the desired population, exposures and outcomes	No downgrading
Inconsistency	The 80% prediction interval did not include 1 & was almost the same size as the CI.<br>Heterogeneity partly explained by regional differences.	No downgrading
Imprecision	The number of person years in the included studies was greater than 940 000.	No downgrading
Publication Bias	According to the funnel plot, there were no sign of publication bias/funnel plot asymmetry.	No downgrading
Large Effect Size	Summary RR = 1.05. Insufficient information on unmeasured potential confounders available.	No upgrading
Confounding	Confounding direction unknown but precision may be affected.	No upgrading
Concentration-response	A linear dose–response relationship was assumed in all studies. 2 studies investigated the shape of the concentration-response relationship with no evidence to suggest non-linear. 95% CI for linear RR excluded 1.	Upgrade one level
**GRADE conclusion**	Downgrade one level and upgrade one level	HIGH CERTAINTY. Evidence for mean RR unadjusted for co-pollutants is 1.05 per 10 $\mu g/m^3$

Table S15. GRADE assessment – O_3 peak/warm period-All cause mortality (Global, 2023-2024).

**Domain**	**Rationale**	**Down/Up Grade**
Limitation in studies	N=12 included studies. Risk of bias moderate because although not all studies adjusted for all confounders, exclusion of high risk of bias studies did not alter significantly the summary RR .	No downgrading
Indirectness	All studies included the desired population, exposures and outcomes	No downgrading
Inconsistency	The 80% prediction interval included 1 & > twice CI (Fig. 2). High level of partly driven by regional differences	Downgrade one level
Imprecision	The number of person years in the included studies was greater than 940 000.	No downgrading
Publication Bias	According to the funnel plot and Egger's test (P < 0.1), there were no sign of publication bias/funnel plot asymmetry.	No downgrading
Large Effect Size	Summary RR = 1.01. Insufficient information on unmeasured potential confounders available.	No upgrading
Confounding	Confounding direction unknown but precision may be affected.	No upgrading
Concentration-response	A linear dose–response relationship was assumed in all studies. 7 studies investigated the shape of the concentration-response relationship with mixed evidence on potential linear, supra linear or threshold association; of these 7 only 3 indicated increased risk with increased levels. CI included 1.	No upgrading
**GRADE conclusion**	Downgrade one level and no upgrade	LOW CERTAINTY. Evidence for mean RR unadjusted for co-pollutants is 1.01 per 10 μg/m^3

Table S16. GRADE assessment – O_3 peak/warm period- Respiratory mortality (Global, 2023-2024).

**Domain**	**Rationale**	**Down/Up Grade**
Limitation in studies	N=9 included studies. Risk of bias moderate because although not all studies adjusted for all confounders, exclusion of high risk of bias studies did not reduce the summary RR.	No downgrading
Indirectness	All studies included the desired population, exposures and outcomes	No downgrading
Inconsistency	The 80% prediction interval included 1 & > twice CI. High level of heterogeneity not explained by region or pollutant level.	Downgrade one level
Imprecision	The number of person years in the included studies was greater than 940 000.	No downgrading
Publication Bias	According to the funnel plot, there were no sign of publication bias/funnel plot asymmetry.	No downgrading
Large Effect Size	Summary RR = 1.01. Insufficient information on unmeasured potential confounders available.	No upgrading
Confounding	Confounding direction unknown but precision may be affected.	No upgrading
Concentration-response	A linear dose–response relationship was assumed in all studies. 4 studies investigated the shape of the concentration-response relationship supporting linear or supra-linear shapes. 95% CI for linear RR included 1.	No upgrading
**GRADE conclusion**	Downgrade one level and upgrade one level	LOW CERTAINTY. Evidence for mean RR unadjusted for co-pollutants is 1.01 per 10 $\mu g/m^3$

Table S17. GRADE assessment – O_3 peak/warm period- COPD mortality (Global, 2023-2024).

**Domain**	**Rationale**	**Down/Up Grade**
Limitation in studies	N=7 included studies. Risk of bias low or moderate for all.	No downgrading
Indirectness	All studies included the desired population, exposures and outcomes	No downgrading
Inconsistency	The 80% prediction interval included 1 & > twice CI. High level of heterogeneity very partly explained by region and O3 levels.	Downgrade one level
Imprecision	The number of person years in the included studies was greater than 940 000.	No downgrading
Publication Bias	According to the funnel plot, there were no sign of publication bias/funnel plot asymmetry.	No downgrading
Large Effect Size	Summary RR = 1.00. Insufficient information on unmeasured potential confounders available.	No upgrading
Confounding	Confounding direction unknown but precision may be affected.	No upgrading
Concentration-response	A linear dose–response relationship was assumed in all studies. 3 studies investigated the shape of the concentration-response relationship with no evidence to suggest non-linear. 95% CI for linear RR included 1.	No upgrading
**GRADE conclusion**	Downgrade one level and upgrade one level	LOW CERTAINTY. Evidence for mean RR unadjusted for co-pollutants is 1.00 per 10 $\mu g/m^3$

Table S18. GRADE assessment – O_3 peak/warm period- ALRI mortality (Global, 2023-2024).

Domain	Rationale	Down/Up Grade
Limitation in studies	N=4 included studies. Risk of bias low or moderate for all.	No downgrading
Indirectness	All studies included the desired population, exposures and outcomes	No downgrading
Inconsistency	The 80% prediction interval included 1 & > twice CI. High level of partly explained by region and O3 levels.	Downgrade one level
Imprecision	The number of person years in the included studies was greater than 940 000.	No downgrading
Publication Bias	According to the funnel plot, there were no sign of publication bias/funnel plot asymmetry.	No downgrading
Large Effect Size	Summary RR = 1.02. Insufficient information on unmeasured potential confounders available.	No upgrading
Confounding	Confounding direction unknown but precision may be affected.	No upgrading
Concentration-response	A linear dose–response relationship was assumed in all studies. 2 studies investigated the shape of the concentration-response relationship with no evidence to suggest non-linear. 95% CI for linear RR included 1.	No upgrading
**GRADE conclusion**	Downgrade one level and upgrade one level	LOW CERTAINTY. Evidence for mean RR unadjusted for co-pollutants is 1.02 per 10 $\mu g/m^3$

APPENDIX S1. METHODS

A protocol was registered at the International Prospective Register of Systematic Reviews (PROSPERO, reference number: CRD42023425327).

Eligibility criteria

The inclusion and exclusion criteria were based on the PECOS as follows: For the Population (P) the General (human) population of all ages; for Exposure the long-term exposure (in the order of months to years) to ambient NO_2 and O_3, considering O_3 either for the annual or peak/warm season; for Comparator (C) exposure to lower pollutants levels in the same population (difference of 10 μg/m³) and; for Outcomes (O) for NO_2: all-cause mortality (10th revision of the International Classification of Diseases; 9th revision of the International Classification of Diseases (ICD-10): A00–Z99; ICD-9: 001-799), mortality due to circulatory diseases (ICD10: I00–I99; ICD-9: 390-459), ischemic heart diseases (IHD) (ICD10: I20–I25; ICD9: 410–414), cerebrovascular diseases (ICD10: I60–I69; ICD9: 430–438), respiratory diseases (ICD10: J00–J99; ICD9: 460-519), chronic obstructive pulmonary disease (COPD) (ICD10: J40–J44, ICD10: J47; ICD9: 490-496), acute lower respiratory infections (ALRI) (ICD10: J12–J18, ICD10: J20–J22; ICD9: 480-487) and lung cancer (ICD10: C33–C34); and for O_3: all-cause mortality (ICD10: A00–Z99; ICD-9: 001-799), mortality due to respiratory diseases (ICD10: J00–J99; ICD9: 460-519), chronic obstructive pulmonary disease (COPD) (ICD10: J40–J44, ICD10: J47; ICD9: 490-496), and acute lower respiratory infections (ALRI) (ICD10: J12–J18, ICD10: J20–J22; ICD9: 480-487). Finally, as Study design (S) we considered prospective and retrospective cohorts.

Studies were excluded if they did not correspond to the PECOS or if they a) were on patient populations, b) were on indoor, occupational or pregnancy exposures, c) were not published in English, d) did not use original data or were methodological studies, e) used a categorical exposure increment for the association, f) had insufficient information to standardize the measure of the association and its precision (standard error or confidence interval) or were g) toxicological studies (e.g. in vivo, in vitro), h) conference abstracts, qualitative studies or systematic reviews and meta-analyses.

Information sources

We searched the PubMed and EMBASE databases for studies published from September 2018 that was the last search date that informed the previous WHO AQG SR, up to May 2023. (Supplement Appendix S2 Table A1). As the current review includes mortality due to circulatory, cerebrovascular and ischemic heart diseases for NO_2 not considered in the previous SR [3], we reassessed articles excluded in the previous SR and published before September 2018. An updated search was performed in January 2024 to identify articles published after May 2023 to enrich the discussion of the current report.

Study selection

ES and PO independently assessed to ascertain compliance with the eligibility criteria and resolved disagreements through discussion. When multiple publications using the same cohorts were identified, we selected the study reporting on the largest cohort sample size, or as a second criterion the most recent paper.

A publication was not included in the meta-analysis if it reported on a specific cohort that was part of greater collaborative project and publication reporting pooled effects between multiple cohorts. We selected the publication that reported the pooled results (and extracted the pooled effect estimate) of the analyzed cohorts and not cohort specific results or cohort specific papers. This decision 1) reduces heterogeneity due to exposure assessment and statistical methods between cohorts and 2) avoids including the same (potentially large administrative) cohort many times in the meta-analysis. Only if the cohort baseline sample size differed by more than 25% or the number of additional years reported in a cohort-specific paper was equal to or greater than the number of overlapping years with those contributing to the pooled analysis, the decision was to include both papers (the pooled analysis and the cohort-specific).

Data collection
Data from newly identified studies were extracted independently by ES and MIK and added to previous review results in an Excel spreadsheet (Supplemental excel file 1). We retrieved information on: publication details (title, authors, date of publication); study characteristics (study design, location); study population; exposure details (concentration of the pollutant; co-exposures; lag patterns); outcome assessment; exposure unit of measurement; risk estimate as measure of association with 95% confidence interval (CI) with corresponding exposure increment.

We extracted data from the single-pollutant models for each pollutant-outcome of interest. When multiple estimates were reported, we selected those reported by study authors as the "main" model in the methods section in accordance with the previous reviews [2,3]. In cases when a "main" model was not clearly identified we extracted estimates from the model that included most of the critical confounders (age, sex, body mass index (BMI), socio-economic status (SES)), while for articles that could not be included in the previous classification, a case-by-case assessment was applied taking into account the sample contributing to each model or other relevant ad hoc criteria. We conducted four corresponding authors to provide information [33,71,87,88].

Data Synthesis - Meta-analysis
Effect estimates (Hazard Ratios (HR) or Relative Risks (RR) and 95% CIs) from single pollutant models were extracted from the selected studies. HRs were considered estimates of relative risks (RR). In cases where the effect estimates were presented per increase in parts per billion (ppb), we converted them to μg/m^3 using standard factors (1.88 for NO_2 and 1.96 for O_3) [4]. When the RRs were reported in increments other than per 10 μg/m^3 we calculated the natural logarithm of the RRs and the CIs, and then standardizing to 10 μg/m^3 by diving by the reported increment and multiplying by 10.

For each pollutant-outcome pair, we applied a random-effects meta-analysis using the restricted maximum likelihood (REML) method for the estimation of between studies variability [3]. Heterogeneity between studies was assessed by calculating 80% prediction intervals (80% PI), indicating heterogeneity if the PI includes the null effect and its width is twice that of the 95% CI. We also used the Cochran's Q test and the I^2 statistic to assess heterogeneity. Publication bias was assessed by funnel plots, and also the Eggers' test if there were 10 studies or more contributing to the meta-analysis. In the interpretation of the funnel pot asymmetry and the publication bias, we took into account heterogeneity and asymmetry around the null.

We performed subgroup analysis to investigate heterogeneity by a) WHO regions (European Region; Region of the Americas; Western Pacific Region and b) Risk of bias assessment per domain in cases the meta-analyses included studies at high risk of bias for the relevant domain (High vs Low/Moderate). We further conducted a meta-regression to assess the impact of the mean level of pollutant concentrations on the effect estimates, in case of 10 studies or more contributed to the meta-analysis. If less than 10 studies were included, we performed a subgroup analysis by the median of the level of the mean pollutant (above median vs below).

Risk of bias Assessment
Individual studies, included in the meta-analysis, were evaluated for the risk of bias (RoB), using the tool developed by a working group convened by WHO for the AQG systematic reviews [3, 5]. In short, the RoB tool includes six domains on confounding, selection bias, exposure assessment, outcome measurement, missing data, and selective reporting. Each domain includes up to four sub-domains, each rated as low, moderate or high risk of bias. Only if all the subdomains are assessed as low risk of bias, the entire domain is rated as low. When at least one subdomain gets a moderate rating and none of the subdomains get a high score, the overall domain is rated as of moderate risk. RoB total score per domain is rated as high risk if any of the subdomains received a high rating. No overall risk of bias is assigned across domains.

Evaluation of certainty of evidence
For each pollutant – outcome pair, we assessed the certainty of evidence using the modified GRADE (Grading of Recommendations, Assessment, Development and Evaluation) [6], adapted by a working group of experts under the

supervision of the WHO Secretariat in the context of the update of the AQGs. In brief, the GRADE instrument includes eight domains, with the initial level of certainty being “moderate” in each domain. After evaluating the evidence, the certainty of evidence could be: **Downgraded** for: 1) Limitations in studies, indicated by differences in the pooled estimates when considering all studies or only those rated “low” in the RoB. The limited influence of high-risk bias studies was not considered a reason for downgrading; 2) Indirectness, if the studies did not address the PECOS 3) If substantial heterogeneity was suspected, as indicted by the PI, that cannot be explained 4) Imprecision, if the number of person-years of follow-up was less than 940 000 person-years and 5) Publication bias. Certainty in the evidence is **Upgraded** when: 1) the impact of potential missing confounders on the Effect size is expected to be minimal 2) the pooled RR remains significant after adjustment for all plausible confounding, after shifting the RR towards the null and 3) indication of linearity or increased risk with increased pollutant levels among studies that investigated the concentration-response function and if the 95%CI of the RR was above 1.

The overall certainty assessment of evidence combines the downgrades and upgrades across domains resulting in an overall rating of high, if additional research is very unlikely to alter the certainty; moderate, if additional research is likely to have a substantial impact on the certainty; low, if additional research is very likely to have a substantial impact on the certainty and very low if the effect is of high uncertainty.

APPENDIX S2.

Table A1. Search Strategy (Global, 2023-2024).

A) PUBMED (01/09/2018 to 22/05/2024)

(mortality[MH] OR death*[MH] or mortality[TIAB] OR death*[TIAB]) AND (cohort*[TIAB] OR cox[TIAB] OR hazard*[TIAB]) AND (air pollution[MH] OR PM10[TIAB] OR PM2[TIAB] OR particle*[TIAB] OR particulate*[TIAB] OR ozone[TIAB] OR O3[TIAB] OR NO2[TIAB] OR nitrogen dioxide[TIAB])

B) EMBASE (2018 to 22/05/2024)

((mortality or death).sh or (mortality or death).tw) and (air pollution.sh or (particle* or particulate* or PM10 or PM2* or nitrogen dioxide or NO2 or ozone or O3).tw) and (cohort* or cox or hazard).tw

Forty-four reviews and 89 original studies were excluded. Details for the reasons of exclusion are presented below in Table A2. In brief, 24 studies did not provide a quantitative HR, 20 studies were excluded due to other outcomes, eight studies were excluded due to assessing other pollutants and 14 studies were excluded for other reasons. Finally, we excluded 9 duplicate studies and 14 conference abstracts. Seventy-four studies were excluded as they assessed only exposure to particles.

Table A2. List of excluded studies with rationale **(N=89)** (Global, 2023-2024).

No quantitative hazard ratio provided (N=24)

Pollutants used only for adjustment

1. Bauwelinck, M. Casas, L. Nawrot, T. S. Nemery, B. Trabelsi, S. Thomas, I. et al., Residing in urban areas with higher green space is associated with lower mortality risk: A census-based cohort study with ten years of follow-up. Environ Int. 2021; 10.1016/j.envint.2020.106365
2. Bereziartua, A. Chen, J. de Hoogh, K. Rodopoulou, S. Andersen, Z. J. Bellander, T. et al., Exposure to surrounding greenness and natural-cause and cause-specific mortality in the ELAPSE pooled cohort. Environ Int. 2022; 10.1016/j.envint.2022.107341
3. Bustaffa, E. Curzio, O. Donzelli, G. Gorini, F. Linzalone, N. Redini, M. et al., Risk Associations between Vehicular Traffic Noise Exposure and Cardiovascular Diseases: A Residential Retrospective Cohort Study. Int J Environ Res Public Health. 2022; 10.3390/ijerph191610034
4. Cakmak, S. Hebbern, C. Vanos, J. Crouse, D. L. Tjepkema, M. Exposure to traffic and mortality risk in the 1991-2011 Canadian Census Health and Environment Cohort (CanCHEC). Environ Int. 2019; 10.1016/j.envint.2018.12.045
5. Cole-Hunter, T. So, R. Amini, H. Backalarz, C. Brandt, J. Bräuner, E. V. et al., Long-term exposure to road traffic noise and all-cause and cause-specific mortality: a Danish Nurse Cohort study. Sci Total Environ. 2022; 10.1016/j.scitotenv.2022.153057
6. Lim, Y. H. Jørgensen, J. T. So, R. Cramer, J. Amini, H. Mehta, A. et al., Long-term exposure to road traffic noise and incident myocardial infarction: A Danish Nurse Cohort study. Environ Epidemiol. 2021; 10.1097/ee9.0000000000000148
7. Lin, Y. Yang, X. Liang, F. Huang, K. Liu, F. Li, J. et al., Benefits of active commuting on cardiovascular health modified by ambient fine particulate matter in China: A prospective cohort study. Ecotoxicol Environ Saf. 2021; 10.1016/j.ecoenv.2021.112641
8. Miguet, M. Venetis, S. Rukh, G. Lind, L. Schiöth, H. B. Time spent outdoors and risk of myocardial infarction and stroke in middle and old aged adults: Results from the UK Biobank prospective cohort. Environ Res. 2021; 10.1016/j.envres.2021.111350
9. Rodopoulou, S. Stafoggia, M. Chen, J. de Hoogh, K. Bauwelinck, M. Mehta, A. J. et al., Long-term exposure to fine particle elemental components and mortality in Europe: Results from six European administrative cohorts within the ELAPSE project. Sci Total Environ. 2022; 10.1016/j.scitotenv.2021.152205
10. Roswall, N. Pyko, A. Ögren, M. Oudin, A. Rosengren, A. Lager, A. et al., Long-Term Exposure to Transportation Noise and Risk of Incident Stroke: A Pooled Study of Nine Scandinavian Cohorts. Environ Health Perspect. 2021; 10.1289/ehp8949
11. Sørensen, M. Raaschou-Nielsen, O. Poulsen, A. H. Hvidtfeldt, U. A. Brandt, J. Khan, J. et al., Long-term exposure to residential transportation noise and mortality: A nationwide cohort study. Environ Pollut. 2023; 10.1016/j.envpol.2023.121642
12. Thacher, J. D. Hvidtfeldt, U. A. Poulsen, A. H. Raaschou-Nielsen, O. Ketzel, M. Brandt, J. et al., Long-term residential road traffic noise and mortality in a Danish cohort. Environ Res. 2020; 10.1016/j.envres.2020.109633
13. Vienneau, D. Saucy, A. Schäffer, B. Flückiger, B. Tangermann, L. Stafoggia, M. et al., Transportation noise exposure and cardiovascular mortality: 15-years of follow-up in a nationwide prospective cohort in Switzerland. Environ Int. 2022; 10.1016/j.envint.2021.106974
14. Wan, S. Rojas-Rueda, D. Pretty, J. Roscoe, C. James, P. Ji, J. S. Greenspace and mortality in the U.K. Biobank: Longitudinal cohort analysis of socio-economic, environmental, and biomarker pathways. SSM - Population Health. 2022; 10.1016/j.ssmph.2022.101194
15. Heritier, H. Vienneau, D. Foraster, M. Eze, I. C. Schaffner, E. Thiesse, L. et al., Diurnal variability of transportation noise exposure and cardiovascular mortality: A nationwide cohort study from Switzerland. International journal of hygiene and environmental health. 2018; 10.1016/j.ijheh.2018.02.005

16. Chen, H. Burnett, R. T. Bai, L. Kwong, J. C. Crouse, D. L. Lavigne, E. et al., Residential greenness and cardiovascular disease incidence, readmission, and mortality. Environmental Health Perspectives. 2020; 10.1289/EHP6161

Pollutants used only as mediators

1. Orioli, R. Antonucci, C. Scortichini, M. Cerza, F. Marando, F. Ancona, C. et al., Exposure to Residential Greenness as a Predictor of Cause-Specific Mortality and Stroke Incidence in the Rome Longitudinal Study. Environ Health Perspect. 2019; 10.1289/ehp2854
2. Pan, W. C. Yeh, S. Y. Wu, C. D. Huang, Y. T. Chen, Y. C. Chen, C. J. et al., Association Between Traffic Count and Cardiovascular Mortality: A Prospective Cohort Study in Taiwan. J Epidemiol. 2021; 10.2188/jea.JE20200082
3. Rodriguez-Loureiro, L. Verdoodt, F. Lefebvre, W. Vanpoucke, C. Casas, L. Gadeyne, S. Long-term exposure to residential green spaces and site-specific cancer mortality in urban Belgium: A 13-year follow-up cohort study. Environment International. 2022; 10.1016/j.envint.2022.107571
4. Rodriguez-Loureiro, L. Casas, L. Bauwelinck, M. Lefebvre, W. Vanpoucke, C. Vanroelen, C. et al., Social inequalities in the associations between urban green spaces, self-perceived health and mortality in Brussels: Results from a census-based cohort study. Health and Place. 2021; 10.1016/j.healthplace.2021.102603

No HR - Rate difference

1. Bai, L. Benmarhnia, T. Chen, C. Kwong, J. C. Burnett, R. T. van Donkelaar, A. et al., Chronic Exposure to Fine Particulate Matter Increases Mortality Through Pathways of Metabolic and Cardiovascular Disease: Insights from a Large Mediation Analysis. J Am Heart Assoc. 2022; 10.1161/jaha.122.026660

No overall HR

1. Thomson, E. M. Christidis, T. Pinault, L. Tjepkema, M. Colman, I. Crouse, D. L. et al., Self-rated stress, distress, mental health, and health as modifiers of the association between long-term exposure to ambient pollutants and mortality. Environ Res. 2020; 10.1016/j.envres.2020.109973
2. Wei, Y. Coull, B. Koutrakis, P. Yang, J. Li, L. Zanobetti, A. et al., Assessing additive effects of air pollutants on mortality rate in Massachusetts. Environ Health. 2021; 10.1186/s12940-021-00704-3
3. Lipfert, F. W. Wyzga, R. E. Environmental predictors of survival in a cohort of U.S. military veterans: A multi-level spatio-temporal analysis stratified by race. Environ Res. 2020; 10.1016/j.envres.2019.108842

Not outcome of interest (N = 20)

Incidence of Disease

1. Bai, L. Shin, S. Burnett, R. T. Kwong, J. C. Hystad, P. van Donkelaar, A. et al., Exposure to ambient air pollution and the incidence of congestive heart failure and acute myocardial infarction: A population-based study of 5.1 million Canadian adults living in Ontario. Environ Int. 2019; 10.1016/j.envint.2019.105004
2. Bai, L. Weichenthal, S. Kwong, J. C. Burnett, R. T. Hatzopoulou, M. Jerrett, M. et al., Associations of Long-Term Exposure to Ultrafine Particles and Nitrogen Dioxide With Increased Incidence of Congestive Heart Failure and Acute Myocardial Infarction. Am J Epidemiol. 2019; 10.1093/aje/kwy194
3. Gowda, S. N. DeRoos, A. J. Hunt, R. P. Gassett, A. J. Mirabelli, M. C. Bird, C. E. et al., Ambient air pollution and lung cancer risk among never-smokers in the Women's Health Initiative. Environ Epidemiol. 2019; 10.1097/ee9.0000000000000076
4. Huang, Y. J. Lee, P. H. Chen, L. C. Lin, B. C. Lin, C. Chan, T. C. Relationships among green space, ambient fine particulate matter, and cancer incidence in Taiwan: A 16-year retrospective cohort study. Environ Res. 2022; 10.1016/j.envres.2022.113416
5. Li, J. Lu, X. Liu, F. Liang, F. Huang, K. Yang, X. et al., Chronic Effects of High Fine Particulate Matter Exposure on Lung Cancer in China. Am J Respir Crit Care Med. 2020; 10.1164/rccm.202001-0002OC
6. Ma, T. Yazdi, M. D. Schwartz, J. Réquia, W. J. Di, Q. Wei, Y. et al., Long-term air pollution exposure and incident stroke in American older adults: A national cohort study. Glob Epidemiol. 2022; 10.1016/j.gloepi.2022.100073
7. Poulsen, A. H. Sørensen, M. Hvidtfeldt, U. A. Brandt, J. Frohn, L. M. Ketzel, M. et al., Source-specific' air pollution and risk of stroke in Denmark. Int J Epidemiol. 2023; 10.1093/ije/dyad030

8. Wolf, K. Hoffmann, B. Andersen, Z. J. Atkinson, R. W. Bauwelinck, M. Bellander, T. et al., Long-term exposure to low-level ambient air pollution and incidence of stroke and coronary heart disease: a pooled analysis of six European cohorts within the ELAPSE project. Lancet Planet Health. 2021; 10.1016/s2542-5196(21)00195-9
9. Wright, N. Newell, K. Chan, K. H. Gilbert, S. Hacker, A. Lu, Y. et al., Long-term ambient air pollution exposure and cardio-respiratory disease in China: findings from a prospective cohort study. Environ Health. 2023; 10.1186/s12940-023-00978-9
10. Yu, K. Zhang, Q. Meng, X. Zhang, L. Kan, H. Chen, R. Association of residential greenness with incident chronic obstructive pulmonary disease: A prospective cohort study in the UK Biobank. Environ Int. 2023; 10.1016/j.envint.2022.107654
11. Wang, L. Xie, J. Hu, Y. Tian, Y. Air pollution and risk of chronic obstructed pulmonary disease: The modifying effect of genetic susceptibility and lifestyle. eBioMedicine. 2022; 10.1016/j.ebiom.2022.103994
12. Grande, G. Ljungman, P. L. S. Eneroth, K. Bellander, T. Rizzuto, D. Association Between Cardiovascular Disease and Long-term Exposure to Air Pollution With the Risk of Dementia. JAMA Neurol. 2020; 10.1001/jamaneurol.2019.4914

Other Causes of Death

1. Bo, Y. Yu, T. Chang, L. Y. Guo, C. Lin, C. Zeng, Y. et al., Combined effects of chronic PM2.5 exposure and habitual exercise on cancer mortality: a longitudinal cohort study. Int J Epidemiol. 2022; 10.1093/ije/dyab209
2. Canterbury, A. Echouffo-Tcheugui, J. B. Shpilsky, D. Aiyer, A. Reis, S. E. Erqou, S. Association between cumulative social risk, particulate matter environmental pollutant exposure, and cardiovascular disease risk. BMC Cardiovasc Disord. 2020; 10.1186/s12872-020-01329-z
3. Dalecká, A. Wigmann, C. Kress, S. Altug, H. Jiřík, V. Heinrich, J. et al., The mediating role of lung function on air pollution-induced cardiopulmonary mortality in elderly women: The SALIA cohort study with 22-year mortality follow-up. Int J Hyg Environ Health. 2021; 10.1016/j.ijheh.2021.113705
4. Hart, J. E. Hohensee, C. Laden, F. Holland, I. Whitsel, E. A. Wellenius, G. A. et al., Long-Term Exposures to Air Pollution and the Risk of Atrial Fibrillation in the Women's Health Initiative Cohort. Environ Health Perspect. 2021; 10.1289/ehp7683
5. Ljungman, P. L. S. Andersson, N. Stockfelt, L. Andersson, E. M. Nilsson Sommar, J. Eneroth, K. et al., Long-Term Exposure to Particulate Air Pollution, Black Carbon, and Their Source Components in Relation to Ischemic Heart Disease and Stroke. Environ Health Perspect. 2019; 10.1289/ehp4757
6. Pyko, A. Roswall, N. Ogren, M. Oudin, A. Rosengren, A. Eriksson, C. et al., Long-term exposure to transportation noise and ischemic heart disease: A pooled analysis of nine scandinavian cohorts. Environmental Health Perspectives. 2023; 10.1289/EHP10745

Not outcome of interest (composite outcome of fatal and non-fatal stroke)

1. Xu, Y. Chen, J. T. Holland, I. Yanosky, J. D. Liao, D. Coull, B. A. et al., Analysis of long- and medium-term particulate matter exposures and stroke in the US-based Health Professionals Follow-up Study. Environ Epidemiol. 2021; 10.1097/ee9.0000000000000178

Not outcome of interest (CVD incidence and all-cause mortality combined)

1. Erqou, S. Clougherty, J. E. Olafiranye, O. Magnani, J. W. Aiyer, A. Tripathy, S. et al., Particulate Matter Air Pollution and Racial Differences in Cardiovascular Disease Risk. Arteriosclerosis, Thrombosis, and Vascular Biology. 2018; 10.1161/ATVBAHA.117.310305

Other Pollutants (N=8)

Second-hand smoke

1. Akiba, S. Kinjo, Y. Japanese Legacy Cohorts: Six-Prefecture Cohort Study (Hirayama Cohort Study). J Epidemiol. 2020; 10.2188/jea.JE20190249

NOx

1. Andersson, E. M. Ögren, M. Molnár, P. Segersson, D. Rosengren, A. Stockfelt, L. Road traffic noise, air pollution and cardiovascular events in a Swedish cohort. Environ Res. 2020; 10.1016/j.envres.2020.109446

Total particulate pollution

1. Genowska, A. Strukcinskiene, B. Jamiołkowski, J. Abramowicz, P. Konstantynowicz, J. Emission of Industrial Air Pollution and Mortality Due to Respiratory Diseases: A Birth Cohort Study in Poland. Int J Environ Res Public Health. 2023; 10.3390/ijerph20021309

Mixtures

1. Li, H. Deng, W. Small, R. Schwartz, J. Liu, J. Shi, L. Health effects of air pollutant mixtures on overall mortality among the elderly population using Bayesian kernel machine regression (BKMR). Chemosphere. 2022; 10.1016/j.chemosphere.2021.131566

PM components

1. So, R. Chen, J. Stafoggia, M. de Hoogh, K. Katsouyanni, K. Vienneau, D. et al., Long-term exposure to elemental components of fine particulate matter and all-natural and cause-specific mortality in a Danish nationwide administrative cohort study. Environ Res. 2023; 10.1016/j.envres.2023.115552
2. Chen, J.; Rodopoulou, S.; de Hoogh, K.; Strak, M.; Andersen, Z. J.; Atkinson, R. et al., Long-Term Exposure to Fine Particle Elemental Components and Natural and Cause-Specific Mortality-a Pooled Analysis of Eight European Cohorts within the ELAPSE Project. Environ Health Perspect. 2021; 10.1289/ehp8368

Industrial PM_{10}

1. Leograde, S. Alessandrini, E. R. Stafoggia, M. Morabito, A. Nocioni, A. Ancona, C.et al., Industrial air pollution and mortality in the Taranto area, Southern Italy: A difference-in-differences approach .Environ Int .2019;10.1016/j.envint.2019.105030

Other Reasons (N = 14)

Categorical increment

1. Khadka, A.; Canning, D. Understanding the Pathways from Prenatal and Post-Birth PM(2.5) Exposure to Infant Death: An Observational Analysis Using US Vital Records (2011-2013). Int J Environ Res Public Health. 2021; 10.3390/ijerph19010258
2. Kotecha, S. J.; Watkins, W. J.; Lowe, J.; Grigg, J.; Kotecha, S. Differential association of air pollution exposure with neonatal and postneonatal mortality in England and Wales: A cohort study. PLoS Med. 2020; 10.1371/journal.pmed.1003400
3. Ku, P. W.; Steptoe, A.; Lai, Y. J.; Yen, Y. F.; Ahmadi, M.; Inan-Eroglu, E. et al., Are associations of leisure-time physical activity with mortality attenuated by high levels of chronic ambient fine particulate matter (PM(2.5)) in older adults? A prospective cohort study. Exp Gerontol.. 2023; 10.1016/j.exger.2023.112148
4. Hadley, M. B. Nalini, M. Adhikari, S. Szymonifka, J. Etemadi, A. Kamangar, F.et al., Spatial environmental factors predict cardiovascular and all-cause mortality: Results of the SPACE study .PLoS One .2022;10.1371/journal.pone.0269650

Patient population

1. Lipfert, F. W.; Wyzga, R. E. Revisiting the Veterans Cohort Mortality Study: New results and synthesis. J Air Waste Manag Assoc. 2018; 10.1080/10962247.2018.1498409

2. Huang, H. L.; Chuang, Y. H.; Lin, T. H.; Lin, C.; Chen, Y. H.; Hung, J. Y. et al., Ambient Cumulative PM2.5 Exposure and the Risk of Lung Cancer Incidence and Mortality: A Retrospective Cohort Study. Int J Environ Res Public Health. 2021; 10.3390/ijerph182312400

Binary exposures

1. Wolf, K.; Rodopoulou, S.; Chen, J.; Andersen, Z. J.; Atkinson, R. W.; Bauwelinck, M. et al., Comparison of traditional Cox regression and causal modeling to investigate the association between long-term air pollution exposure and natural-cause mortality within European cohorts. Environ Pollut. 2023; 10.1016/j.envpol.2023.121515

Modelling study using previous CRFs

1. Castro, A.; Künzli, N.; de Hoogh, K.; Kappeler, R.; Kutlar Joss, M.; Vienneau, D. et al., Mortality attributable to ambient fine particulate matter and nitrogen dioxide in Switzerland in 2019: Use of two-pollutant effect estimates. Environ Res. 2023; 10.1016/j.envres.2023.116029

No cohort design

1. Jung, E. M.; Kim, K. N.; Park, H.; Shin, H. H.; Kim, H. S.; Cho, S. J. et al., Association between prenatal exposure to PM(2.5) and the increased risk of specified infant mortality in South Korea. Environ Int. 2020; 10.1016/j.envint.2020.105997
2. Renzi, M.; Forastiere, F.; Schwartz, J.; Davoli, M.; Michelozzi, P.; Stafoggia, M. Long-Term PM(10) Exposure and Cause-Specific Mortality in the Latium Region (Italy): A Difference-in-Differences Approach. Environ Health Perspect. 2019; 10.1289/ehp3759
3. Ghosh, R. E.; Freni-Sterrantino, A.; Douglas, P.; Parkes, B.; Fecht, D.; de Hoogh, K. et al., Fetal growth, stillbirth, infant mortality and other birth outcomes near UK municipal waste incinerators; retrospective population based cohort and case-control study. Environ Int. 2019; 10.1016/j.envint.2018.10.060

Measurement error correction

1. Feng, Y.; Wei, Y.; Coull, B. A.; Schwartz, J. D. Measurement error correction for ambient PM(2.5) exposure using stratified regression calibration: Effects on all-cause mortality. Environ Res. 2023; 10.1016/j.envres.2022.114792

Ozone and cardiovascular mortality

1. Liu, S.; Zhang, Y.; Ma, R.; Liu, X.; Liang, J.; Lin, H. et al., Long-term exposure to ozone and cardiovascular mortality in a large Chinese cohort. Environ Int. 2022; 10.1016/j.envint.2022.107280
2. Niu, Y.; Zhou, Y.; Chen, R.; Yin, P.; Meng, X.; Wang, W. et al., Long-term exposure to ozone and cardiovascular mortality in China: a nationwide cohort study. The Lancet Planetary Health. 2022; 10.1016/S2542-5196%2822%2900093-6

Duplicate Studies (N = 9)

1. Hystad, P. Larkin, A. Rangarajan, S. AlHabib, K. F. Avezum, Á Calik, K. B. T. et al., Associations of outdoor fine particulate air pollution and cardiovascular disease in 157 436 individuals from 21 high-income, middle-income, and low-income countries (PURE): a prospective cohort study. Lancet Planet Health. 2020; 10.1016/s2542-5196(20)30103-0
2. Niu, Y. Zhou, Y. Chen, R. Yin, P. Meng, X. Wang, W. et al., Long-term exposure to ozone and cardiovascular mortality in China: a nationwide cohort study. Lancet Planet Health. 2022; 10.1016/s2542-5196(22)00093-6
3. Yazdi, M. D. Wang, Y. Di, Q. Requia, W. J. Wei, Y. Shi, L. et al., Long-term effect of exposure to lower concentrations of air pollution on mortality among US Medicare participants and vulnerable subgroups: a doubly-robust approach. The Lancet Planetary Health. 2021; 10.1016/S2542-5196%2821%2900204-7
4. Yang, Y. Tang, R. Qiu, H. Lai, P. C. Wong, P. Thach, T. Q. et al., Long term exposure to air pollution and mortality in an elderly cohort in Hong Kong. Environment International. 2018; 10.1016/j.envint.2018.04.034
5. Wolf, K. Hoffmann, B. Andersen, Z. J. Atkinson, R. W. Bauwelinck, M. Bellander, T. et al., Long-term exposure to low-level ambient air pollution and incidence of stroke and coronary heart disease: a pooled

analysis of six European cohorts within the ELAPSE project. The Lancet Planetary Health. 2021; 10.1016/S2542-5196%2821%2900195-9

6. Stafoggia, M. Oftedal, B. Chen, J. Rodopoulou, S. Renzi, M. Atkinson, R. W. et al., Long-term exposure to low ambient air pollution concentrations and mortality among 28 million people: results from seven large European cohorts within the ELAPSE project. The Lancet Planetary Health. 2022; 10.1016/S2542-5196%2821%2900277-1
7. Cakmak, S. Hebbern, C. Pinault, L. Lavigne, E. Vanos, J. Crouse, D. L. et al., Associations between long-term PM2.5 and ozone exposure and mortality in the Canadian Census Health and Environment Cohort (CANCHEC), by spatial synoptic classification zone. Environment International. 2018; 10.1016/j.envint.2017.11.030
8. Bert, B. Maciej, S. Jie, C. Zorana, J. A. Richard, A. Mariska, B. et al., Mortality and Morbidity Effects of Long-Term Exposure to Low-Level PM2.5, BC, NO2, and O3: An Analysis of European Cohorts in the ELAPSE Project. Research report (Health Effects Institute). 2021
9. Li, T. Zhang, Y. Wang, J. Xu, D. Yin, Z. Chen, H.et al., All-cause mortality risk associated with long-term exposure to ambient PM2.5 in China: a cohort study .The Lancet Public Health .2018;10.1016/S2468-2667%2818%2930144-0

Conference Abstracts (N = 14)

1. Xu, S. Marcon, A. Bertelsen, R. J. Benediktsdottir, B. Geels, C. Gislason, T.et al., Associations of low-level air pollution and greenness with mortality in men and women. The Life- GAP project. European Clinical Respiratory Journal. 2022; 10.1080/20018525.2022.2058255
2. Schikowski, T. Dalecka, A. Wigmann, C. Altug, H. Jirik, V. Heinrich, J.et al., Effect of air pollution and lung function on cardiopulmonary mortality in aging women. American Journal of Respiratory and Critical Care Medicine. Conference: American Thoracic Society International Conference, ATS. 2020;
3. Ottone, M. Broccoli, S. Giannini, S. Scotto, F. Parmagnani, F. Bonvicini, L.et al., Source-related components of PM2.5 and long-term health effects: Epidemiological findings of supersite project in Italy. Occupational and Environmental Medicine. 2018; 10.1136/oemed-2018-ISEEabstracts.73
4. Oftedal, B. Bekkevold, T. Nordheim, C. F. Kristoffersen, D. T. Krog, N. H. Aasvang, G. M.et al., Air pollution and mortality in Norway-using NORCOHORT. Norsk Epidemiologi. 2019;
5. Lo, L. W. Tsai, T. Y. Huang, Y. S. Leu, H. B. Chen, S. A. Ten-Year Effects of Ambient Pm2.5 Exposure Significantly Increase Sudden Cardiac Death, Cardiovascular and All-Cause Mortalities: A Nationwide Cohort Study. Heart Rhythm. 2023; 10.1016/j.hrthm.2023.03.926
6. Lim, C. C. Hayes, R. Ahn, J. Shao, Y. Thurston, G. D. Air pollution, mediterranean diet, and cause-specific mortality risk in the NIH-AARP diet and health study. American Journal of Respiratory and Critical Care Medicine. Conference: American Thoracic Society International Conference, ATS. 2018;
7. Kim, I. S. Yang, P. S. Kim, T. H. Uhm, J. S. Pak, H. N. Lee, M. H.et al., Particulate matter air pollution and mortality in a general healthy population: A nationwide cohort study. European Heart Journal. 2018; 10.1093/eurheartj/ehy565.P2515
8. Jung, Y. Effects of exposure to air pollution on ischemic stroke incidence and mortality. Journal of the Neurological Sciences. 2019; 10.1016/j.jns.2019.10.614
9. Ji, J. S. Zhu, A. Lv, Y. Shi, X. Residential greenness and air pollution mortality using the Chinese Longitudinal Healthy Longevity Survey: a longitudinal analysis. The Lancet. 2019; 10.1016/S0140-6736%2819%2932352-9
10. Ji, J. S. Zhu, A. Bai, C. James, P. Yan, L. Zeng, Y.et al., Residential greenness and mortality in oldest-old women and men in China: a prospective cohort study. The Lancet. 2018; 10.1016/S0140-6736%2818%2932694-1
11. Hadley, M. Nalini, M. Adhikari, S. Szymonifka, J. Etemadi, A. Kamangar, F.et al., Spatial risk factors impact cardiovascular mortality: Analysis from the Golestan cohort study. Circulation. Conference: American Heart Association Scientific Sessions, AHA. 2019; 10.1161/circ.140.suppl_1.13476

12. Dalecka, A. Wigmann, C. Altug, H. Kress, S. Jirik, V. Heinrich, J.et al., The mediating role of lung function on air pollution-induced cardiopulmonary mortality. European Respiratory Journal. Conference: European Respiratory Society International Congress, ERS. 2020; 10.1183/13993003.congress-2020.3846
13. Borchert, W. Hart, J. E. Nethery, R. Albert, C. M. Laden, F. Exposure to Multiple Size Fractions of Particulate Matter Air Pollution and Sudden Cardiac Death. Circulation. Conference: American Heart Association's. 2022; 10.1161/circ.146.suppl_1.14572
14. Bauwelinck, M. Casas, L. Deboosere, P. Trabelsi, S. Bijnens, E. Van Nieuwenhuyse, A.et al., Long-term exposure to neighbourhood green spaces protects from all-cause mortality in adults (18 to 65 years old) living in the five largest cities in Belgium: A census-based study. Occupational and Environmental Medicine. 2018; 10.1136/oemed-2018-ISEEabstracts.39

Excluded studies – Reviews (N=44)

1. Abed Al Ahad, M. Sullivan, F. Demšar, U. Melhem, M. Kulu, H. The effect of air-pollution and weather exposure on mortality and hospital admission and implications for further research: A systematic scoping review. PLoS One. 2020; 10.1371/journal.pone.0241415
2. Badida, P. Krishnamurthy, A. Jayaprakash, J. Meta analysis of health effects of ambient air pollution exposure in low- and middle-income countries. Environ Res. 2023; 10.1016/j.envres.2022.114604
3. Bekkar, B. Pacheco, S. Basu, R. DeNicola, N. Association of Air Pollution and Heat Exposure With Preterm Birth, Low Birth Weight, and Stillbirth in the US: A Systematic Review. JAMA Netw Open. 2020; 10.1001/jamanetworkopen.2020.8243
4. Boogaard, H. Samoli, E. Patton, A. P. Atkinson, R. W. Brook, J. R. Chang, H. H.et al., Long-term exposure to traffic-related air pollution and non-accidental mortality: A systematic review and meta-analysis. Environ Int. 2023; 10.1016/j.envint.2023.107916
5. Chen, J. Hoek, G. Long-term exposure to PM and all-cause and cause-specific mortality: A systematic review and meta-analysis. Environ Int. 2020; 10.1016/j.envint.2020.105974
6. Ciabattini, M. Rizzello, E. Lucaroni, F. Palombi, L. Boffetta, P. Systematic review and meta-analysis of recent high-quality studies on exposure to particulate matter and risk of lung cancer. Environ Res. 2021; 10.1016/j.envres.2020.110440
7. Colonna, K. J. Koutrakis, P. Kinney, P. L. Cooke, R. M. Evans, J. S. Mortality Attributable to Long-Term Exposure to Ambient Fine Particulate Matter: Insights from the Epidemiologic Evidence for Understudied Locations. Environ Sci Technol. 2022; 10.1021/acs.est.1c08343
8. Dimala, C. A. Kadia, B. M. A systematic review and meta-analysis on the association between ambient air pollution and pulmonary tuberculosis. Sci Rep. 2022; 10.1038/s41598-022-15443-9
9. Edwards, L. Wilkinson, P. Rutter, G. Milojevic, A. Health effects in people relocating between environments of differing ambient air pollution concentrations: A literature review. Environ Pollut. 2022; 10.1016/j.envpol.2021.118314
10. Gan, T. Bambrick, H. Tong, S. Hu, W. Air pollution and liver cancer: A systematic review. J Environ Sci (China). 2023; 10.1016/j.jes.2022.05.037
11. Guo, J. Chai, G. Song, X. Hui, X. Li, Z. Feng, X.et al., Long-term exposure to particulate matter on cardiovascular and respiratory diseases in low- and middle-income countries: A systematic review and meta-analysis. Front Public Health. 2023; 10.3389/fpubh.2023.1134341
12. Huang, S. Li, H. Wang, M. Qian, Y. Steenland, K. Caudle, W. M.et al., Long-term exposure to nitrogen dioxide and mortality: A systematic review and meta-analysis. Sci Total Environ. 2021; 10.1016/j.scitotenv.2021.145968
13. Huangfu, P. Atkinson, R. Long-term exposure to NO(2) and O(3) and all-cause and respiratory mortality: A systematic review and meta-analysis. Environ Int. 2020; 10.1016/j.envint.2020.105998
14. Jaganathan, S. Jaacks, L. M. Magsumbol, M. Walia, G. K. Sieber, N. L. Shivasankar, R.et al., Association of Long-Term Exposure to Fine Particulate Matter and Cardio-Metabolic Diseases in Low- and Middle-Income Countries: A Systematic Review. Int J Environ Res Public Health. 2019; 10.3390/ijerph16142541
15. Ju, L. Hua, L. Xu, H. Li, C. Sun, S. Zhang, Q.et al., Maternal atmospheric particulate matter exposure and risk of adverse pregnancy outcomes: A meta-analysis of cohort studies. Environ Pollut. 2023; 10.1016/j.envpol.2022.120704
16. Kihal-Talantikite, W. Marchetta, G. P. Deguen, S. Infant Mortality Related to NO(2) and PM Exposure: Systematic Review and Meta-Analysis. Int J Environ Res Public Health. 2020; 10.3390/ijerph17082623
17. Kim, H. B. Shim, J. Y. Park, B. Lee, Y. J. Long-Term Exposure to Air Pollutants and Cancer Mortality: A Meta-Analysis of Cohort Studies. Int J Environ Res Public Health. 2018; 10.3390/ijerph15112608
18. Krittanawong, C. Qadeer, Y. K. Hayes, R. B. Wang, Z. Virani, S. Thurston, G. D.et al., PM2.5 and Cardiovascular Health Risks. Curr Probl Cardiol. 2023; 10.1016/j.cpcardiol.2023.101670
19. Lipfert, F. W. Wyzga, R. E. Longitudinal relationships between lung cancer mortality rates, smoking, and ambient air quality: a comprehensive review and analysis. Crit Rev Toxicol. 2019; 10.1080/10408444.2019.1700210
20. Mandaglio-Collados, D. López-Gálvez, R. Ruiz-Alcaraz, A. J. López-García, C. Roldán, V. Lip, G. Y. H.et al., Impact of particulate matter on the incidence of atrial fibrillation and the risk of adverse clinical outcomes: A review. Sci Total Environ. 2023; 10.1016/j.scitotenv.2023.163352
21. Masroor, K. Shamsipour, M. Mehrdad, R. Fanaei, F. Aghaei, M. Yunesian, M. Exposure to ambient gaseous air pollutants and adult lung function: a systematic review. Rev Environ Health. 2023; 10.1515/reveh-2021-0135

22. Nabizadeh, R. Yousefian, F. Moghadam, V. K. Hadei, M. Characteristics of cohort studies of long-term exposure to PM(2.5): a systematic review. Environ Sci Pollut Res Int. 2019; 10.1007/s11356-019-06382-6
23. Niu, Z. Liu, F. Yu, H. Wu, S. Xiang, H. Association between exposure to ambient air pollution and hospital admission, incidence, and mortality of stroke: an updated systematic review and meta-analysis of more than 23 million participants. Environ Health Prev Med. 2021; 10.1186/s12199-021-00937-1
24. Park, J. Kim, H. J. Lee, C. H. Lee, C. H. Lee, H. W. Impact of long-term exposure to ambient air pollution on the incidence of chronic obstructive pulmonary disease: A systematic review and meta-analysis. Environ Res. 2021; 10.1016/j.envres.2020.110703
25. Pope, C. A., 3rd Coleman, N. Pond, Z. A. Burnett, R. T. Fine particulate air pollution and human mortality: 25+ years of cohort studies. Environ Res. 2020; 10.1016/j.envres.2019.108924
26. Pranata, R. Vania, R. Tondas, A. E. Setianto, B. Santoso, A. A time-to-event analysis on air pollutants with the risk of cardiovascular disease and mortality: A systematic review and meta-analysis of 84 cohort studies. J Evid Based Med. 2020; 10.1111/jebm.12380
27. Pyo, J. S. Kim, N. Y. Kang, D. W. Impacts of Outdoor Particulate Matter Exposure on the Incidence of Lung Cancer and Mortality. Medicina (Kaunas). 2022; 10.3390/medicina58091159
28. Ritz, B. Hoffmann, B. Peters, A. The Effects of Fine Dust, Ozone, and Nitrogen Dioxide on Health. Dtsch Arztebl Int. 2019; 10.3238/arztebl.2019.0881
29. Rojas-Rueda, D. Morales-Zamora, E. Alsufyani, W. A. Herbst, C. H. AlBalawi, S. M. Alsukait, R.et al., Environmental Risk Factors and Health: An Umbrella Review of Meta-Analyses. Int J Environ Res Public Health. 2021; 10.3390/ijerph18020704
30. Song, X. Hu, Y. Ma, Y. Jiang, L. Wang, X. Shi, A.et al., Is short-term and long-term exposure to black carbon associated with cardiovascular and respiratory diseases? A systematic review and meta-analysis based on evidence reliability. BMJ Open. 2022; 10.1136/bmjopen-2021-049516
31. Stieb, D. M. Berjawi, R. Emode, M. Zheng, C. Salama, D. Hocking, R.et al., Systematic review and meta-analysis of cohort studies of long term outdoor nitrogen dioxide exposure and mortality. PLoS One. 2021; 10.1371/journal.pone.0246451
32. Sun, H. Z. Yu, P. Lan, C. Wan, M. W. L. Hickman, S. Murulitharan, J.et al., Cohort-based long-term ozone exposure-associated mortality risks with adjusted metrics: A systematic review and meta-analysis. Innovation (Camb). 2022; 10.1016/j.xinn.2022.100246
33. Vodonos, A. Awad, Y. A. Schwartz, J. The concentration-response between long-term PM(2.5) exposure and mortality; A meta-regression approach. Environ Res. 2018; 10.1016/j.envres.2018.06.021
34. Wang, Q. Cao, J. Atmospheric PM(2.5) exposure and risk of ischemic heart disease: A systematic review and meta-analysis of observational studies. Perfusion. 2022; 10.1177/02676591221131485
35. Wang, T. Ma, Y. Li, R. Sun, J. Huang, L. Wang, S.et al., Trends of ischemic heart disease mortality attributable to household air pollution during 1990-2019 in China and India: an age-period-cohort analysis. Environ Sci Pollut Res Int. 2022; 10.1007/s11356-022-21770-1
36. Yang, H. Li, S. Sun, L. Zhang, X. Cao, Z. Xu, C.et al., Smog and risk of overall and type-specific cardiovascular diseases: A pooled analysis of 53 cohort studies with 21.09 million participants. Environ Res. 2019; 10.1016/j.envres.2019.01.040
37. Yang, Y. Ruan, Z. Wang, X. Yang, Y. Mason, T. G. Lin, H.et al., Short-term and long-term exposures to fine particulate matter constituents and health: A systematic review and meta-analysis. Environ Pollut. 2019; 10.1016/j.envpol.2018.12.060
38. Yang, Z. Mahendran, R. Yu, P. Xu, R. Yu, W. Godellawattage, S.et al., Health Effects of Long-Term Exposure to Ambient PM(2.5) in Asia-Pacific: a Systematic Review of Cohort Studies. Curr Environ Health Rep. 2022; 10.1007/s40572-022-00344-w
39. Yu, P. Guo, S. Xu, R. Ye, T. Li, S. Sim, M. R.et al., Cohort studies of long-term exposure to outdoor particulate matter and risks of cancer: A systematic review and meta-analysis. Innovation (Camb). 2021; 10.1016/j.xinn.2021.100143
40. Yang, S. Wang, J. Jiang, Q. He, Z. Huang, Y. Li, Z.et al., Long-term exposure to PM(2.5) and stroke: A systematic review and meta-analysis of cohort studies. Environ Res. 2019; 10.1016/j.envres.2019.108587
41. Zare Sakhvidi, M. J. Lequy, E. Goldberg, M. Jacquemin, B. Air pollution exposure and bladder, kidney and urinary tract cancer risk: A systematic review. Environ Pollut. 2020; 10.1016/j.envpol.2020.115328
42. Zhao, K. Li, J. Du, C. Zhang, Q. Guo, Y. Yang, M. Ambient fine particulate matter of diameter ≤ 2.5 μm and risk of hemorrhagic stroke: a systemic review and meta-analysis of cohort studies. Environ Sci Pollut Res Int. 2021; 10.1007/s11356-021-13074-7

43. Spangler, E. Jones, R. Livinski, A. Dawsey, S. Haley, N. Exposure to particulate matter air pollution and the incidence and mortality of GI cancers: A systematic review. Journal of Global Oncology. 2020; 10.1200/GO.20.15000
44. Atkinson, R. W. Butland, B. K. Anderson, H. R. Maynard, R. L. Long-term concentrations of nitrogen dioxide and mortality. Epidemiology. 2018; 10.1097/EDE.0000000000000847

Excluded Studies – Studies assessing only Particles **(n=71)**

1. Alexeeff, S. E. Deosaransingh, K. Van Den Eeden, S. Schwartz, J. Liao, N. S. Sidney, S.Association of Long-term Exposure to Particulate Air Pollution With Cardiovascular Events in California .JAMA Netw Open .2023;10.1001/jamanetworkopen.2023.0561
2. Awad, Y. A. Di, Q. Wang, Y. Choirat, C. Coull, B. A. Zanobetti, A.et al., Change in PM(2.5) exposure and mortality among Medicare recipients: Combining a semi-randomized approach and inverse probability weights in a low exposure population .Environ Epidemiol .2019;10.1097/ee9.0000000000000054
3. Bauleo, L. Bucci, S. Antonucci, C. Sozzi, R. Davoli, M. Forastiere, F.et al., Long-term exposure to air pollutants from multiple sources and mortality in an industrial area: a cohort study .Occup Environ Med .2019;10.1136/oemed-2018-105059
4. Bo, Y. C. Yu, T. Guo, C. Lin, C. C. Yang, H. T. Chang, L. Y.et al., Cardiovascular Mortality, Habitual Exercise, and Particulate Matter 2.5 Exposure: A Longitudinal Cohort Study .Am J Prev Med .2023;10.1016/j.amepre.2022.09.004
5. Brauer, M. Brook, J. R. Christidis, T. Chu, Y. Crouse, D. L. Erickson, A.et al., Mortality-Air Pollution Associations in Low Exposure Environments (MAPLE): Phase 2 .Res Rep Health Eff Inst .2022;
6. Brauer, M. Brook, J. R. Christidis, T. Chu, Y. Crouse, D. L. Erickson, A.et al., Mortality-Air Pollution Associations in Low-Exposure Environments (MAPLE): Phase 1 .Res Rep Health Eff Inst .2019;
7. Brown, P. E. Izawa, Y. Balakrishnan, K. Fu, S. H. Chakma, J. Menon, G.et al., Mortality Associated with Ambient PM2.5 Exposure in India: Results from the Million Death Study .Environ Health Perspect .2022;10.1289/ehp9538
8. Carlsen, H. K. Andersson, E. M. Molnár, P. Oudin, A. Xu, Y. Wichmann, J.et al., Incident cardiovascular disease and long-term exposure to source-specific air pollutants in a Swedish cohort .Environ Res .2022;10.1016/j.envres.2022.112698
9. Chen, J. Hoek, G. de Hoogh, K. Rodopoulou, S. Andersen, Z. J. Bellander, T.et al., Long-Term Exposure to Source-Specific Fine Particles and Mortality─A Pooled Analysis of 14 European Cohorts within the ELAPSE Project .Environ Sci Technol .2022;10.1021/acs.est.2c01912
10. Chen, S. Lin, X. Du, Z. Zhang, Y. Zheng, L. Ju, X.et al., Potential causal links between long-term ambient particulate matter exposure and cerebrovascular mortality: Insights from a large cohort in southern China .Environ Pollut .2023;10.1016/j.envpol.2023.121336
11. Chen, Y. Chen, R. Chen, Y. Dong, X. Zhu, J. Liu, C.et al., The prospective effects of long-term exposure to ambient PM(2.5) and constituents on mortality in rural East China .Chemosphere .2021;10.1016/j.chemosphere.2021.130740
12. Christidis, T. Erickson, A. C. Pappin, A. J. Crouse, D. L. Pinault, L. L. Weichenthal, S. A.et al., Low concentrations of fine particle air pollution and mortality in the Canadian Community Health Survey cohort .Environ Health .2019;10.1186/s12940-019-0518-y
13. Christidis, T. Pinault, L. L. Crouse, D. L. Tjepkema, M.The influence of outdoor PM(2.5) concentration at workplace on nonaccidental mortality estimates in a Canadian census-based cohort .Environ Epidemiol .2021;10.1097/ee9.0000000000000180
14. Coleman, C. J. Yeager, R. A. Pond, Z. A. Riggs, D. W. Bhatnagar, A. Arden Pope, C., 3rdMortality risk associated with greenness, air pollution, and physical activity in a representative U.S. cohort .Sci Total Environ .2022;10.1016/j.scitotenv.2022.153848
15. Coleman, N. C. Burnett, R. T. Higbee, J. D. Lefler, J. S. Merrill, R. M. Ezzati, M.et al., Cancer mortality risk, fine particulate air pollution, and smoking in a large, representative cohort of US adults .Cancer Causes Control .2020;10.1007/s10552-020-01317-w

16. Crouse, D. L. Erickson, A. C. Christidis, T. Pinault, L. van Donkelaar, A. Li, C.et al., Evaluating the Sensitivity of PM2.5-Mortality Associations to the Spatial and Temporal Scale of Exposure Assessment .Epidemiology .2020;10.1097/ede.0000000000001136
17. Crouse, D. L. Pinault, L. Balram, A. Brauer, M. Burnett, R. T. Martin, R. V.et al., Complex relationships between greenness, air pollution, and mortality in a population-based Canadian cohort .Environ Int .2019;10.1016/j.envint.2019.04.047
18. Dominici, F. Zanobetti, A. Schwartz, J. Braun, D. Sabath, B. Wu, X.Assessing Adverse Health Effects of Long-Term Exposure to Low Levels of Ambient Air Pollution: Implementation of Causal Inference Methods .Res Rep Health Eff Inst .2022;
19. Elliott, E. G. Laden, F. James, P. Rimm, E. B. Rexrode, K. M. Hart, J. E.Interaction between Long-Term Exposure to Fine Particulate Matter and Physical Activity, and Risk of Cardiovascular Disease and Overall Mortality in U.S. Women .Environ Health Perspect .2020;10.1289/ehp7402
20. Erickson, A. C. Brauer, M. Christidis, T. Pinault, L. Crouse, D. L. van Donkelaar, A.et al., Evaluation of a method to indirectly adjust for unmeasured covariates in the association between fine particulate matter and mortality .Environ Res .2019;10.1016/j.envres.2019.05.010
21. Erickson, A. C. Christidis, T. Pappin, A. Brook, J. R. Crouse, D. L. Hystad, P.et al., Disease assimilation: The mortality impacts of fine particulate matter on immigrants to Canada .Health Rep .2020;10.25318/82-003-x202000300002-eng
22. Fischer, P. H. Marra, M. Ameling, C. B. Velders, G. J. M. Hoogerbrugge, R. de Vries, W.et al., Particulate air pollution from different sources and mortality in 7.5 million adults - The Dutch Environmental Longitudinal Study (DUELS) .Sci Total Environ .2020;10.1016/j.scitotenv.2019.135778
23. Guo, C. Yu, T. Bo, Y. Lin, C. Chang, L. Y. Wong, M. C. S.et al., Long-term Exposure to Fine Particulate Matter and Mortality A Longitudinal Cohort Study of 400,459 Adults .Epidemiology .2022;10.1097/ede.0000000000001464
24. Guo, C. Yu, T. Chang, L. Y. Lin, C. Yang, H. T. Bo, Y.et al., Effects of air pollution and habitual exercise on the risk of death: a longitudinal cohort study .Cmaj .2021;10.1503/cmaj.202729
25. Guo, C. Yu, T. Lin, C. Chang, L. Y. Bo, Y. Wong, M. C. S.et al., Habitual Exercise, Air Pollution, and Pneumonia Mortality: A Longitudinal Cohort Study of Approximately 0.4 Million Adults .Am J Epidemiol .2022;10.1093/aje/kwac113
26. Hao, H. Wang, Y. Zhu, Q. Zhang, H. Rosenberg, A. Schwartz, J.et al., National Cohort Study of Long-Term Exposure to PM(2.5) Components and Mortality in Medicare American Older Adults .Environ Sci Technol .2023;10.1021/acs.est.2c07064
27. Hayes, R. B. Lim, C. Zhang, Y. Cromar, K. Shao, Y. Reynolds, H. R.et al., PM2.5 air pollution and cause-specific cardiovascular disease mortality .Int J Epidemiol .2020;10.1093/ije/dyz114
28. Higbee, J. D. Lefler, J. S. Burnett, R. T. Ezzati, M. Marshall, J. D. Kim, S. Y.et al., Estimating long-term pollution exposure effects through inverse probability weighting methods with Cox proportional hazards models .Environ Epidemiol .2020;10.1097/ee9.0000000000000085
29. Hvidtfeldt, U. A. Geels, C. Sørensen, M. Ketzel, M. Khan, J. Tjønneland, A.et al., Long-term residential exposure to PM(2.5) constituents and mortality in a Danish cohort .Environ Int .2019;10.1016/j.envint.2019.105268
30. Hwang, J. S. Hu, T. H.Later-Life Exposure to Moderate PM(2.5) Air Pollution and Life Loss of Older Adults in Taiwan .Int J Environ Res Public Health .2020;10.3390/ijerph17061873
31. Jalali, S. Karbakhsh, M. Momeni, M. Taheri, M. Amini, S. Mansourian, M.et al., Long-term exposure to PM(2.5) and cardiovascular disease incidence and mortality in an Eastern Mediterranean country: findings based on a 15-year cohort study .Environ Health .2021;10.1186/s12940-021-00797-w
32. Ji, J. S. Zhu, A. Lv, Y. Shi, X.Interaction between residential greenness and air pollution mortality: analysis of the Chinese Longitudinal Healthy Longevity Survey .Lancet Planet Health .2020;10.1016/s2542-5196(20)30027-9
33. Josey, K. P. Delaney, S. W. Wu, X. Nethery, R. C. DeSouza, P. Braun, D.et al., Air Pollution and Mortality at the Intersection of Race and Social Class .N Engl J Med .2023;10.1056/NEJMsa2300523

34. Kangas, T. Gadeyne, S. Lefebvre, W. Vanpoucke, C. Rodriguez-Loureiro, L.Are air quality perception and PM(2.5) exposure differently associated with cardiovascular and respiratory disease mortality in Brussels? Findings from a census-based study .Environ Res .2023;10.1016/j.envres.2022.115180
35. Kazemiparkouhi, F. Honda, T. Eum, K. D. Wang, B. Manjourides, J. Suh, H. H.The impact of Long-Term PM(2.5) constituents and their sources on specific causes of death in a US Medicare cohort .Environ Int .2022;10.1016/j.envint.2021.106988
36. Kim, I. S. Yang, P. S. Lee, J. Yu, H. T. Kim, T. H. Uhm, J. S.et al., Long-term fine particulate matter exposure and cardiovascular mortality in the general population: a nationwide cohort study .J Cardiol .2020;10.1016/j.jjcc.2019.11.004
37. Li, P. Wu, J. Wang, R. Liu, H. Zhu, T. Xue, T.Source sectors underlying PM(2.5)-related deaths among children under 5 years of age in 17 low- and middle-income countries .Environ Int .2023;10.1016/j.envint.2023.107756
38. Liang, F. Liu, F. Huang, K. Yang, X. Li, J. Xiao, Q.et al., Long-Term Exposure to Fine Particulate Matter and Cardiovascular Disease in China .J Am Coll Cardiol .2020;10.1016/j.jacc.2019.12.031
39. Liang, R. Chen, R. Yin, P. van Donkelaar, A. Martin, R. V. Burnett, R.et al., Associations of long-term exposure to fine particulate matter and its constituents with cardiovascular mortality: A prospective cohort study in China .Environ Int .2022;10.1016/j.envint.2022.107156
40. Liao, J. Liu, Y. Steenland, K. Pillarisetti, A. Thompson, L. M. Dey, S.et al., Child Survival and Early Lifetime Exposures to Ambient Fine Particulate Matter in India: A Retrospective Cohort Study .Environ Health Perspect .2022;10.1289/ehp8910
41. Lim, Y. H. Oh, J. Han, C. Bae, H. J. Kim, S. Jang, Y.et al., Long-term exposure to moderate fine particulate matter concentrations and cause-specific mortality in an ageing society .Int J Epidemiol .2021;10.1093/ije/dyaa146
42. Liu, L. Luo, S. Zhang, Y. Yang, Z. Zhou, P. Mo, S.et al., Longitudinal Impacts of PM(2.5) Constituents on Adult Mortality in China .Environ Sci Technol .2022;10.1021/acs.est.1c04152
43. Nilsson Sommar, J. Andersson, E. M. Andersson, N. Sallsten, G. Stockfelt, L. Ljungman, P. L.et al., Long-term exposure to particulate air pollution and black carbon in relation to natural and cause-specific mortality: a multicohort study in Sweden .BMJ Open .2021;10.1136/bmjopen-2020-046040
44. Pond, Z. A. Hernandez, C. S. Adams, P. J. Pandis, S. N. Garcia, G. R. Robinson, A. L.et al., Cardiopulmonary Mortality and Fine Particulate Air Pollution by Species and Source in a National U.S. Cohort .Environ Sci Technol .2022;10.1021/acs.est.1c04176
45. Pond, Z. A. Saha, P. K. Coleman, C. J. Presto, A. A. Robinson, A. L. Arden Pope Iii, C.Mortality risk and long-term exposure to ultrafine particles and primary fine particle components in a national U.S. Cohort .Environ Int .2022;10.1016/j.envint.2022.107439
46. Pope, C. A., 3rd Lefler, J. S. Ezzati, M. Higbee, J. D. Marshall, J. D. Kim, S. Y.et al., Mortality Risk and Fine Particulate Air Pollution in a Large, Representative Cohort of U.S. Adults .Environ Health Perspect .2019;10.1289/ehp4438
47. Sawada, N. Nakaya, T. Kashima, S. Yorifuji, T. Hanibuchi, T. Charvat, H.et al., Long-term exposure to fine particle matter and all-cause mortality and cause-specific mortality in Japan: the JPHC Study .BMC Public Health .2022;10.1186/s12889-022-12829-2
48. Shin, M. Kim, O. J. Yang, S. Choe, S. A. Kim, S. Y.Different Mortality Risks of Long-Term Exposure to Particulate Matter across Different Cancer Sites .Int J Environ Res Public Health .2022;10.3390/ijerph19063180
49. Son, J. Y. Sabath, M. B. Lane, K. J. Miranda, M. L. Dominici, F. Di, Q.et al., Long-term Exposure to PM2.5 and Mortality for the Older Population: Effect Modification by Residential Greenness .Epidemiology .2021;10.1097/ede.0000000000001348
50. Takeuchi, A. Nishiwaki, Y. Okamura, T. Milojevic, A. Ueda, K. Asakura, K.et al., Long-Term Exposure to Particulate Matter and Mortality from Cardiovascular Diseases in Japan: The Ibaraki Prefectural Health Study (IPHS) .J Atheroscler Thromb .2021;10.5551/jat.54148
51. Toyib, O. Lavigne, E. Traub, A. Umbrio, D. You, H. Ripley, S.et al., Long-term Exposure to Oxidant Gases and Mortality: Effect Modification by PM 2.5 Transition Metals and Oxidative Potential .Epidemiology .2022;10.1097/ede.0000000000001538

52. Wang, B. Eum, K. D. Kazemiparkouhi, F. Li, C. Manjourides, J. Pavlu, V.et al., The impact of long-term PM(2.5) exposure on specific causes of death: exposure-response curves and effect modification among 53 million U.S. Medicare beneficiaries .Environ Health .2020;10.1186/s12940-020-00575-0
53. Wang, Y. Du, Z. Zhang, Y. Chen, S. Lin, S. Hopke, P. K.et al., Long-term exposure to particulate matter and COPD mortality: Insights from causal inference methods based on a large population cohort in southern China .Sci Total Environ .2023;10.1016/j.scitotenv.2022.160808
54. Wang, Y. Wei, J. Zhang, Y. Guo, T. Chen, S. Wu, W.et al., Estimating causal links of long-term exposure to particulate matters with all-cause mortality in South China .Environ Int .2023;10.1016/j.envint.2022.107726
55. Wang, Y. Xiao, S. Zhang, Y. Chang, H. Martin, R. V. Van Donkelaar, A.et al., Long-term exposure to PM(2.5) major components and mortality in the southeastern United States .Environ Int .2022;10.1016/j.envint.2021.106969
56. Weichenthal, S. Pinault, L. Christidis, T. Burnett, R. T. Brook, J. R. Chu, Y.et al., How low can you go? Air pollution affects mortality at very low levels .Sci Adv .2022;10.1126/sciadv.abo3381
57. Xia, Y. Liu, Z. Hu, B. Rangarajan, S. Ah Tse, L. Li, Y.et al., Associations of outdoor fine particulate air pollution and cardiovascular disease: Results from the Prospective Urban and Rural Epidemiology Study in China (PURE-China) .Environ Int .2023;10.1016/j.envint.2023.107829
58. Yang, X. Liang, F. Li, J. Chen, J. Liu, F. Huang, K.et al., Associations of long-term exposure to ambient PM(2.5) with mortality in Chinese adults: A pooled analysis of cohorts in the China-PAR project .Environ Int .2020;10.1016/j.envint.2020.105589
59. Yang, X. Zhang, L. Chen, X. Liu, F. Shan, A. Liang, F.et al., Long-term exposure to ambient PM(2.5) and stroke mortality among urban residents in northern China .Ecotoxicol Environ Saf .2021;10.1016/j.ecoenv.2021.112063
60. Yao, Y. Liu, L. Guo, G. Zeng, Y. Ji, J. S.Interaction of Sirtuin 1 (SIRT1) candidate longevity gene and particulate matter (PM2.5) on all-cause mortality: a longitudinal cohort study in China .Environ Health .2021;10.1186/s12940-021-00718-x
61. Yazdi, M. D. Wang, Y. Di, Q. Requia, W. J. Wei, Y. Shi, L.et al., Long-term effect of exposure to lower concentrations of air pollution on mortality among US Medicare participants and vulnerable subgroups: a doubly-robust approach .Lancet Planet Health .2021;10.1016/s2542-5196(21)00204-7
62. Yitshak-Sade, M. Kloog, I. Zanobetti, A. Schwartz, J. D.Estimating the causal effect of annual PM(2.5) exposure on mortality rates in the Northeastern and mid-Atlantic states .Environ Epidemiol .2019;10.1097/ee9.0000000000000052
63. Yorifuji, T. Kashima, S. Tani, Y. Yamakawa, J. Doi, H.Long-term exposure to fine particulate matter and natural-cause and cause-specific mortality in Japan .Environ Epidemiol .2019;10.1097/ee9.0000000000000051
64. Zhang, Y.All-Cause Mortality Risk and Attributable Deaths Associated with Long-Term Exposure to Ambient PM(2.5) in Chinese Adults .Environ Sci Technol .2021;10.1021/acs.est.0c08527
65. Zhang, Y. Wang, Y. Du, Z. Chen, S. Qu, Y. Hao, C.et al., Potential causal links between long-term ambient particulate matter exposure and cardiovascular mortality: New evidence from a large community-based cohort in South China .Ecotoxicol Environ Saf .2023;10.1016/j.ecoenv.2023.114730
66. Yusuf, S. Joseph, P. Rangarajan, S. Islam, S. Mente, A. Hystad, P.et al., Modifiable risk factors, cardiovascular disease, and mortality in 155 722 individuals from 21 high-income, middle-income, and low-income countries (PURE): a prospective cohort study .The Lancet .2020;10.1016/S0140-6736%2819%2932008-2
67. Parker, J. D. Kravets, N. Vaidyanathan, A.Particulate matter air pollution exposure and heart disease mortality risks by race and ethnicity in the United States .Circulation .2018;10.1161/CIRCULATIONAHA.117.029376
68. Loop, M. S. McClure, L. A. Levitan, E. B. Al-Hamdan, M. Z. Crosson, W. L. Safford, M. M.Fine particulate matter and incident coronary heart disease in the REGARDS cohort .American Heart Journal .2018;10.1016/j.ahj.2017.11.007
69. Li, T. Zhang, Y. Wang, J. Xu, D. Yin, Z. Chen, H.et al., All-cause mortality risk associated with long-term exposure to ambient PM2.5 in China: a cohort study .The Lancet Public Health .2018;10.1016/S2468-2667%2818%2930144-0

70. Ji, J. S. Zhu, A. Lv, Y. Shi, X.Interaction between residential greenness and air pollution mortality: analysis of the Chinese Longitudinal Healthy Longevity Survey .The Lancet Planetary Health .2020;10.1016/S2542-5196%2820%2930027-9
71. Hystad, P.; Larkin, A.; Rangarajan, S.; AlHabib, K. F.; Avezum, A.; Calik, K. B. T.; Chifamba, J.; Dans, A.; Diaz, R.; du Plessis, J. L.; Gupta, R.; Iqbal, R.; Khatib, R.; Kelishadi, R.; Lanas, F.; Liu, Z.; Lopez-Jaramillo, P.; Nair, S.; Poirier, P.; Rahman, O.; Rosengren, A.; Swidan, H.; Tse, L. A.; Wei, L.; Wielgosz, A.; Yeates, K.; Yusoff, K.; Zatonski, T.; Burnett, R.; Yusuf, S.; Brauer, M.Associations of outdoor fine particulate air pollution and cardiovascular disease in 157 436 individuals from 21 high-income, middle-income, and low-income countries (PURE): a prospective cohort study .The Lancet Planetary Health .2020;10.1016/S2542-5196%2820%2930103-0

APPENDIX S3.

Nitrogen dioxide

Shape of the concentration response function between NO_2 and mortality outcomes.

We extracted information on the shape of the concentration response function for each exposure-outcome pair. Most studies used splines (natural or penalized splines) with varying degrees of freedom and compared to the linear model by model fit criteria, such as the model likelihood and BIC. All studies indicated increasing risk with increasing NO_2 levels for all pairs, while the vast majority indicated linear or supra-linear shapes, i.e. linear shapes with steeper slopes at low pollutant levels.

In the investigation of all-cause mortality, four studies [11, 13, 34, 42, 53] supported linearity, while four [15, 24, 44, 67] indicated supra-linear associations. Zhang et al. [84] indicated an approximately linear NO_2-mortality shape between 6.9–57.4 μg/m^3 with slightly gentler slopes at concentrations greater than 25 mg/m^3 and Wang et al. [76] reported a linear association at 7.4–45.0 μg/m^3 with a slightly moderated slope above 20 μg/m^3. Stafoggia et al. [67] also reported linear or supra linear shapes for circulatory, respiratory and lung cancer mortality in six administrative cohorts that were part of the ELAPSE project, similar to Brunekreef et al. [15] that additionally reported from ELAPSE on cerebrovascular, IHD, COPD and ALRI. Strak et al. [68] reporting on a pooled cohort with detailed individual covariates in ELAPSE and applying both natural splines and the SCHIF assessed the shape of the association between NO_2 and all-cause, circulatory, IHD, cerebrovascular, respiratory, COPD, and reported that associations tended to be steeper at low concentrations, levelling off at high concentrations.

Huang et al. [40] reported for all-cause, circulatory, respiratory and lung cancer mortality associations that generally showed a non-threshold linear or supra-linear shape, similar to Klompmaker et al. [47] for all-cause, circulatory, IHD, cerebrovascular, respiratory, COPD, and lung cancer mortality. Eum et al. [25] reported linear associations for long-term NO_2 exposure and all-cause and lung cancer mortality, and supra-linear for circulatory, IHD, cerebrovascular, and ALRI mortality. Fischer et al. [28] assessed the nonlinearity in the associations between NO_2 and all- cause, respiratory, circulatory and lung cancer mortality and concluded that the associations deviated significantly from linear only for circulatory disease. Zhang et al. [85] investigated mortality from all-causes, circulatory and respiratory diseases with the SCHIF and indicated a tendency for steeper slopes at above mid-range concentrations.

Dehbi et al. [25] used quartiles to explore associations between NO_2 and circulatory mortality and reported no deviations from linearity, while Yang et al. [79] reached the same conclusion by splines. For cerebrovascular mortality, Zhang et al. [86] detected a supra-linear shape with the SCHIF method. For mortality from IHD, Gan et al. [30] using quintiles supported increasing effects with increasing levels. Lim et al. [53] assessed linearity for circulatory, cerebrovascular and IHD mortality by natural splines with varying degrees of freedom and concluded on the linearity of the associations, similarly to Chen et al. [17] that assessed the associations with parabolic shapes. Klompmaker et al. [49] used splines for IHD and cerebrovascular mortality and supra-linear shapes with deviations from linearity only in the extremes of the distribution with sparse data.

Kim et al. [46] using splines concluded on the linearity for cardiovascular mortality, while for respiratory mortality associations were unclear. Naess et al. [56] investigated the association between NO_2 and circulatory and lung cancer mortality reporting that for the former risk appeared to start increasing at the level of 40 μg/m^3. Gan et al. [29] using natural cubic spline models reported ‘no discernible exposure–response trends’ for NO_2 and COPD mortality, while for ALRI mortality Liu et al. [55] again using natural cubic splines reported no deviation from linearity.

Co-pollutant adjustment

Eighteen studies adjusted for PM2.5 [11, 15, 25, 30, 33, 44, 46, 51, 53, 55, 58, 67, 68, 76, 79, 80, 84, 85]; 1 for PM10 [28]; 9 for O3 [14, 40, 51, 55, 66-68, 76, 84]; 4 for black carbon/smoke [22, 30, 68, 79]; 2 for PM2.5-10 [11, 51] and SO2 [22, 46], and one for each of SO4 [42] and CO [51].

In most of the studies, further adjustment for other pollutants did not affect the effect estimate of interest. Only five studies reported a significant impact on the estimates of interest. When Fischer et al. [28] adjusted for $PM_{10,}$ the effect estimates for NO_2 decreased, and disappeared for respiratory mortality; Lefler et al. [51] reported reduced mortality risk in models that controlled for $PM_{2.5}$; Yorifuji et al. [80] reported that in the multipollutant models with $PM_{2.5}$, the elevated risks for all- cause mortality were attenuated while, NO_2 was still associated with the elevated risk for lung cancer mortality; Strak et al. [68] reported that the associations for NO_2 with all-cause and circulatory mortality were attenuated but remained significant after adjustment for $PM_{2.5,}$ O_3 or BC; and Ji et al. [44] reported that after adjusting for $PM_{2.5}$, the association between NO_2 and mortality became protective but non statistically significantly.

Ozone

Shape of the concentration response function between O_3 and mortality

Most of the studies applied splines (natural or penalized splines) and assessed linearity by model fit criteria, with the vast majority reporting no deviations from linearity.

For annual $O_{3,}$ Hvidtfeldt et al. [41] assessed linearity for all-cause mortality by linear spline models and reported no deviation from linearity. Lim et al. [52] using splines reported that the association of O_3 and respiratory mortality was observed to be monotonic, and positively linear across the range of concentration levels in the cohort. Yuan et al. [94] used a natural cubic spline and reported a nonlinear association between long-term O_3 exposure and all-cause mortality risk was observed at 60.7-142.4 $\mu g/m^3$.

For peak/warm O_3 and mortality, Stafoggia et al. [67] and Brunekreef et al. [15] using both natural splines and the SCHIF in ELAPSE reported near linear associations along the main range of the distribution. Similarly, Strak et al. [68] in the pooled ELAPSE cohort reported generally linear or supra-linear shapes. Linearity was also supported in Liu et al. [55] by natural cubic splines for the association with ALRI mortality and in Byun et al. [90] for peak/warm O_3 and mortality, whereas associations of annual ozone leveled off after approximately 25 ppb. Shi et al. [63] using penalized splines reported nonlinearity for low levels of mean peak/warm-season O_3 (<50ppb), while Zhang et al. [95] using a restricted cubic spline detected an approximately linear relation across the concentration range 23.3–81.6 ppb.

Co-pollutant adjustment

Among studies reporting multi pollutant models, the vast majority adjusted for particles and this adjustment did not considerably influence the estimates. Kazemiparkouhi et al. [93] assessed peak/warm O_3 in models adjusting for $PM_{2.5}$ and reported that mortality risks remained significant and positive for all-cause mortality, decreased for ALRI and respiratory and increased for COPD mortality. Stafoggia et al. [67] and Brunekreef et al. [15] using the ELAPSE administrative cohorts for peak/warm O_3 reported that protective mortality risks in single pollutant models approached unity and became non-significant following adjustment for NO_2 or black carbon, and, in some instances, $PM_{2.5.}$ Similarly, Strak et al. [68] noted that the single pollutant models negative associations attenuated towards unity for all mortality outcomes, but remained statistically significant for all-cause mortality.
